# Meta-analysis of differential gene expression in lower motor neurons isolated by laser capture microdissection from post-mortem ALS spinal cords

**DOI:** 10.3389/fgene.2024.1385114

**Published:** 2024-04-16

**Authors:** William R. Swindell

**Affiliations:** Department of Internal Medicine, Division of Hospital Medicine, University of Texas Southwestern Medical Center, Dallas, TX, United States

**Keywords:** LCM, meta-analysis, matrisome, motor neuron disease, RNA-seq, SOD1, transcription factor, transcriptome

## Abstract

**Introduction:**

ALS is a fatal neurodegenerative disease for which underlying mechanisms are incompletely understood. The motor neuron is a central player in ALS pathogenesis but different transcriptome signatures have been derived from bulk analysis of post-mortem tissue and iPSC-derived motor neurons (iPSC-MNs).

**Methods:**

This study performed a meta-analysis of six gene expression studies (microarray and RNA-seq) in which laser capture microdissection (LCM) was used to isolate lower motor neurons from post-mortem spinal cords of ALS and control (CTL) subjects. Differentially expressed genes (DEGs) with consistent ALS *versus* CTL expression differences across studies were identified.

**Results:**

The analysis identified 222 ALS-increased DEGs (FDR <0.10, SMD >0.80) and 278 ALS-decreased DEGs (FDR <0.10, SMD < −0.80). ALS-increased DEGs were linked to PI3K-AKT signaling, innate immunity, inflammation, motor neuron differentiation and extracellular matrix. ALS-decreased DEGs were associated with the ubiquitin-proteosome system, microtubules, axon growth, RNA-binding proteins and synaptic membrane. ALS-decreased DEG mRNAs frequently interacted with RNA-binding proteins (e.g., FUS, HuR). The complete set of DEGs (increased and decreased) overlapped significantly with genes near ALS-associated SNP loci (*p* < 0.01). Transcription factor target motifs with increased proximity to ALS-increased DEGs were identified, most notably DNA elements predicted to interact with forkhead transcription factors (e.g., FOXP1) and motor neuron and pancreas homeobox 1 (MNX1). Some of these DNA elements overlie ALS-associated SNPs within known enhancers and are predicted to have genotype-dependent MNX1 interactions. DEGs were compared to those identified from SOD1-G93A mice and bulk spinal cord segments or iPSC-MNs from ALS patients. There was good correspondence with transcriptome changes from SOD1-G93A mice (*r* ≤ 0.408) but most DEGs were not differentially expressed in bulk spinal cords or iPSC-MNs and transcriptome-wide effect size correlations were weak (bulk tissue: *r* ≤ 0.207, iPSC-MN: *r* ≤ 0.037).

**Conclusion:**

This study defines a robust transcriptome signature from LCM-based motor neuron studies of post-mortem tissue from ALS and CTL subjects. This signature differs from those obtained from analysis of bulk spinal cord segments and iPSC-MNs. Results provide insight into mechanisms underlying gene dysregulation in ALS and highlight connections between these mechanisms, ALS genetics, and motor neuron biology.

## 1 Introduction

ALS is a fatal disease involving death of motor neurons leading to progressive muscle weakness with eventual paralysis (Feldman et al., 2022). After frustrating decades of limited progress, tremendous steps have been made in recent years, yielding multiple new drug approvals, which has finally given clinicians a choice of medications to offer ALS patients (Mead et al., 2023). Previously, the only approved disease-modifying ALS treatments were riluzole (Bensimon et al., 1994) and non-invasive ventilation (Bourke et al., 2006). More recently, however, newly approved treatments have included edaravone (Edaravone MCI-186 ALS 19 Study Group, 2017), AMX0035 (sodium phenylbutyrate and taurursodiol) (Paganoni et al., 2020) and tofersen (for patients carrying SOD1 mutations) (Miller T. M. et al., 2022). Despite these milestones, ALS remains an incurable disease with many unanswered questions regarding upper and lower motor neuron failure and its underlying pathophysiology. Existing data has supported diverse pathomechanisms related to processes shared by other neurodegenerative conditions, which include defects in DNA/RNA, protein aggregation, proteostasis, neuronal networks, cytoskeleton structure, energy metabolism, inflammation and cell death pathways (Wilson et al., 2023). For all these processes, moreover, disease mechanisms are multilayered and influenced by specific genetic mutations, background disease-modifying genetic variants, environmental factors, and finally, by the ageing process itself (Akçimen et al., 2023). Increasingly, it has been appreciated that unraveling this complexity will require guidance from large-scale hypothesis-generating investigations that fully leverage multi-omics technologies (Morello et al., 2020). For this work, motor neurons, although a difficult cell type to isolate and study in humans, represent an epicenter of the ALS disease cascade and thus warrant special focus in efforts to understand disease mechanisms.

The evaluation of ALS motor neuron pathology has included studies of post-mortem CNS tissues as well as motor neurons differentiated from induced pluripotent stem cells (iPSCs) (Dimos et al., 2008). This latter approach has been implemented on a large scale as part of the Answer ALS project, which has now generated >1000 iPSC lines from ALS patients and controls along with transcriptome data from hundreds of these lineages (Workman et al., 2023). This approach offers the potential to obtain a functional genomic signature for ALS, based on motor neurons, using a much larger sample size than has been practical in post-mortem tissue studies. Results from this strategy, however, have been puzzling. An initial transcriptome comparison of 341 ALS and 92 control (CTL) iPSC lines from the Answer ALS project identified only 13 differentially expressed genes (DEGs) (Workman et al., 2023). Similarly, meta-analysis of 15 transcriptome datasets generated from iPSC-derived motor neurons identified only 43 genes differentially expressed between ALS (*n* = 323) and CTL (*n* = 106) samples (Ziff et al., 2023). Much larger numbers of differentially expressed genes, however, have been obtained from post-mortem tissue studies. A recent analysis, for example, used bulk tissue RNA-seq to compare post-mortem spinal cord segments between ALS (*n* = 154) and CTL subjects (*n* = 49), and identified 7349 and 4694 DEGs in the cervical and lumbar cord regions, respectively (FDR <0.05) (Humphrey et al., 2023). It is unclear why this degree of differential expression has not been replicated by iPSC transcriptome studies. On the one hand, bulk tissue RNA-seq may capture transcriptome changes stemming from multiple cell types, including microglia and astrocytes, leading to more profound differential expression (Yadav et al., 2023). Alternatively, iPSC-MNs may not be sufficiently differentiated to model the disease state of postmitotic motor neurons in ALS (Ho et al., 2016), leading to loss or diminution of *in situ* transcriptome differences that separate ALS from CTL motor neurons.

The application of laser capture microdissection (LCM) to post-mortem CNS tissue offers an approach for evaluating *in situ* motor neurons and their distinctive transcriptome signature in ALS patients (Jiang et al., 2005; Cox et al., 2010; Rabin et al., 2010; Kirby et al., 2011; Highley et al., 2014; Cooper-Knock et al., 2015; Ladd et al., 2017; Krach et al., 2018; Nizzardo et al., 2020). LCM allows RNA to be isolated from a motor neuron-enriched cellular pool, with less contribution from surrounding cell types as would occur with bulk tissue analysis (EmmertBuck et al., 1996). These studies have been informative but sample sizes have been limited and studies have varied with respect to laboratory methods, expression profiling platforms and statistical methods. Additionally, most analyses have focused on newly generated data only, without comparisons to previously published data. Conclusions have thus varied, although functional annotations of ALS dysregulated genes in LCM studies have related to cytoskeleton structure, RNA metabolism, RNA splicing and phosphatidylinositol-3 kinase signaling (Jiang et al., 2005; Cox et al., 2010; Rabin et al., 2010; Kirby et al., 2011; Highley et al., 2014; Cooper-Knock et al., 2015; Ladd et al., 2017; Krach et al., 2018; Nizzardo et al., 2020). There has been one meta-analysis study of three LCM-generated datasets comparing motor neuron expression between ALS and CTL samples, which identified 206 DEGs in common among datasets (*p* < 0.05, FC ≥ 2 or FC ≤ 0.50) (Lin et al., 2020). This study, however, did not include RNA-seq datasets, did not utilize a formal meta-analysis model, and only included studies of sporadic but not familial ALS patients.

This study reports findings from meta-analysis of six datasets in which LCM-dissected motor neurons were targeted for genome-wide expression profiling (Cox et al., 2010; Rabin et al., 2010; Kirby et al., 2011; Highley et al., 2014; Cooper-Knock et al., 2015; Krach et al., 2018; Nizzardo et al., 2020). The study incorporates both microarray and RNA-seq data and applies a random effects meta-analysis model to define differentially expressed genes (DEGs). The identified DEGs are compared to those linked to ALS by genome-wide association (GWA) studies as well as to genes altered in iPSC-derived motor neurons (Workman et al., 2023; Ziff et al., 2023) and bulk spinal cord segments (Humphrey et al., 2023). A novel approach utilizing nearest neighbor statistics is applied to identify putative transcription factor (TF) binding sites near DEGs and to further identify ALS-associated SNPs that may disrupt such sites. Results from this study define a high-confidence ALS transcriptome signature that provides a window into the *in situ* properties of motor neurons, *cis* regulatory mechanisms, and connections between ALS genetics and motor neuron biology.

## 2 Materials and methods

Table 1 lists datasets incorporated into the meta-analysis. Preprocessing, normalization and differential expression analysis steps for each dataset are described below. All samples correspond to RNA pools from LCM-dissected lower motor neurons from post-mortem spinal cord segments. Prior to performing the meta-analysis, DEGs were identified with respect to each dataset individually using the same statistical thresholds (i.e., FDR <0.10 with FC > 1.50 or FC < 0.67).

**TABLE 1:** Meta-analysis datasets. The table lists the 6 expression datasets incorporated into the meta-analysis. Sample sizes are listed for each dataset along with the number of genes included in each differential expression analysis. The final two columns list the number of differentially expressed genes identified with respect to each dataset individually. See footnotes for further details.

GEO series	*n* (ALS)	*n* (CTL)	Gene count^a^	ALS-increased^b^	ALS-decreased^c^
GSE18920^d^	12	10	12064	3	0
GSE19332^e^	6	7	9629	0	0
GSE56500^d^	6	6	12112	98	411
GSE68605^e^	8	3	7687	17	12
GSE76220^f^	13	7	8759	0	0
GSE115130^g^	7	4	3293	93	82

^a^ Number of protein-coding genes included in differential expression analyses.

^b^ Number of ALS-increased genes identified (FDR <0.10, FC > 1.50).

^c^ Number of ALS-decreased genes identified (FDR <0.10, FC < 0.67).

^d^ Affymetrix Human Exon 1.0 ST, array.

^e^ Affymetrix Human Genome U133 Plus 2.0 array.

^f^ Illumina Genome Analyzer II.

^g^ Illumina HiSeq 2000.

### 2.1 GSE18920

The dataset consisted of 22 samples from 12 sporadic ALS subjects (6 males, 6 females) and 10 CTL subjects (8 males, 2 females) (Rabin et al., 2010). The average age of ALS and CTL subjects was 66.4 (±3.2) and 72.8 (±3.3) years, respectively (Rabin et al., 2010). The average post-mortem sample collection interval was 4.4 (±0.33) and 5.1 (±1.05) hours for ALS and CTL subjects, respectively (Supplementary Figure S1A) (Rabin et al., 2010). RNA expression profiling was performed using the Affymetrix Human Exon 1.0 ST array platform. No prominent spatial artifacts were identified from microarray pseudo-images (Supplementary Figures S1B–W). Raw signal intensities had a similar distribution for each array, with the exception of CTL-1 (GSM468741), which had an increased frequency of low-intensity probes (Supplementary Figure S1X).

Quality control metrics were calculated using probe level models (PLM) (R package: oligo, function: fitProbeLevelModel) (McCall et al., 2011). PLM residuals were similar among arrays (Supplementary Figure S1Y). One sample (CTL-1) had a relatively increased normalized unscaled standard error (NUSE) median and interquartile range (Supplementary Figures S1Z, S1AA) (McCall et al., 2011). Likewise, CTL-1 had a relatively increased relative log expression (RLE) median (Supplementary Figure S1BB) although the RLE IQR was similar among arrays (Supplementary Figure S1CC) (McCall et al., 2011). Microarray normalization was performed using robust multichip average (RMA), including background subtraction, quantile normalization and median-polish summarization (R package oligo, function: rma) (Irizarry et al., 2003). RMA generated normalized intensity estimates for 22011 meta-probesets (MPS), of which 14303 could be unambiguously assigned to a single human gene symbol. In some cases, a human gene was represented by more than one MPS. The 14303 MPS were therefore filtered to include only one MPS per human gene, preferentially retaining the MPS for which mean RMA-normalized expression was highest across samples. This yielded 14141 MPS uniquely representing the same number of human genes, which were further filtered to include only 13611 MPS assigned to a protein-coding gene.

Since quality concerns were noted for CTL-1, MPS were excluded if expression of the CTL-1 sample was the highest or lowest among all samples, with a z-score-normalized expression estimate greater than 3 or less than −3. This yielded 13501 MPS. Of these, those with detectable expression in at least 15% of samples were retained (i.e., ≥4 of 22 samples), with detectable expression defined as an RMA-normalized expression value above the 15th percentile. This yielded 12064 MPS upon which differential expression analyses were based.

Linear models for differential expression analyses were estimated by generalized least squares using both sex and phenotype (ALS vs. CTL) as variables (R package: limma, function: lmFit) (Smyth, 2004). Sex was included in models since, as noted above, the proportion of males and females was dissimilar between ALS and CTL subjects. Moderated t-statistics were calculated using empirical Bayes shrinkage of the standard errors (R package: limma, function: eBayes) (Smyth, 2004). To control for multiple hypothesis testing among the 12064 MPS, raw *p*-values were FDR-corrected using the Benjamini-Hochberg method (Benjamini and Hochberg, 1995).

### 2.2 GSE19332

The dataset consisted of 13 samples from 6 ALS and 7 CTL subjects (Table 1). Of the 6 ALS samples, 3 were from subjects carrying *CHMP2B* mutations (Cox et al., 2010) while 3 were from subjects carrying *SOD1* mutations (Kirby et al., 2011). For brevity, these data are referenced by the accession GSE19332 throughout this manuscript. However, CTL samples were submitted under two GEO accessions (GSE19332 and GSE20589), whereas samples for ALS patients with *CHMP2B* or *SOD1* mutations are available under GSE19332 and GSE20589, respectively.

Gene expression was profiled using the Affymetrix Human Genome U133 Plus 2.0 array. Inspection of microarray pseudo-images did not reveal prominent spatial artifacts (Supplementary Figures S2A–M). Raw signal intensities showed good correspondence among samples (Supplementary Figure S2N). Probe-level model residual distributions were consistent among samples (Supplementary Figure S2O) (R package: oligo, function: fitProbeLevelModel) (McCall et al., 2011). Sample CTL-7 (GSM480310) had relatively elevated NUSE median and IQR values (Supplementary Figures S2O, P) but was otherwise unremarkable with respect to RLE median and IQR (Supplementary Figures S2R, S) (McCall et al., 2011). Expression summary scores for 54675 probe sets (PS) were calculated using RMA normalization (R package: affy, function: justRMA) (Irizarry et al., 2003). To limit redundancy (Li et al., 2008), a single PS was chosen to represent each human gene. To choose a representative PS, those PS with less specific probe sequences that may cross-hybridize with non-targeted transcripts (i.e., Affymetrix identifiers with an _x_ or _s_ suffix) were preferentially excluded. If there remained multiple PS after applying this criterion, the PS for which mean RMA-normalized expression was highest among the 13 samples was chosen as the representative.

The above steps yielded 20824 PS uniquely assigned to the same number of human genes, of which 17892 were assigned to a protein-coding gene. The MAS 5.0 algorithm was applied to determine which PS had detectable expression in each sample (R package: affy, function: mas5calls) (Liu et al., 2002). Differential expression testing was then performed using a subset of 9629 PS with detectable expression in at least 15% of samples (i.e., at least 2 of 13). Differential expression testing was performed as described above using general linear models and moderated t-statistics (R package: limma, functions: lmFit, eBayes) (Smyth, 2004). To correct for multiple hypothesis testing among the 9629 PS, raw *p*-values were FDR-corrected using the Benamini-Hochberg method (Benjamini and Hochberg, 1995).

### 2.3 GSE56500

The dataset consisted of 12 samples from 12 subjects, including 6 ALS subjects (4 males, 2 females) and 6 CTL subjects (5 males, 1 female) (Highley et al., 2014). The average age of ALS and CTL subjects was 60.2 (±3.5) and 61.7 (±3.9) years, respectively. Of the 6 ALS patients, 3 had sporadic disease and 3 carried *C9ORF72* mutations. Expression profiling was performed using the Affymetrix Human Exon 1.0 ST array. Inspection of microarray pseduo-images did not reveal prominent spatial artifacts (Supplementary Figures S3A–L). Raw signal intensities differed among samples although no single sample emerged as an outlier (Supplementary Figure S3M). Likewise, the distribution of PLM residuals, NUSE median/IQR and RLE median/IQR did not identify any problematic samples (Supplementary Figures S3N–R).

Data normalization and processing steps mirrored those described above for GSE18920, which utilized the same microarray platform. RMA normalization was performed (R package oligo, function: rma) (Irizarry et al., 2003) and subsequent filtering steps yielded 13617 MPS uniquely corresponding to the same number of protein-coding human genes. Of these, 12112 MPS had detectable expression in at least 15% of the 12 samples (i.e., at least 2 of the 12 samples). Differential expression testing was performed on these 12112 MPS using generalized linear models with moderated t-statistics (R package: limma, functions: lmFit, eBayes). To correct for multiple hypothesis testing among the 12112 MPS, raw *p*-values were FDR-corrected using the Benjamini-Hochberg method (Benjamini and Hochberg, 1995).

### 2.4 GSE68605

The dataset included 11 samples from 8 ALS (3 males, 5 females) and 3 CTL (1 male, 2 females) subjects (Cooper-Knock et al., 2015). The average age of ALS and CTL samples was 62 (±1.5) and 60 (±4.0) years, respectively (Cooper-Knock et al., 2015). Expression profiling was performed using the Affymetrix Human Genome U133 Plus 2.0 array. Inspection of microarray pseudo-images revealed minor spatial artifacts for some samples, including CTL-1 (GSM1676861), CTL-2 (GSM1676862) and ALS-2 (GSM1676854) (Supplementary Figures S4A–K). However, raw signal intensity distributions were similar among samples (Supplementary Figure S4L) and no consistent outlier pattern was evident with respect to PLM residuals, NUSE median/IQR and RLE median/IQR (Supplementary Figures S4M–Q). Preprocessing and normalization steps were similar to those described above for GSE19332, which utilized the same array platform. RMA normalization generated signals for 17892 PS (R package: affy, function: justRMA) (Irizarry et al., 2003), of which 7687 had detectable expression in at least 15% of samples (i.e., at least 2 of 11 samples). Differential expression testing for these 7687 PS was performed using generalized linear models with moderated t-statistics (R package: limma, functions: lmFit, eBayes) (Smyth, 2004). To control for multiple hypothesis testing among the 7687 PS, raw *p*-values were FDR-adjusted using the Benjamini-Hochberg method (Benjamini and Hochberg, 1995).

### 2.5 GSE76220

The initial dataset consisted of 21 samples (Krach et al., 2018). However, fastq files downloaded for CTL-3 (GSM1977029) and CTL-8 (GSM1977034) were identical. The latter sample CTL-8 (GSM1977034) was therefore dropped from the analysis, after which there remained 20 samples from 13 ALS (9 males, 4 females) and 7 CTL (5 males, 2 females) subjects. The average age of ALS and CTL subjects was 63.3 (3.8) and 74.8 (2.8) years, respectively. The average post-mortem sample collection interval was 4.2 h (Supplementary Figure S5A) and the average RNA integrity number (RIN) was 5.7 (Supplementary Figure S5B).

Sequencing reads were generated using the Illumina Genome Analyzer II. Fastq file statistics were calculated using the FastQC software (Andrews, 2010). Prior to read filtering, there was an average of 28.7 million reads per sample, with no less than 11.3 million reads in any one sample (Supplementary Figure S5C). Read correction was performed using Rcorrector (Song and Florea, 2015) and adaptor and quality trimming was carried out using TrimGalore (Krueger, 2015). Sequences matching rRNA sequences were filtered out using the bbduk.sh shellscript contained within the BBTools software suite (Bushnell, 2018). Following these filtering steps, there was an average of 26.5 million reads per sample, with all samples having at least 10.4 million reads (Supplementary Figure S5D). Quality-filtered reads were mapped to the UCSC GRCh38/hg38 genome sequence using the STAR aligner (Dobin et al., 2013). Quantification of gene counts was performed with StringTie (Pertea et al., 2015). This protocol mapped 94.4% of reads on average (Supplementary Figure S5E), with 99.0% of reads on average assigned to intragenic sequence (Supplementary Figure S5F). An average of 87.1% of reads were assigned to exons (Supplementary Figure S5G) and only 0.4% of reads on average were assigned to ribosomal sequence (Supplementary Figure S5H).

Gene counts were generated for 18220 protein-coding genes. A gene was considered to have detectable expression if there was at least one mapped read and the Fragments Per Kilobase of transcript per Million mapped reads (FPKM) was greater than 0.3 (Ramsköld et al., 2009; Hart et al., 2013). Initially, analyses were performed on 10921 genes meeting these criteria for expression in at least 15% of samples (i.e., at least 3 of 20 samples; Supplementary Figure S6). However, inspection of MA plots revealed a relationship between FC estimates and gene abundance (Supplementary Figure S6D). This relationship was no longer apparent when the analysis was performed using 8759 genes with detectable expression in at least 50% of samples (i.e., at least 10 of 20 samples). This more stringent inclusion criteria was thus applied. For differential expression testing, raw gene counts were normalized using Trimmed Mean of M-values (TMM) (Robinson and Oshlack, 2010) and transformed to log-counts per million using the voom algorithm (Law et al., 2014) (R package: edgeR, functions: calcNormFactors, voom). Differential expression testing was then performed using precision weights estimated from the global mean-variance trend (Law et al., 2014), followed by fitting of generalized linear models and calculation of moderated t-statistics in a manner similar to that described above for microarray datasets (Smyth, 2004). To control for multiple hypothesis testing among the 8759 genes, raw *p*-values were FDR-corrected using the Benjamini-Hochberg algorithm (Benjamini and Hochberg, 1995).

### 2.6 GSE115130

The initial dataset consisted of 28 samples with sequencing reads generated from the Illumina HiSeq 2000 platform, which had been uploaded under three GEO series accessions (GSE115130, GSE76514, GSE93939) (Nichterwitz et al., 2016; Nizzardo et al., 2020). Throughout this manuscript, for brevity, only the GEO accession linked to ALS samples (GSE115130) is mentioned when referring to these data. The 28 samples included 10 from 7 ALS subjects and 18 from 10 CTL subjects. Of the 18 CTL, samples, there were six sample pairs for which fastq files were identical or nearly identical (GSM2027414:GSM3615509, GSM2027415:GSM3615511, GSM2027416:GSM3615501, GSM2027417:GSM3615503, GSM2027418:GSM3615504, GSM2027419:GSM3615505). Six of the duplicated samples were thus dropped from further analysis (GSM3615509, GSM3615511, GSM3615501, GSM3615503, GSM3615504, GSM3615505). This yielded a filtered dataset with 22 samples overall, including 10 ALS samples from 7 subjects and 12 CTL samples from 9 subjects.

There was an average of 6.6 million reads per sample prior to filtering (Supplementary Figure S7A). Read processing and filtering steps described above were applied, yielding an average of 4.8 million reads per sample (Supplementary Figure S5B). Quality-filtered reads were then mapped to the UCSC GRCh38/hg38 genome sequence using the STAR/StringTie pipeline described above (Dobin et al., 2013; Pertea et al., 2015). An average of 80.2% of reads were mapped (Supplementary Figure S7C), with an average of 97.1% of mapped reads assigned to intragenic sequence (Supplementary Figure S7D) and an average of 77.6% of mapped reads assigned to exonic sequence (Supplementary Figure S7E). Only 1.2% of reads on average mapped to ribosomal sequences (Supplementary Figure S7F). Nine samples were excluded from further analysis because the post-filter read count was less than 4 million reads, yielding 13 samples from 11 subjects. For cases in which more than one sample was available from the same subject, the sample with highest post-filter read count was retained. Following these steps, the dataset contained 7 ALS samples from unique subjects (GSM2465366, GSM2465372, GSM2465365, GSM2465370, GSM2465364, GSM2465363, GSM2465369) and 4 CTL samples from unique subjects (GSM3615508, GSM2027417, GSM2027419, GSM3615507).

Gene counts were quantified for 18220 protein-coding genes. Given that sequencing depth was limited, differential expression testing was performed for only 3293 genes with detectable expression in all 11 samples, where detectable expression was defined as indicated above (i.e., at least one mapped read with FPKM ≥0.30) (Ramsköld et al., 2009; Hart et al., 2013). Differential expression analysis was performed using the voom-limma approach described above (Law et al., 2014), and the Benjamini-Hochberg method was used to correct for multiple hypothesis testing among the 3293 genes (Benjamini and Hochberg, 1995).

### 2.7 Meta-analysis

Differential expression effect size was calculated based on the standardized mean difference (SMD) using the Hedge’s g estimator (R package: effsize, function: cohen.d) (Hedges, 1981; Hedges and Olkin, 2014). For microarray datasets (GSE18920, GSE19332, GSE56500, GSE68605), SMD was estimated using log_2_ scale RMA-normalized expression values (Irizarry et al., 2003). For RNA-seq datasets (GSE76220, GSE115130), SMD was estimated using TMM-normalized gene counts transformed to log_2_-counts per million (Robinson and Oshlack, 2010; Law et al., 2014), such that SMD estimates from both the array and RNA-seq datasets were calculated using a log_2_-based expression scale. SMD was only calculated for genes meeting the above-stated criteria for having detectable expression in a sufficient number of samples. If these criteria were not met for a given study, the overall SMD meta-estimate was based on fewer than 6 individual study estimates. Given this approach, an SMD meta-estimate was calculated for 9882 protein-coding genes for which at least 3 study-specific estimates were available (3 estimates for 2680 genes, 4 for 2776 genes, 5 for 2576 genes, and 6 for 1850 genes). Genes with 2 or fewer study-specific estimates were excluded from the meta-analysis. The SMD meta-estimate was calculated using a random effects meta-analysis model with inverse variance weighting (R package: meta, function: metacont) (Schwarzer, 2007). DEGs were identified based upon an FDR-adjusted *p*-value less than 0.10 with meta-SMD estimate greater than 0.80 or less than −0.80. The FDR threshold of <0.10 is consistent with prior transcriptome meta-analysis studies (Burguillo et al., 2010; Huan et al., 2016; Vora et al., 2018; Medina et al., 2023). The meta-SMD threshold of ±0.80 has been viewed as representing a “large” treatment effect in meta-analysis studies (Cohen, 2013), with SMD values exceeding 0.80 in absolute value corresponding to 53% or less overlap between two comparison groups (Andrade, 2020).

### 2.8 Analysis of over-represented gene annotations

ALS-increased and -decreased DEGs were evaluated to assess for overrepresentation of annotations related to processes, functions, cell components or pathways (Maleki et al., 2020). The “universe” of background genes in all analyses was limited to the 9882 motor neuron-expressed protein-coding genes included in the meta-analysis. Enrichment of Gene Ontology (GO) biological process (BP), molecular function (MF) and cell component (CC) terms was assessed using a conditional hypergeometric test (R package: Gestates; function: hyperGTest) (Falcon and Gentleman, 2007). Fisher’s exact test was used to evaluate for enrichment with respect to terms from the Kyoto Encyclopedia of Genes and Genomes (KEGG) (Kanehisa et al., 2016), WikiPathways (Agrawal et al., 2024), Reactome (Fabregat et al., 2018), Disease Ontology (DO) (Kibbe et al., 2015), Pathway Commons (Rodchenkov et al., 2020), Medical Subject Headings (MeSH) (Yu, 2018), Drug Signatures Database (DSigDB) (Yoo et al., 2015), Molecular Signatures Database (MSigDB) (Liberzon et al., 2015) databases (R packages clusterProfiler (Yu et al., 2012), ReactomePA (Yu and He, 2016), DOSE (Yu et al., 2015), paxtoolsr (Luna et al., 2016), meshes (Yu, 2018) and msigdbr (Dolgalev, 2022)). Fisher’s exact test was also used to determine if any non-coding RNA targets were enriched among DEGs, based on ncRNA-target associations provided by the LncRNA2Target (Cheng et al., 2019), miRTarBase (Chou et al., 2018), miRDB (Chen and Wang, 2020) and TargetScan (Agarwal et al., 2015; McGeary et al., 2019). Likewise, Fisher’s exact test was used to identify protein interaction partners frequently interacting with mRNAs linked to DEGs, based on RNAInter database annotations (Kang et al., 2022).

Networks were generated to illustrate topological relationships among GO BP terms enriched with respect to ALS-increased and -decreased DEGs, respectively. In each case, the network was generated by drawing connections between enriched GO BP terms for which associated genes overlapped by at least 50% (R package: igraph) (Csardi and Nepusz, 2006). Groups of similar enriched GO BP terms were identified to color-code the network based on broader GO categorizations, with GO BP term groups identified using hierarchical cluster analysis with Euclidean distance and average linkage. For this analysis, the distance between two GO BP terms *A* and *B* was calculated using the distance metric 1—max (*p*_A_, *p*_B_), where *p*_A_ and *p*_B_ represent the proportions of overlapping genes associated with terms *A* and *B*, respectively. Groups of similar GO BP terms associated with overlapping gene sets were then defined based on the resulting dendrogram using variable height branch pruning (R package: dynamicTreeCut; function: cutreeDynamicTree) (Langfelder et al., 2008).

### 2.9 Protein-protein interaction network

Protein-protein interactions were obtained from the STRING database (version 12.0) (Szklarczyk et al., 2023). Only high-confidence protein interactions were considered (confidence score ≥0.700). Of the 1850 genes having detectable expression in each of the 6 meta-analysis datasets, 1343 were linked to a protein having at least one high-confidence STRING database interaction, with an overall total of 4449 interactions among the 1343 proteins. The 1343 proteins were assigned to 12 subgroups based on average linkage hierarchical clustering (Euclidean distance), using a distance matrix with entries calculated from the confidence score between protein pairs (i.e., 1—confidence score) (R package: dynamicTreeCut, function: cutreeDynamicTree) (Langfelder et al., 2008). The dominant functional theme for each protein group was determined based upon the most significant GO BP term overrepresented in each group (R package: GOstats) (Falcon and Gentleman, 2007). The Kamada-Kawai algorithm was used to generate the protein-protein interaction network layout (R package: igraph, function: layout_with_kk) (Kamada and Kawai, 1989; Csardi and Nepusz, 2006). Network vertices and edges were color-coded based upon protein subgroup or SMD estimate.

### 2.10 Motif analyses

Motif analyses were performed using the Jaspar Core vertebrate collection, consisting of 841 non-redundant experimentally-determined TF binding sites (Rauluseviciute et al., 2023). The 841 position frequency matrices (PFMs) were converted to position probability matrices (PPMs) using a pseudocount of 0.8 (Nishida et al., 2009). Genome analyses were performed using the telomere-to-telomere (T2T-CHM13) human genome sequence with annotated sequence coordinates for 19969 protein-coding genes (Nurk et al., 2022). The T2T genome sequence was used for motif analysis because it corrects assembly gaps from the GRCh38 genome, providing an additional 200 million base pairs with improved accuracy and coverage of complex repetitive genomic regions (Nurk et al., 2022). For each T2T-CHM13 chromosome, PPMs were converted to position weight matrices (PWMs) based on background nucleotide frequencies calculated for that chromosome (Wasserman and Sandelin, 2004). The chromosome sequence was scanned for PWM matches using a multinomial model with Dirichlet conjugate prior (R package: Biostrings, function: matchPWM) (Wasserman and Sandelin, 2004). Background nucleotide frequencies were calculated for each chromosome separately. A PWM model was considered to match a given locus if the correspondence score exceeded 80% of the theoretical PWM maximum score (Wasserman and Sandelin, 2004). Further analyses were performed on 798 motifs having at least one PWM match to each chromosome.

Transcription factors can regulate gene expression through binding at enhancer sites located far from target genes, with regulatory interactions spanning distances extending far beyond the upstream windows of 1 kb, 2 kb or 5 kb often recognized as gene promoter regions (Shlyueva et al., 2014; Chen et al., 2020). The current study therefore used a threshold-free nearest neighbor statistic to test for associations between PWM match locations and DEG transcription start sites (TSSs). For each PWM model and each protein-coding gene, the nearest neighbor (NN) distance was calculated, defined as the distance between the gene’s TSS and the closest PWM match on the chromosome. For these calculations, the most downstream TSS was used for genes associated with multiple isoforms with alternative TSS locations. NN distances among genes approximated a Poisson distribution for each PWM model. The distance ratio was defined as NN_*FG*_/NN_*BG*_, where NN_FG_ represents the average NN distance among foreground genes (i.e., DEGs), and NN_*BG*_ represents the average NN distance among background genes (i.e., all other genes included in the meta-analysis). A ratio less than one indicates that a PWM model has genome matches closer on average to the TSS of foreground genes. To evaluate the significance of this ratio, log_10_-transformed NN distances were compared between foreground and background genes using Welch’s two-sample two-tailed *t*-test (R function: t.test). To correct for multiple hypothesis testing among the 798 motifs, raw *p*-values were FDR-corrected using the Benjamini-Hochberg method (Benjamini and Hochberg, 1995). ALS-associated PWMs (AAPs) were defined as PWM models for which the average NN distance between genomic match locations and FG gene TSSs is lower (FDR <0.10) than that of BG gene TSSs.

### 2.11 ALS-associated genes and SNP loci

SNP loci previously associated with ALS by genome-wide association (GWA) studies were identified from the NHGRI-EBI GWAS catalog (file: All associations v1.0) (Sollis et al., 2023). SNP loci were identified based on 7 catalog traits, including “ALS,” “ALS (age of onset),” “ALS (C9orf72 mutation interaction),” “ALS (sporadic),” “ALS in C9orf72 mutation negative individuals,” “ALS in C9orf72 mutation positive individuals” and “Rapid functional decline in sporadic ALS.” There were 175 unique SNPs associated with these traits, which were together associated with 323 genes based on reported, mapped, upstream and downstream catalog genes. Based on the 175 SNPs, a broader set of 2639 SNPs was identified by including those in linkage disequilibrium with the initial set of 175 (*R* ^2^ ≥ 0.90) (R package: LDlinkR, function: LDproxy) (Myers et al., 2020). For each SNP, the nearest human gene was identified, leading to an additional 60 ALS-associated genes for an overall total of 383. Of these 383 genes, 204 were protein-coding motor neuron-expressed genes included in the meta-analysis. The location of each SNP was evaluated to determine if it was within an enhancer region, based on annotations available from GeneHancer (Fishilevich et al., 2017), the ENCODE Project (Consortium, 2012) and ORegAnno (Lesurf et al., 2016). Both alleles of each SNP were evaluated to determine if there was differential correspondence to any of the PWM models described above. A genotype-dependent PWM match was defined as one for which the correspondence score at a SNP-overlying locus exceeded 80% of the theoretical maximum score of that PWM for one allele but not the other (R package: Biostrings, function: matchPWM) (Swindell et al., 2015).

### 2.12 Comparison to SOD1-G93A mouse model of ALS

Expression changes seen in ALS patient motor neurons were compared to those in LCM-dissected motor neurons from SOD1-G93A mice (Gurney et al., 1994). Two datasets were evaluated (GSE10953 and GSE46298). The first (GSE10953) compared G93A to CTL mice on the C57BL6/J background at three time points, corresponding to presymptomatic (day 60), symptomatic (day 90) and late (day 120) stages of disease progression (*n* = 3 for each treatment/time combination) (Ferraiuolo et al., 2007). The second (GSE46298) evaluated mice from two strains (C57BL6/J and 129Sv), with comparisons between G93A and CTL mice at 4 time points corresponding to presymptomatic (day 56), onset (C57: day 133, 129Sv: day 101), symptomatic (C57: day 152, 129Sv: day 111) and endstage disease (C57: day 160, 129Sv: day 121) (*n* = 4 for each treatment/time/strain combination) (Nardo et al., 2013).

The GSE10953 dataset was generated using the Affymetrix Mouse Expression 430A array platform. Raw CEL files were normalized using the RMA algorithm (R library: affy, function: justRMA) (Irizarry et al., 2003) yielding signals for 22690 PS representing 13091 unique genes, of which 12765 were protein-coding. Of these, a subset of 11223 genes could be uniquely matched to a human orthologue based upon homology information from the Mouse Genome Database (Blake et al., 2021). Inspection of microarray pseudoimages revealed minor spatial artifacts on two arrays (Supplementary Figures S8Q, R) and these same arrays differed with respect to their raw intensity distributions and some quality-control metrics (Supplementary Figures S8S–X). However, none of the 11223 genes with human orthologues had outlying expression values for these two array samples (i.e., |z-score| < 3 for all genes). Additionally, no single array emerged as an outlier on cluster and PC analyses (Supplementary Figures S9A, D). There was minimal separation between G93A and CTL samples on cluster and PC analyses (Supplementary Figures S9A, D), although G93A and CTL samples did differ significantly with respect to the third PC axis (*p* < 0.05, two-sample two-tailed *t*-test; Supplementary Figure S9G).

The GSE46298 dataset was generated using the Affymetrix Mouse Genome 430 2.0 array platform. RMA normalization as above yielded signals for 45101 PS corresponding to 20493 unique mouse genes of which 17292 were protein-coding. Of the 17292 protein-coding genes, 14741 were uniquely matched to a human orthologue (Mouse Genome Database) and thus included in further analyses. No prominent spatial artifacts were detected on microarray pseudoimages and quality-control metrics were within an acceptable range for all microarray samples (Supplementary Figures S10, 11). For both strains, there was good separation between G93A and CTL samples in cluster and PC analyses (Supplementary Figures S9B, C, E, F) and there was a significant difference between G93A and CTL samples with respect to the first and/or second PC axis (*p* < 0.05, two-sample two-tailed *t*-test; Supplementary Figures S9H, I).

Differential expression testing (G93A vs. CTL) was performed as described above using general linear models and moderated t-statistics (R package: limma, functions: lmFit, eBayes) (Smyth, 2004). Testing was performed only for those protein-coding genes having human orthologues and detectable expression in at least one of the 6–8 samples involved in each comparison. Genes with detectable expression were defined as above based upon the Mas 5.0 algorithm (Liu et al., 2002) (R library: affy, function: mas5calls). Raw *p*-values were adjusted for multiple hypothesis testing using the Benjamini-Hochberg method (Benjamini and Hochberg, 1995).

### 2.13 Additional datasets

Cell type-specific expression of genes was evaluated using single-nucleus RNA-sequencing data from post-mortem human lumbar spinal cord sections (GSE190442; *n* = 7 donors) (Yadav et al., 2023). Analyses were performed using QC-processed counts provided by Gene Expression Omnibus (file: GSE190442_aggregated_counts_postqc.csv.gz). Counts were obtained for 55289 cells from 64 cellular subtypes broadly classified into 11 categories (neurons, astrocytes, microglia, oligodendrocytes (OD), oligodendrocyte precursor cells (OPCs), Schwann cells, pericytes, endothelial cells, meninges, lymphocytes and ependymal cells). Raw counts were normalized to count per million mapped reads (CPM) and further analyses were performed using Log_10_(CPM +1) as an expression metric (Yadav et al., 2023).

Gene expression changes during motor neuron differentiation were evaluated using RNA-seq data from a time series in which *in vitro* monolayer human embryonic stem cells were differentiated to motor neurons (GSE140747; *n* = 45 samples) (Rayon et al., 2020). Retinoic acid and a smoothened (SMO) protein (hedgehog pathway component) agonist were used as differentiation-promoting agents. Raw sequencing reads were mapped to the UCSC GRCh38/hg38 genome using the STAR/StringTie pipeline described above (Dobin et al., 2013; Pertea et al., 2015). There was an average of 36.3 million quality-filtered reads among the 45 samples and the percentage of mapped reads, intragenic reads and exonic reads was greater than 90% for all samples (Supplementary Figures S12A–E). Cluster and principal component analyses demonstrated a strong time series effect without outliers (Supplementary Figures S12F, G). Changes in gene expression over time were evaluated using least-squares regression using voom-normalized expression data (Law et al., 2014), with FDR correction of raw *p*-values performed using the Benjamini-Hochberg approach (Benjamini and Hochberg, 1995).

## 3 Results

### 3.1 Identification of DEGs from each study separately

The analysis incorporated samples from six datasets (Table 1). Cluster analysis showed better separation of ALS and CTL samples for two datasets (GSE68605 and GSE115130) compared to the other four (Supplementary Figures S13A–F). When samples were plotted in two-dimensional principal component (PC) space, however, there was stronger separation between ALS and CTL samples, such that linear discriminant functions classified disease status with accuracy ranging from 70% (GSE76220) to 100% (GSE68605) (Supplementary Figures S13G–L). For all datasets except GSE115130, ALS and CTL samples differed significantly with respect to at least one of the top 10 PC axes (*p* < 0.05, two-sample two-tailed *t*-test; Supplementary Figures S13M–R).

Goodness-of-fit testing based on model deviance was used to evaluate the contribution of other factors to gene expression variation (e.g., age and sex). This analysis was not performed for two datasets (GSE19332 and GSE115130) since disease status was the only annotation available. For GSE56500 and GSE68605, most gene expression variation was explained by disease status, which represented the dominant factor for 49%–56% of protein-coding genes (Supplementary Figures S14C–F). For GSE18920 and GSE76220, goodness-of-fit was similarly improved by disease status, sex, age and RNA integrity number (RIN), although disease status remained the dominant factor for 28%–30% of protein-coding genes (Supplementary Figures S14A,B, G–H).

Differential expression testing generated L-shaped raw *p*-value distributions reflecting an overabundance of low *p*-values (Supplementary Figures S15A–F). Consistent with this, t-statistic Q-Q plots were non-linear to support significant expression differences between ALS and CTL samples (Supplementary Figures S15G–L). Volcano plots demonstrated balanced FC estimates between ALS-increased and -decreased genes (Supplementary Figures S15M–R) and MA plots did not show FC differences between low- and high-expressed genes (Supplementary Figures S15S–X). There were 175 and 509 DEGs identified with respect to GSE115130 and GSE56500, respectively, although fewer DEGs (≤29) were identified in other datasets at the same significance thresholds (i.e., FDR <0.10, FC > 1.50 or FC < 0.67; see Table 1).

### 3.2 Effect size comparison among studies

**FIGURE 1:**
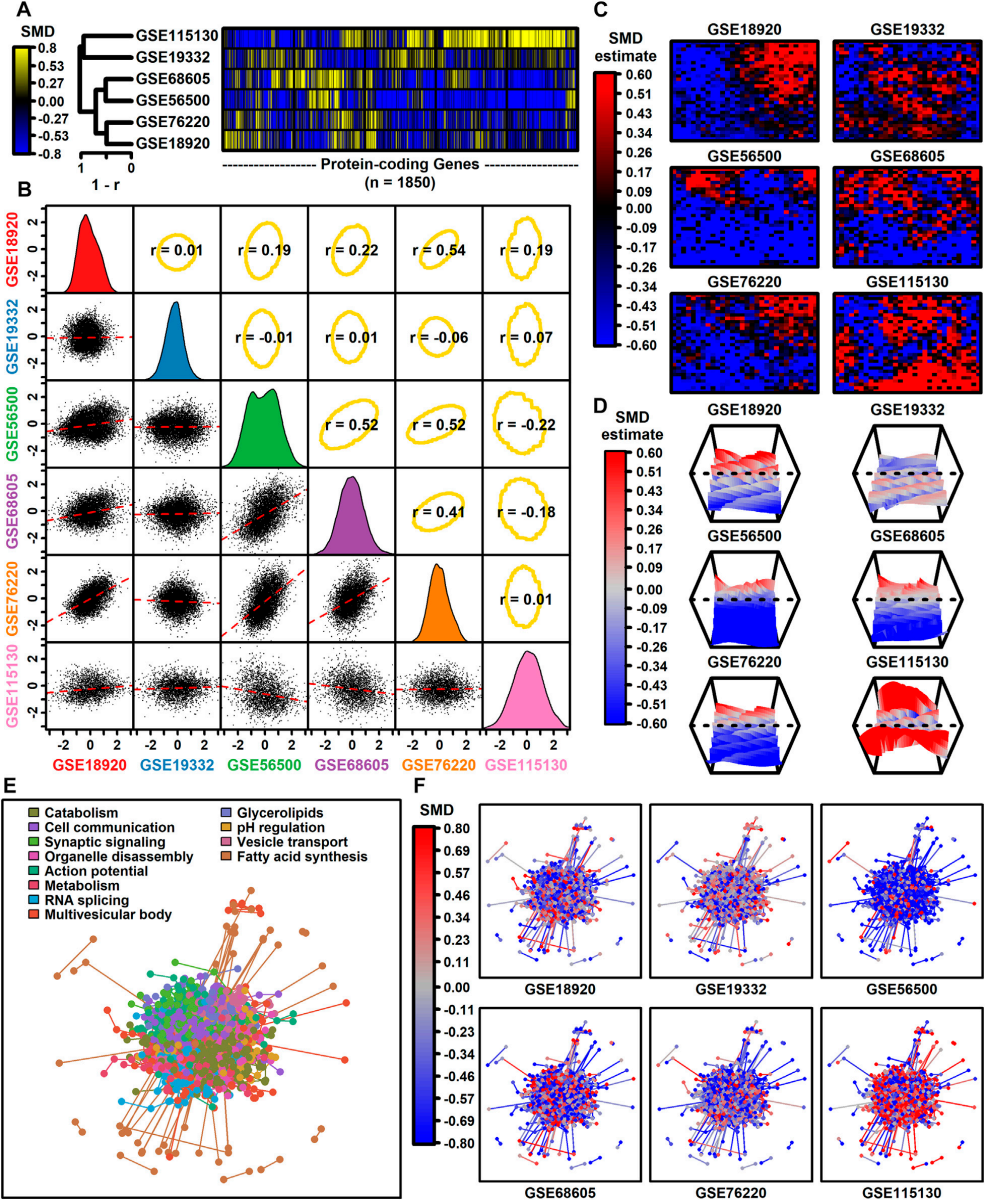
Genome-wide SMD comparison. **(A)** Cluster analysis. The heatmap shows SMD estimates for 1850 protein-coding genes (those included in all 6 differential expression analyses). Heatmap rows are clustered hierarchically using average linkage (Pearson correlation) and columns are similarly clustered (Euclidean distance). **(B)** Scatterplot comparisons. Below-diagonal boxes show scatterplots showing SMD estimates for all genes included in both differential expression analyses (red line: least squares regression estimate). Diagonal boxes show the SMD distribution for each dataset. Above-diagonal boxes show Spearman correlation estimates for each comparison (yellow ellipse: middle 90% of genes based on Mahalanobis distance). **(C)** Self-organizing maps (SOMs). An SOM map was generated based upon pooled normalized data from all 6 datasets. Genes assigned to each SOM region were color-coded based on the average SMD estimate for each SOM region. **(D)** SOM loess surface plots. Plots show the loess surface based on SMD estimates in each SOM region. **(E, F)** Protein-protein interaction network (STRING database). Network vertices correspond to one of 1343 proteins associated with mRNAs having detectable expression in all 6 meta-analysis datasets. Network edges represent 4449 high-confidence interactions between protein pairs (confidence score ≥0.700). Proteins were assigned to 12 groups based on hierarchical clustering and in part **(E)** the network is color-coded based on the GO BP term most overrepresented in each group (see legend). In part **(F)**, the same network is color-coded based on SMD estimates calculated for each dataset (see legend).

The number of protein-coding genes with detectable expression varied from 3293 to 12112 among datasets (Table 1). Among 1850 protein-coding genes with detectable expression in all six datasets, SMD estimates were positively correlated among four datasets (GSE68605, GSE56500, GSE76220, GSE189200), with estimates from these four datasets weakly correlated with those from the other two (GSE19332, GSE115130) (Figures 1A,B). SMD correlations ranged from −0.22 to 0.54 among 15 possible dataset comparisons (Figure 1B). Consistent with this, self-organizing maps color-coded by SMD estimates revealed regions with shared as well as dataset-specific patterns (Figures 1C,D). The 1850 protein-coding genes were associated with 1343 proteins having at least one high-confidence protein-protein interaction (PPI), with a total of 4449 interactions among the 1343 proteins (Figure 1E). The pattern of up- and downregulation varied across the PPI network with uniquely downregulated (GSE56500) and upregulated (GSE115130) components (Figure 1F).

### 3.3 Meta-analysis identifies DEGs with a consistent pattern across studies

DEGs with consistent SMD estimates across studies were identified using a meta-analysis model. The analysis included 9882 protein-coding genes with detectable expression in at least three of six studies. This identified 500 DEGs meeting criteria for differential expression, including 222 ALS-increased genes (FDR <0.10, SMD >0.80; Supplementary Excel file S1) and 278 ALS-decreased genes (FDR <0.10, SMD < −0.80; Supplementary Excel file S2). A subset and analysis of 342 DEGs identified at a more stringent FDR threshold (FDR <0.05) is provided as Supplementary Data (147 ALS-increased DEGs, Supplementary Excel file S3; 195 ALS-decreased DEGs, Supplementary Excel file S4).

**FIGURE 2:**
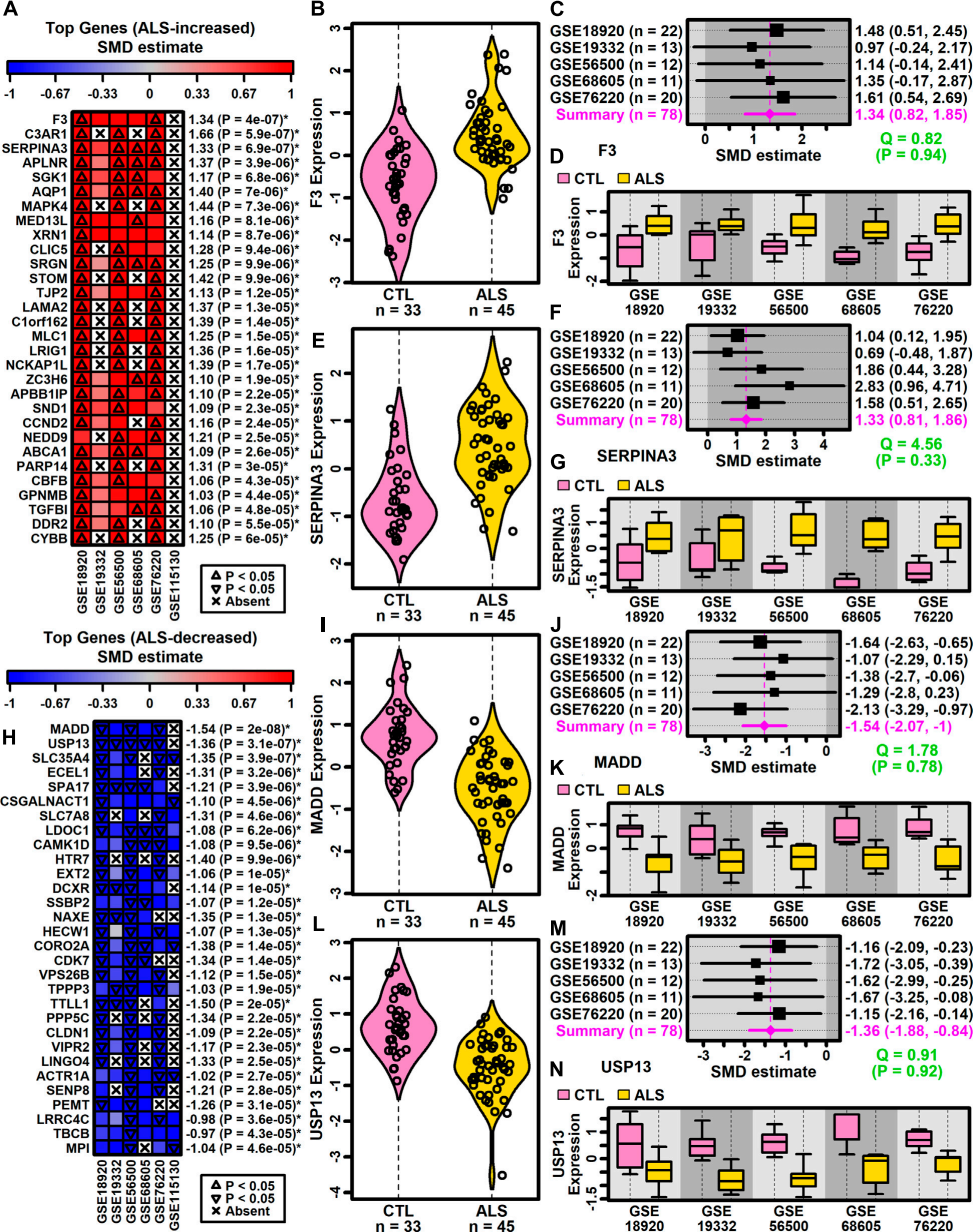
Top ranked meta-analysis genes. **(A, H)** Ranked list of **(A)** ALS-increased and **(H)** ALS-decreased genes. Heatmap colors reflect SMD estimates (see scale). The meta-SMD estimate is shown in the right margin with *p*-value (*FDR <0.10). **(B, E, I, L)** Violin plots. Log_2_-scaled expression values from each study were z-score normalized and combined. Gaussian kernal-based density estimates are plotted with expression values for each subject. **(C, F, J, M)** Forest plots. SMD point estimates are shown with 95% confidence intervals (right margin). The meta-SMD estimate is given in the bottom row (magenta font). Cochran’s Q test statistic for heterogeneity is shown with *p*-value (green font, bottom right). **(D, G, K, N)** Boxplots by study. Boxes outline the middle 50% of expression values for each group (whiskers: 10th to 90th percentiles). Log_2_-scaled expression values from each study were z-score normalized.

Genes most strongly increased in ALS included coagulation factor III tissue factor (*F3*) and serpin family A member 3 (*SERPINA3*) (Figures 2A–G). Both genes were ALS-increased in all five datasets for which expression was detectably measured (Figures 2A–G) without significant effect size heterogeneity (Figures 2C,D). The SMD for *F3* varied from 0.97 (GSE19332) to 1.61 (GSE76220) with an overall meta-estimate of 1.34 (*p* = 4 × 10^−7^; Figures 2A,C). Likewise, the SMD for *SERPINA3* varied from 0.69 (GSE19332) to 2.83 (GSE68605) with an overall meta-estimate of 1.33 (*p* = 6.9 × 10^−7^; Figures 2A,F).

Genes most strongly decreased in ALS included MAP kinase activating death domain (*MADD*) and ubiquitin specific peptidase 13 (*USP13*) (Figures 2H–N). Both genes were ALS-decreased in the five datasets for which expression was detectably measured, with no significant effect size heterogeneity (Figures 2I–N). SMD for *MADD* ranged from −1.07 (GSE19332) to −2.13 (GSE76220) with an overall meta-estimate of −1.54 (*p* = 2 × 10^−8^; Figures 2H,J). Likewise, SMD for *USP13* varied from −1.15 (GSE76220) to −1.72 (GSE19332) with an overall meta-estimate of −1.36 (*p* = 3.1 × 10^−7^; Figures 2H,M).

### 3.4 ALS-increased genes are linked to immune processes and blood vessel development with localization to plasma membrane and exosomes

**FIGURE 3:**
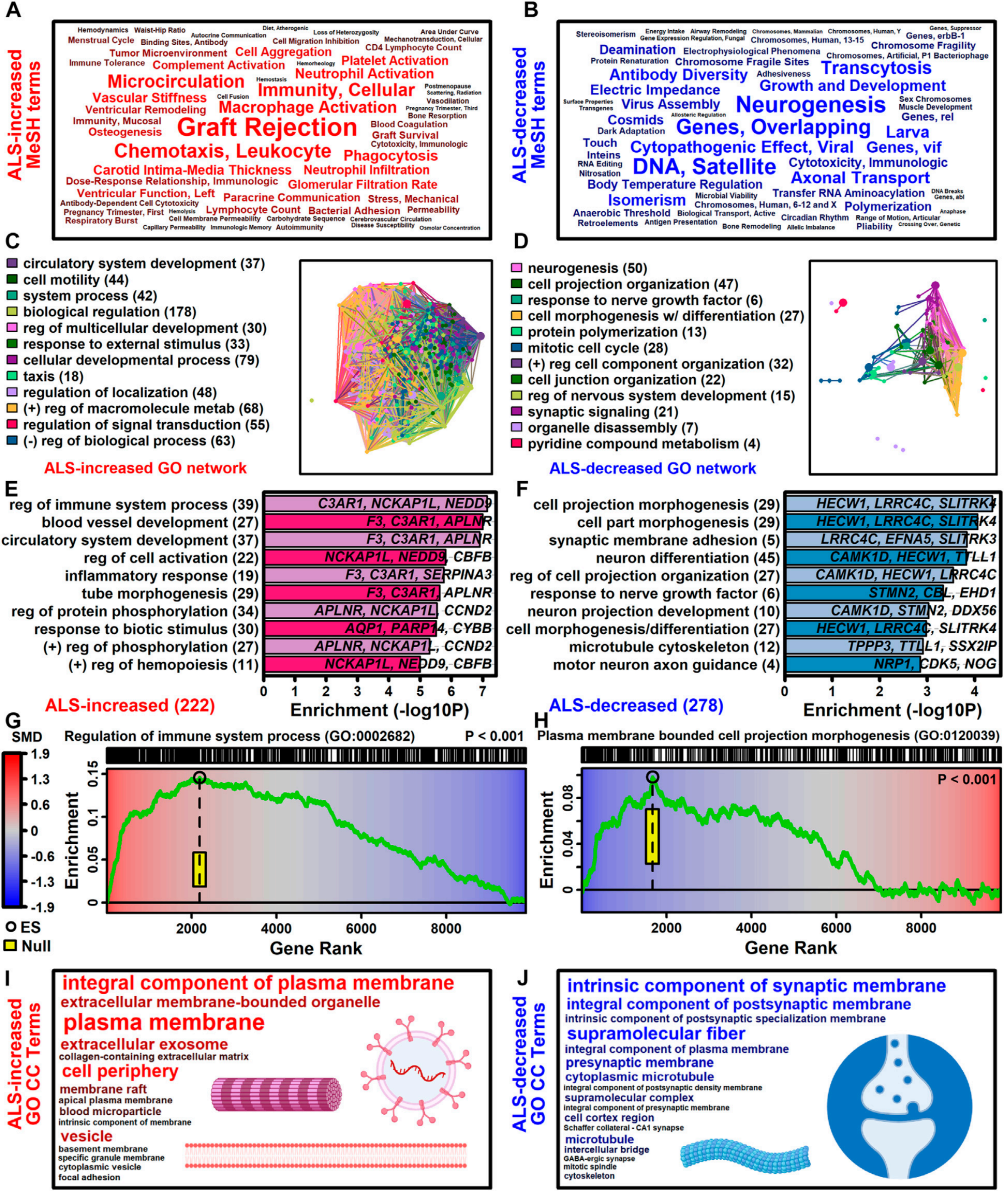
Functional enrichment analyses. **(A and B)** MeSH terms enriched among **(A)** ALS-increased and **(B)** ALS-decreased genes. MeSH term font size is inversely proportional to the enrichment *p*-value (Fisher’s Exact Test). MeSH terms were extracted from the gene2pubmed database (phenomena and processes category). **(C and D)** GO BP term network enriched among **(C)** ALS-increased and **(D)** ALS-decreased genes. Each node corresponds to a GO BP term with connections drawn between node pairs having ≥50% gene overlap. The complete set of enriched GO BP terms was clustered to form color-coded groups with the term having the largest number of genes listed as a representative. The number of GO BP terms within each group is given in parentheses. **(E and F)** Top-ranked GO BP terms. The most strongly enriched GO BP terms associated with **(E)** ALS-increased and **(F)** ALS-decreased genes are shown. Terms are ranked according to enrichment -log10-transformed *p*-values (horizontal axis). Representative genes are listed for each GO BP term and the total number of DEGs for each term is given in parentheses. **(G and H)** Gene set enrichment analysis (GSEA). Genes were ranked based upon SMD estimate (see color scale) and a running sum score was tabulated (green line) based on the position of genes linked to the indicated GO BP term (top margin). The enrichment score (black circle) is the running sum score with maximum absolute value. The yellow box outlines the middle 95% of the enrichment score null distribution from simulations in which the ranked gene list was randomly permuted (100000 trials). **(I and J)** Top ranked GO CC terms. All GO CC terms shown are significantly overrepresented among **(I)** ALS-increased DEGs (*p* < 0.001) or **(J)** ALS-decreased DEGs (*p* ≤ 0.019). The font size of each term is inversely proportional to its enrichment *p*-value.

The 222 ALS-increased DEGs were most strongly associated with MeSH terms related to immunological processes, such as graft rejection, leukocyte chemotaxis, macrophage activation, cellular immunity and phagocytosis (Figure 3A; Supplementary Excel file S1). There were 390 Gene Ontology (GO) biological process (BP) terms enriched among the 222 ALS-increased DEGs (*p* < 0.05 with at least 2 ALS-increased DEGs per GO term), which could be organized into broad categories such as development, cell motility or taxis, signal transduction and response to external stimulus (Figure 3C). Specific GO BP terms most strongly enriched among ALS-increased DEGs included regulation of immune system process, blood vessel or circulatory system development and regulation of cell activation (Figure 3E). Gene set enrichment analysis (GSEA) confirmed that genes associated with regulation of immune system process were enriched among ALS-increased DEGs (*p* < 0.001; Figure 3G). The 222 ALS-increased genes were further enriched with respect to GO cell component (CC) terms, including plasma membrane, exosome, vesicle and cell periphery (Figure 3I). Other annotations most strongly enriched with respect to ALS-increased genes included extracellular matrix structural constituent (e.g., *LAMA2*, *TGFBI*, *FN1*), TYROBP causal network in microglia (e.g., *NCKAP1L*, *APBB1IP*, *TYROBP*), and spinal cord injury (e.g., *AQP1*, *VCAN*, *AIF1*) (Supplementary Excel file S1). Drug signature analysis showed that ALS-increased DEGs were enriched with genes targeted by dexamethasone, retinoic acid and the dopamine agonist pergolide (Supplementary Excel file S1).

### 3.5 ALS-decreased genes include neurofilament light and are associated with neurogenesis, cell projection morphogenesis and motor neuron axon guidance

The 278 ALS-decreased DEGs were most frequently associated with MeSH terms related to neurogenesis, satellite DNA, viral cytopathogenic effect, transcytosis and axonal transport (Figure 3B). Overall, there were 125 GO BP terms enriched among ALS-decreased DEGs (*p* < 0.05 with at least 2 ALS-decreased DEGs per term), which could be organized into broad categories such as neurogenesis, cell projection and mitotic cell cycle (Figure 3D). Specific GO BP terms most strongly enriched among ALS-decreased genes included cell projection morphogenesis, neuron differentiation, response to nerve growth factor, and motor neuron axon guidance (Figure 3F). Neurofilament light chain (*NEFL*) was among ALS-decreased genes associated with cell projection morphogenesis and enrichment of this category among ALS-decreased genes was confirmed by GSEA (*p* < 0.001; Figure 3H). GO CC terms enriched among ALS-decreased genes included synaptic membrane intrinsic/integral component, supramolecular fiber and microtubule (Figure 3J). Other annotations enriched among ALS-decreased DEGs included Hsp90 protein binding (e.g., *PPP5C*, *STUB1*, *CDK5*) and parkin ubiquitin proteasomal system (e.g., *STUB1*, *PSMC3*, *TUBB*) (Supplementary Excel file S2). Drug signature analysis demonstrated overlap between ALS-decreased DEGs and multiple cancer drugs (e.g., ixabepilone, cabazitazel, eribulin mesylate) (Supplementary Excel file S2).

### 3.6 FUS is a top-ranked protein interaction partner of ALS-decreased DEG mRNAs

The 222 ALS-increased DEGs were enriched with genes targeted by certain lncRNAs (e.g., *lnrCXCR4*, *BALR-2*, *SBF2-AS1*, *ANCR*; FDR ≤0.005) and microRNAs (e.g., *hsa-miR-190a-3p*, *hsa-miR-223-3p*, *hsa-miR-183-3p*, *miR-29*; FDR ≤0.03) (LncRNA2Target, miRTarBase, miRDB and TargetScan databases; see Supplementary Excel file S1). Based on RNAInter database annotations (Kang et al., 2022), ALS-increased DEG mRNAs frequently interacted with RNA-binding proteins such as CELF5, CPEB1 and PABPC5 (*p* < 0.001, FDR = 1.00, Fisher’s exact test; Supplementary Figure S16A). ALS DEGs with CELF5-interacting mRNAs included *SERPINA3*, *APLNR* and *SGK1* (Supplementary Figure S16E). The percentage of ALS-increased DEG mRNAs interacting with TARDBP (TDP-43) (Suk and Rousseaux, 2020) (51.4%) did not differ compared to non-DEGs (54.8%) (*p* = 0.83, Fisher’s exact test; Supplementary Figure S16C).

The 278 ALS-decreased DEGs did not include an increased proportion of genes targeted by any specific lncRNA or microRNA (FDR ≥0.127) (Supplementary Excel file S2). Of 16177 proteins tested, ZNF326, ELAVL1 and FUS were the top 3 most overrepresented as ALS-decreased DEG mRNA interaction partners (*p* ≤ 0.0037; FDR ≥0.915; Supplementary Figure S16B). Overall, 62% of ALS-decreased DEGs had FUS-interacting mRNAs (e.g., *USP13*, *ECEL1*, *SPA17*) as compared to 54% of non-DEGs (*p* = 0.004, FDR = 0.915, Fisher’s exact test; Supplementary Figures S16B, F). The percentage of ALS-decreased DEG mRNAs interacting with TARDBP (TDP-43) (51.8%) did not differ compared to non-DEGs (54.8%) (*p* = 0.82, Fisher’s exact test; Supplementary Figure S16D).

### 3.7 DEGs have detectable expression in motor neurons from normal adult spinal cord but are not motor neuron-specific

Cell type-specific expression of genes in post-mortem spinal cords from normal adults was recently investigated using single-nucleus RNA sequencing (Yadav et al., 2023). Of 222 ALS-increased DEGs, expression of 219 were quantified by this prior study and nearly all (214 of 219) had detectable expression (i.e., CPM ≥1) in at least some motor neurons (Supplementary Excel file S1). Most ALS-increased DEGs, however, did not have motor neuron-specific expression but were expressed by multiple cell types (Supplementary Figures S17A,B). Many increased DEGs were expressed by endothelial cells, microglia and/or astrocytes (Supplementary Figure S17B), with more than half of ALS-increased DEGs having highest average expression in one of these 3 cell types (Supplementary Figure S17C). Compared to non-DEGs, increased DEGs had lower expression on average in neurons with similar or higher expression in other cell types (Supplementary Figures S17E, F). Further analysis of 64 cell subpopulations showed that ALS-increased DEGs had quantitatively lower expression in all neuronal subpopulations including motor neurons (Supplementary Figure S17E).

Of 278 ALS-increased DEGs, expression of 274 had been quantified by single-nucleus RNA sequencing (Yadav et al., 2023). Nearly all of these (271 of 274) had detectable expression in at least some motor neurons (i.e., CPM ≥1) (Supplementary Excel file S2). ALS-decreased DEGs had more robust expression in neuronal cell types (SupplementaryFigures S18A, B) and a strong majority (70.4%) were more highly expressed in neurons than any other cell type (Supplementary Figure S18C). There was no difference on average between neuronal expression of ALS-decreased DEGs and non-DEGs, although average expression of ALS-decreased DEGs was significantly lower than non-DEGs in other cell types (SupplementaryFigures S18D–F).

### 3.8 mRNAs encoding extracellular matrix proteins are disproportionately increased in ALS motor neurons

ALS-increased DEGs were enriched with respect to genes linked to the collagen-containing extracellular matrix (ECM) (Figure 3I) consistent with prior work (Rabin et al., 2010). It was therefore of interest to evaluate expression of genes related to the matrisome, defined as the set of genes encoding core ECM components and ECM-associated proteins (Hynes and Naba, 2012). Matrisome gene categories include collagens, ECM-affiliated genes, ECM glycoproteins, ECM regulator genes, proteoglycans and secreted factors (Supplementary Figure S19A). Genes associated with each category tended to have positive SMD estimates (*p* ≤ 0.033; Supplementary Figures S19B–G) with 55.2%–68.8% of genes being ALS-increased. Matrisome genes significantly elevated in ALS samples include *COL12A1* (collagen), *C1QB* (ECM-affiliated), *LAMA2* (ECM glycoprotein), *SERPINA3* (ECM-regulator), *SRGN* (proteoglycan) and *CCL2* (secreted factor) (Supplementary Figures S19H–M).

### 3.9 Motor neuron DEGs overlap significantly with genes near ALS-associated SNP loci and are mutually associated with plasma membrane, cell adhesion and semaphorin interaction

**FIGURE 4:**
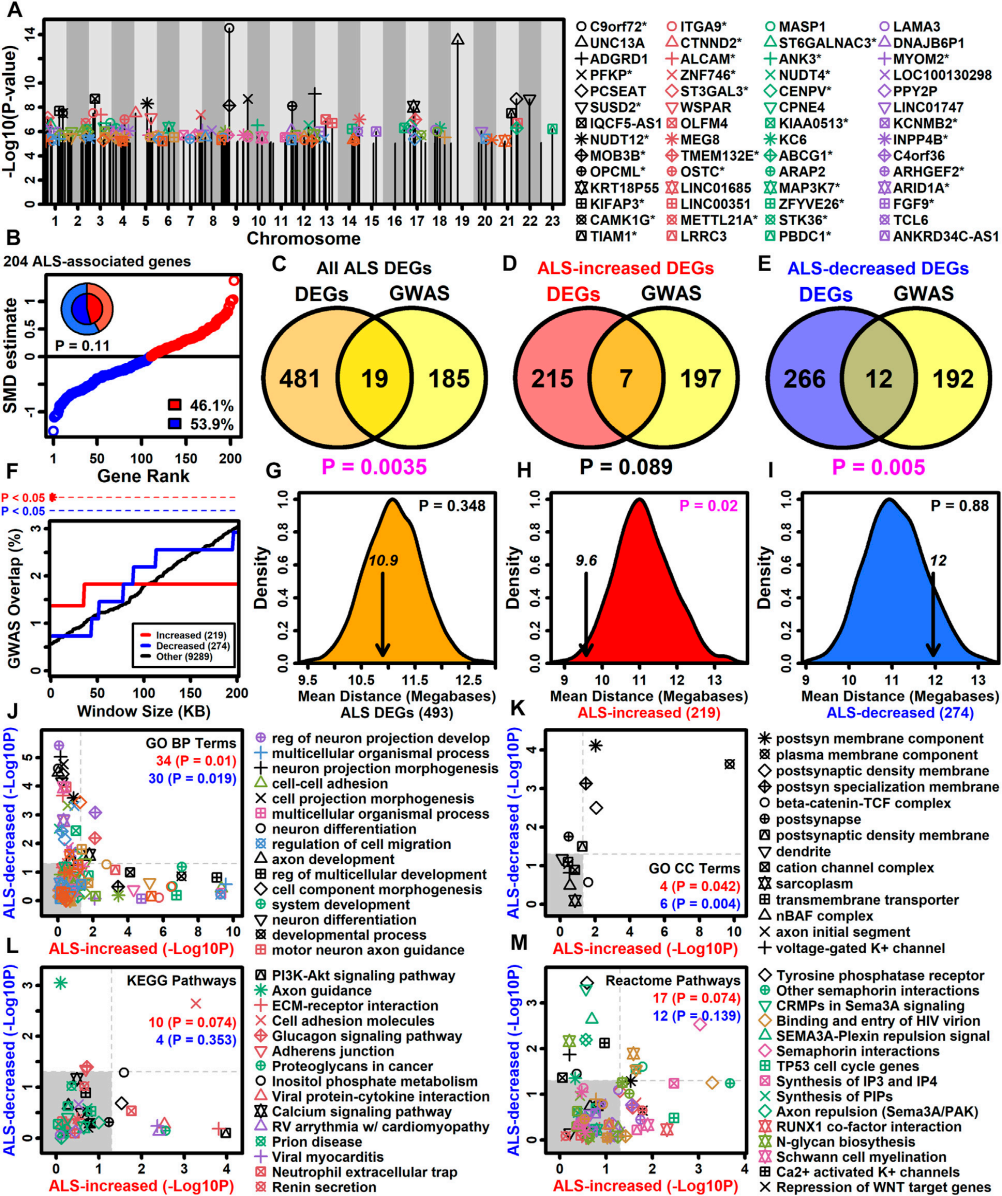
ALS GWAS genes. **(A)** Genes near ALS GWAS loci (NHGRI-EBI GWAS Catalog). Association signal *p*-values are plotted relative to chromosome location. An asterisk (*) denotes protein-coding motor-neuron expressed genes included in the meta-analysis. **(B)** SMD estimates for 204 ALS-associated protein-coding motor neuron-expressed genes. The percentage of ALS-increased and ALS-decreased genes is shown (legend) and represented by the pie chart (inner region: the 204 ALS-associated genes; outer region: all 9882 motor neuron-expressed genes). The *p*-value was obtained by testing whether the inner and outer pie chart frequencies differ significantly (Fisher’s exact test). **(C–E)** GWAS and DEG gene overlap. Overlap is shown between **(C)** all ALS DEGs and GWAS genes, **(D)** ALS-increased DEGs and GWAS genes and **(E)** ALS-decreased DEGs and GWAS genes. The significance of the overlapping gene count was evaluated using Fisher’s exact test (bottom margin *p*-value). **(F)** DEG overlap with genes located varying distances from ALS GWAS loci. The percent overlap (vertical axis) is shown for varying genome window sizes (horizontal axis) and instances of significant overlap are indicated (top margin, Fisher’s exact test). **(G–I)** Average distance of DEGs to nearest ALS GWAS locus (arrow). The distribution of average distances obtained by sampling the same number of genes at random is shown. The *p*-value (upper right) is calculated based on the position of the arrow relative to the null distribution. **(J–M)** GO BP, GO CC, KEGG and Reactome terms enriched among protein-coding motor neuron-expressed ALS GWAS genes (*p* < 0.05). The enrichment of each term was also calculated with respect to ALS-increased (horizontal axis) and ALS-decreased DEGs (vertical axis) and the number of terms significantly enriched (*p* < 0.05) with respect to each gene set is shown (upper right). *p*-values were obtained from a null distribution generated by randomly sampling the same number of genes and calculating the number of significantly enriched terms (*p* < 0.05) in each random sample.

Genes with altered expression in ALS motor neurons may be related to those linked to the disease by GWA studies (Zhang S. et al., 2022). It was possible to identify 383 protein-coding genes overlying or in linkage disequilibrum with ALS-associated SNP loci included in the NHGRI-EBI GWAS catalog (Sollis et al., 2023). Of these 383 genes, 204 had detectable expression in LCM-isolated motor neurons and had been included in the meta-analysis (Figure 4A). A slight majority of these genes were ALS-decreased (53.9%) although this trend was non-significant (*p* = 0.11, Figure 4B). The 204 genes overlapped significantly with the complete set of ALS-dysregulated DEGs (*p* = 0.0035, Figure 4C) as well as the subset of ALS-decreased DEGs (*p* = 0.005, Figure 4E), although there was no significant overlap with ALS-increased DEGs (*p* = 0.089, Figure 4D). However, ALS-increased DEGs did overlap significantly with genes close to ALS-associated SNP loci (<20 kb) (Figure 4F). Moreover, the average genomic distance between ALS-increased DEGs and their nearest ALS-associated SNP was 9.6 megabases, which was significantly less than observed in equally sized gene sets chosen randomly (*p* = 0.02, Figure 4H). However, this was not observed for ALS-decreased DEGs or the complete set of DEGs (*p* ≥ 0.348, Figures 4G,I).

Gene sets may have annotation-level correspondence even if few genes are in common (Wang et al., 2007). Annotations enriched among the 204 SNP-proximal genes were therefore assessed to determine if they are also enriched among ALS-increased and ALS-decreased DEGs (Figures 4J–M). Of 129 GO BP terms enriched among SNP-proximal genes (*p* < 0.05 with ≥2 SNP-proximal genes per GO BP term, conditional hypergeometric test), 34 and 30 were likewise enriched among ALS-increased and ALS-decreased DEGs, respectively, in each case exceeding the number of enriched terms seen in random gene sets of the same size (*p* ≤ 0.019, Figure 4J). For example, regulation of neuron projection development was highly enriched among SNP-proximal genes as well as ALS-increased DEGs, whereas cell-cell adhesion was enriched among SNP-proximal genes and ALS-decreased DEGs (Figure 4J). With respect to GO CC terms, there was a significant fraction mutually enriched between SNP-proximal genes and ALS-increased DEGs (*p* = 0.042), as well as between SNP-proximal genes and ALS-decreased DEGs (*p* = 0.004), with plasma membrane component commonly enriched in all three gene sets (Figure 4K). With regard to KEGG and Reactome pathways, there was no significant tendency for terms enriched among SNP-proximal genes to also show enrichment among ALS-increased or -decreased DEGs (*p* ≥ 0.074, Figures 4L,M), although all three gene sets were enriched with respect to cell adhesion molecules (Figure 4L) and semaphorin interactions (Figure 4M).

### 3.10 ALS-increased DEGs have increased proximity to DNA elements recognized by forkhead transcription factors and motor neuron and pancreas homeobox 1 (MNX1)

Genes may be dysregulated in ALS motor neurons due to activation or repression of transcription factors (TFs) having sequence-specific interactions with DNA elements. This was investigated using position weight matrix (PWM) models characterizing binding affinities of transcription factors compiled in the Jaspar database (Rauluseviciute et al., 2023). This allowed identification of ALS-associated PWMs (AAPs) for which matching DNA elements are in closer proximity to ALS-increased or -decreased DEGs, as compared to all other motor neuron-expressed genes included in the meta-analysis. Using a nearest neighbor statistic, 60 AAPs were identified with respect to ALS-increased DEGs (FDR <0.10, Welch’s two-sample two-tailed *t*-test), although no PWMs meeting the same significance threshold were identified with respect to ALS-decreased DEGs.

**FIGURE 5:**
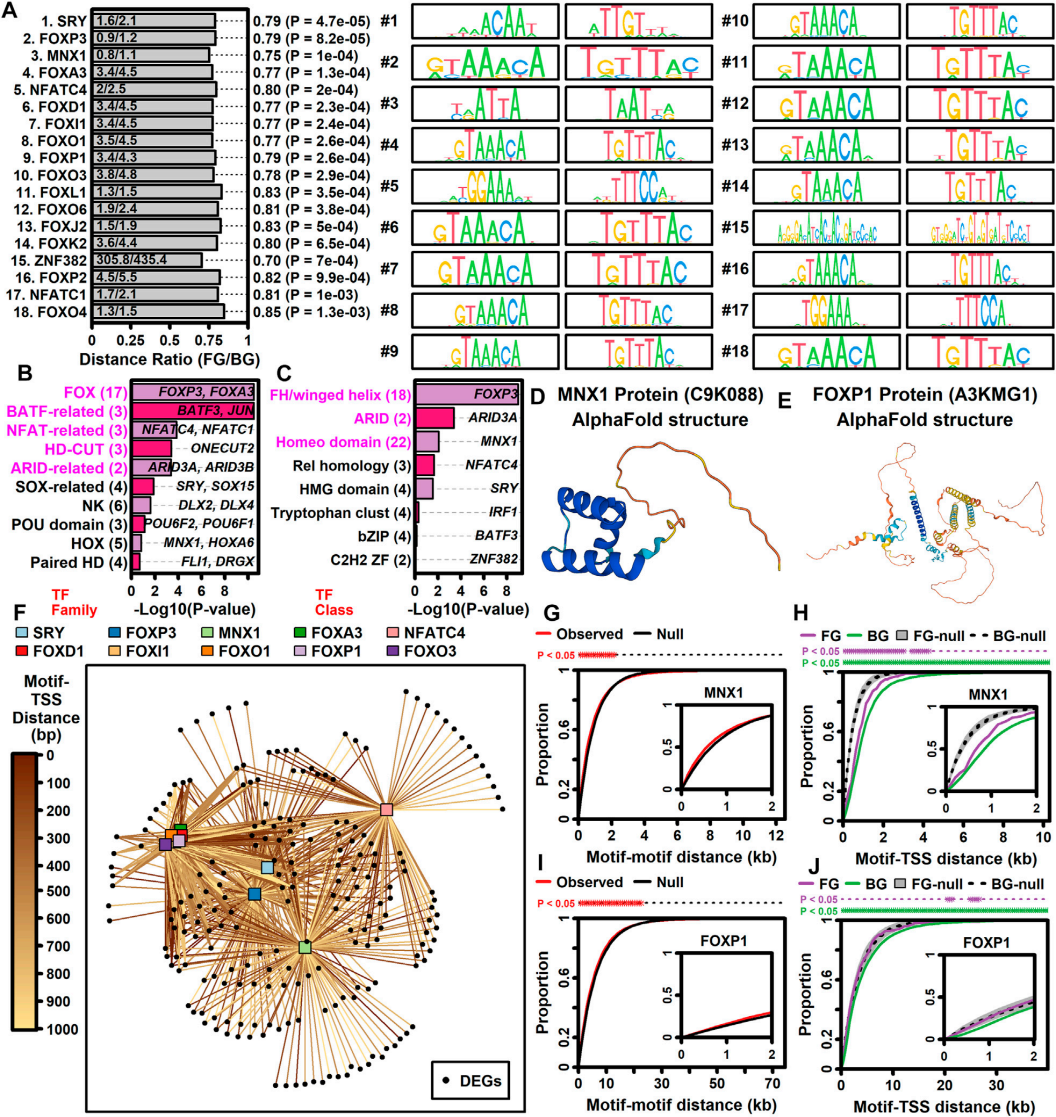
TF binding sites with increased proximity to ALS-increased DEGs. **(A)** Top 18 TF PWM models. The ratio (horizontal axis) is the average nearest neighbor distance (NN) between TF binding sites and the TSS of ALS-increased DEGs (FG, foreground) divided by the average such distance for all other motor neuron-expressed protein-coding genes (BG, background). *p*-values were obtained by comparing log10-transformed NN distances between FG and BG gene sets (two-sample two-tailed *t*-test). Sequence logos for each primary and reverse complement PWM model are shown. **(B and C)** TF families and TF classes over-represented among the 60 binding sites with increased proximity to ALS-increased genes (FDR <0.10, distance ratio <1.0). *p*-values were obtained by testing for over-representation of a given TF family or class among the 60 TF binding sites (Fisher’s exact test). The number of binding sites (out of 60) associated with each TF family or class is indicated (parentheses, left margin). Example transcription factors associated with each motif class are listed within each figure. **(D)** Motor neuron and pancreas homeobox 1 (MNX1) protein structure (alphaFold database). **(E)** Forkhead box P1 protein (FOXP1) protein structure (alphaFold database). **(F)** TF-DEG network. Connections are drawn between each TF (top margin) and ALS-increased gene (black circles) for which a putative binding site is less than 1 kb from the ALS-increased gene TSS. Darker lines are drawn in cases where the binding site is closer to the TSS location (see legend). **(G and I)** Motif-motif distance cumulative distribution functions (cdfs). Empirical cdfs are shown (red line) for the **(G)** MNX1 and **(I)** FOXP1 motifs. Black lines outline the middle 95% of null distribution cdf values obtained in simulations during which motif locations were randomly chosen throughout the genome. Asterisks (top margin) denote distances at which the observed cdf (red) is outside the 95% null region obtained by simulation (black lines). Note that null distribution regions were sufficiently narrow that black lines are not visually separable. **(H and J)** Motif-TSS distance cumulative distribution functions (cdfs) for FG and BG genes. Simulations were performed wherein motif locations were chosen at random. Grey regions outline the middle 95% null distribution for FG genes whereas dotted lines outline the middle 95% null distribution for BG genes. Top margin asterisks denote distances at which the observed cdf is outside the null region for FG and BG genes, respectively.

A PWM with consensus 5-GTA​ATT​AT/ATA​ATT​AC-3 recognized by motor neuron and pancreas homeobox 1 (MNX1) (Figure 5D) matched DNA elements with increased proximity to ALS-increased genes, with an average nearest neighbor distance of 0.80 kb between ALS-increased DEG TSSs and the predicted MNX1 binding sites, as compared to an average distance of 1.1 kb for all other motor neuron-expressed genes (Figure 5A). Such MNX1 target sites clustered together in the genome (Figure 5G) and were closer to ALS-increased DEGs than other motor neuron-expressed genes, although still further than if putative binding sites were randomized along each chromosome (Figure 5H). ALS-increased DEGs with TSS near a putative MNX1 binding site include trinucleotide repeat containing adaptor 6C (*TNRC6C*) and nuclear factor I X (*NFIX*) (Supplementary Figure S20A). Four predicted MNX1 binding sites were identified upstream of each gene (<1 kb), each of which was in a conserved region annotated as having gene regulatory activity by ORegAnno (Lesurf et al., 2016).

ALS-increased DEGs also demonstrated increased proximity to DNA elements matching PWM models sharing a 5-GTA​AAC​A/TGT​TTA​C-3 consensus sequence interacting with factors from the forkhead box (FOX) family and forkhead/winged-helix class (Figures 5B,C). These factors include FOXP3, FOXD1, FOXl1, FOXO1, FOXA3, FOXP1, and FOXO3 and were predicted to target similar genes given their DNA binding affinities (Figure 5F). For example, DNA sites matching the FOXP1 binding model (Figure 5E) had increased proximity to ALS-increased DEGs (Figure 5A). Sites matching the FOXP1 PWM had decreased distance to ALS-increased DEGs (Figure 5J) and tended to occur in clusters (Figure 5I). ALS-increased DEGs with predicted upstream FOXP1 target sites included transforming growth factor beta induced (*TGFBI*) and abhydrolase domain containing 4 N-acyl phospholipase B (*ABHD4*) (Supplementary Figure S21A), both of which had predicted FOXP1 binding sites in TSS-proximal regulatory sequences (<1 kb upstream) (Supplementary Figures S21B, C).

### 3.11 ALS-associated SNPs with genotype-dependent transcription factor binding sites

The NHGRI-EBI GWAS catalogue was used to identify 175 lead SNPs linked to ALS by GWA studies that were in linkage with a larger set of 2613 SNP loci (*r* ^2^ ≥ 0.90). These SNPs were assessed to determine if they were at locations matching PWM models. ALS-associated PWMs (AAPs) matching DNA sequences proximal to ALS-increased DEGs were more likely than other PWMs to match ALS-associated SNP loci. APPs matched 10.65 ALS-associated SNP loci on average, whereas non-APPs matched 4.28 such loci on average (Supplementary Figure S22A). This difference was again seen when only PWMs having genotype-independent matches to SNP loci were counted (Supplementary Figure S22B).

The ALS-associated SNP rs1554165 on chromosome 2 (near *DYNC1l2*) had the largest number of AAP matches, altogether matching 17 AAP models with 10 matches being genotype-dependent (Supplementary Figure S22C). Likewise, a PWM model for ARID3A, with consensus element 5-ATTAAA/TTTAAT-3, matched the largest number of DNA elements overlying ALS-associated SNPs, including 66 such SNPs with 65 of the PWM matches being genotype-dependent (Supplementary Figure S22D). Some ALS-associated SNPs predicted to interact with AAPs in a genotype-dependent fashion were within enhancers (Supplementary Figures S22E–H). For example, a chromosome 4 SNP (rs115352980) 1.1 kb from the *HADH* gene was within a putative MNX1 binding site predicted to depend upon the SNP genotype (Supplementary Figure S22F).

### 3.12 ALS-increased DEGs are enriched among genes upregulated during motor neuron differentiation

Motifs with increased proximity to ALS-increased DEGs interacted with TFs mediating motor neuron differentiation (e.g., MNX1 and FOXP1; Figure 5) (Adams et al., 2015; Garcia-Diaz et al., 2020). Meta-analysis DEGs were thus compared to genes altered *in vitro* during differentiation of monolayer human embryonic stem cells to motor neurons (GSE140747) (Rayon et al., 2020). It was possible to identify ALS-increased DEGs upregulated during motor neuron differentiation (e.g., *MLC1*, *ANGPT1*, *CP*; see Supplementary Figures S23A, B, D) as well as ALS-decreased DEGs downregulated during motor neuron differentiation (e.g., *EFNA5*, *LGI2*, *PAQR9*; Supplementary Figures S23C, E). ALS-increased DEGs were significantly enriched among genes most strongly upregulated during motor neuron differentiation (*p* = 0.01; Supplementary Figure S23F). However, ALS-decreased DEGs were not enriched among genes decreased during motor neuron differentiation (*p* = 1.00; Supplementary Figure S23G). Among ALS-increased DEGs, those with TSS nearest to a putative MNX1 binding site (e.g., *MLC1*, *ANGPT1*, *ERBB4*) tended to be more strongly upregulated during motor neuron differentiation (*r*_s_ = −0.21, *p* = 0.0055) (Supplementary Figure S23H). There was a similar although non-significant trend with regard to predicted FOXP1 binding sites (*r*_s_ = −0.10, *p* = 0.21) (Supplementary Figure S23I).

### 3.13 ALS DEGs are similarly altered in LCM-dissected motor neurons from symptomatic SOD1-G93A mice

**FIGURE 6:**
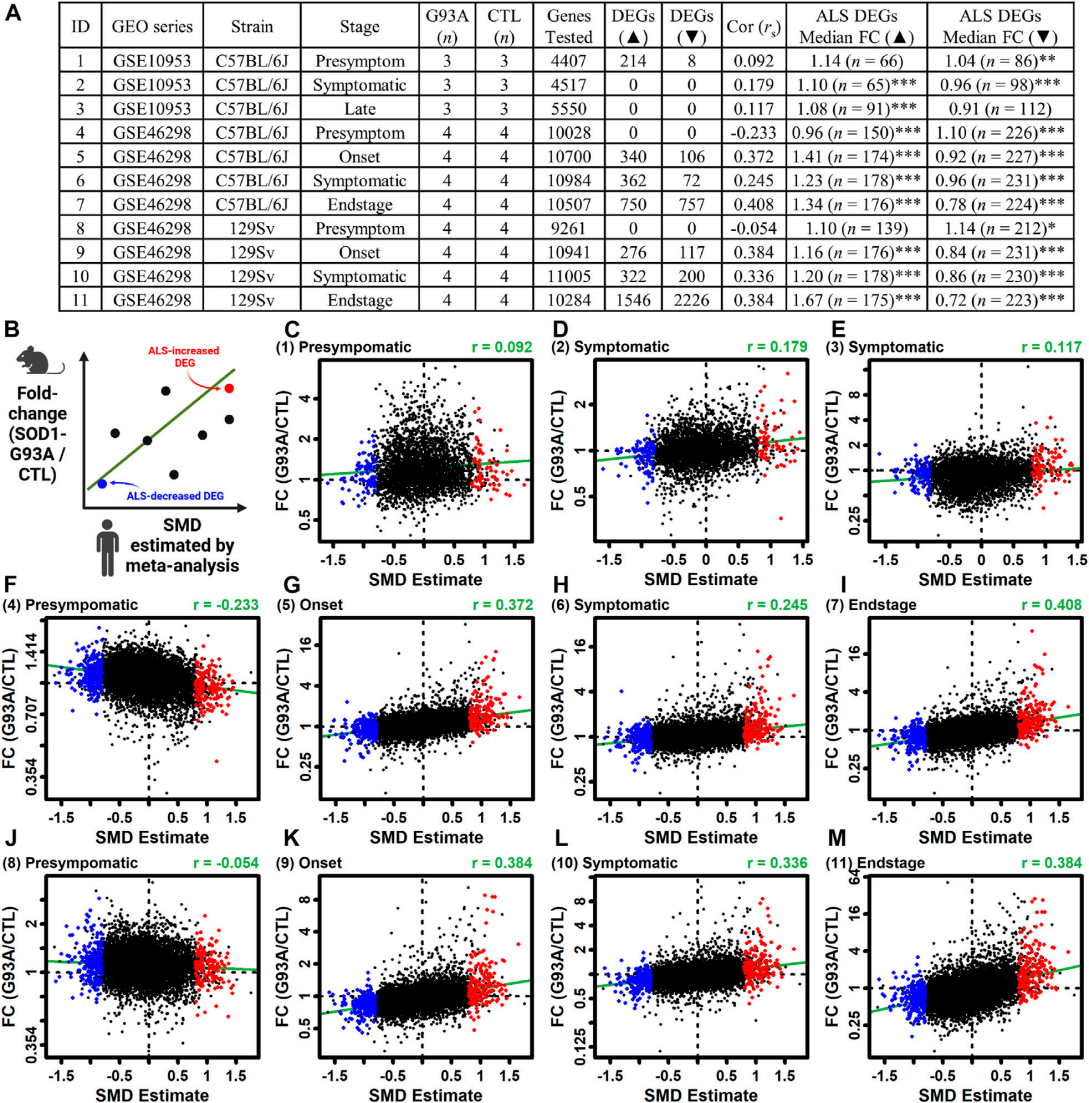
Comparison between gene expression changes in LCM-dissected ALS spinal cord motor neurons and those from the SOD1-G93A ALS mouse model. **(A)** Mouse studies. The table lists the ID number for each study, GEO series identifier, mouse strain, stage of disease onset, sample sizes, number of protein-coding genes including in the analysis, number of increased DEGs (FDR <0.10 with FC > 1.50), number of decreased DEGs (FDR <0.10 with FC < 0.67), Spearman correlation between mouse FC estimates and human SMD estimates, median FC (G93/CTL) of ALS-increased DEGs from human studies, and median FC (G93/CTL) of ALS-decreased DEGs from human studies. In the last two columns, asterisks are used to denote a significant difference between FC estimates of ALS DEGs and all motor neuron-expressed mouse genes having human orthologues (*p < 0.05, **p < 0.01, ***p < 0.001, Wilcoxon rank sum test). **(B)** Scatterplot legend. **(C–M)** Scatterplot comparisons for each mouse dataset. The mouse dataset ID is indicated (upper left) and the estimates Spearman rank correlation is shown (upper right). The green line in each figure represents the least-squares regression estimate.

The ALS meta-analysis expression signature was compared to that observed in LCM-dissected motor neurons from SOD1-G93A mice (Gurney et al., 1994). The analysis was performed using differential expression results from 11 comparisons between SOD1-G93A and CTL mice (Figures 6A, Supplementary Figure S24) (Ferraiuolo et al., 2007; Nardo et al., 2013). The genome-wide correlation between SMD and FC estimates (G93A/CTL) was near zero or even negative for presymptomatic mice (*r*_s_ ≤ 0.092) (Figures 6A,C,F,J) but was higher and always positive in symptomatic mice (*r*_s_ ≥ 0.117). Consistent with this, ALS-increased DEGs tended to be increased in G93A mice whereas ALS-decreased DEGs tended to be decreased (Figure 6). For example, for G93A mice on the 129Sv background with an endstage phenotype, ALS-increased DEGs were increased by 67% on average and ALS-decreased DEGs were decreased by 28% on average (*p* < 0.001 in each case, Wilcoxon rank sum test; Figures 6A, M). ALS-increased DEGs with increased expression in endstage G93A motor neurons from both strains included *C3AR1*, *RGS1* and *NCKAP1L* (Supplementary Figure S25A) and such genes were associated with interspecies interaction, blood vessel development and inflammatory response (Supplementary Figure S25C). Likewise, ALS-decreased DEGs with decreased expression in endstage G93A motor neurons from both strains included *PRUNE2*, *HTR7* and *USP13* (Supplementary Figure S25B) and such genes were associated with generation of neurons, neuron development and cell projection morphogenesis (Supplementary Figure S25D).

### 3.14 Genes increased (but not decreased) in LCM-dissected motor neurons from ALS patients are correspondingly altered in bulk spinal cord segments

Large-scale isolation of RNA from bulk spinal cord segments in ALS (*n* = 154) and CTL (*n* = 49) subjects has been performed (Humphrey et al., 2023). SMD estimates from this study were positively correlated with FC estimates (ALS/CTL) from each spinal cord segment (0.127 ≤ *r*_s_ ≤ 0.206) (Supplementary Figures S26A–C) as well as FC estimates averaged across segments (*r*_s_ = 0.207) (Supplementary Figure S26D).

Based on differential expression statistics reported previously (Humphrey et al., 2023), there were 62 protein-coding genes with ALS-increased expression in each cord segment (FC > 1.50 with *p* < 0.05 in each segment and FDR <0.10 in at least one segment). These genes overlapped significantly with ALS-increased DEGs from this LCM meta-analysis (Supplementary Figure S26E), with the majority (79.0%) having correspondingly positive SMD estimates (Supplementary Figure S26G). Based on GSEA, such genes were also enriched in the initial part of a gene list ranked in descending order by SMD estimate (Supplementary Figure S26I). *NCF2*, *CXCL16* and *ABCA1* are examples of DEGs increased in LCM-dissected motor neurons and each cord segment (FDR <0.10 in each comparison, Supplementary Figure S26K).

Likewise, based upon results reported previously (Humphrey et al., 2023), there were 62 protein-coding genes with ALS-decreased expression in each cord segment (FC < 0.67 with *p* < 0.05 in each segment and FDR <0.10 in at least one segment). These genes, however, did not significantly overlap with ALS-decreased DEGs identified by the LCM meta-analysis (Supplementary Figure S26F) and SMD estimates were correspondingly negative for only 48.4% of the genes (Supplementary Figure S26H). Based on GSEA, the 62 genes were not significantly enriched in the initial part of a gene list ranked in ascending order by SMD estimate (Supplementary Figure S26J). Examples of DEGs decreased in LCM-dissected motor neurons and each cord segment include *ELAVL3*, *LDOC1* and *TBCB* (FDR <0.10 in each comparison, Supplementary Figure S26L).

### 3.15 Genes decreased (but not increased) in LCM-dissected motor neurons from ALS patients have significant overlap with those altered in iPSC-derived motor neurons

**FIGURE 7:**
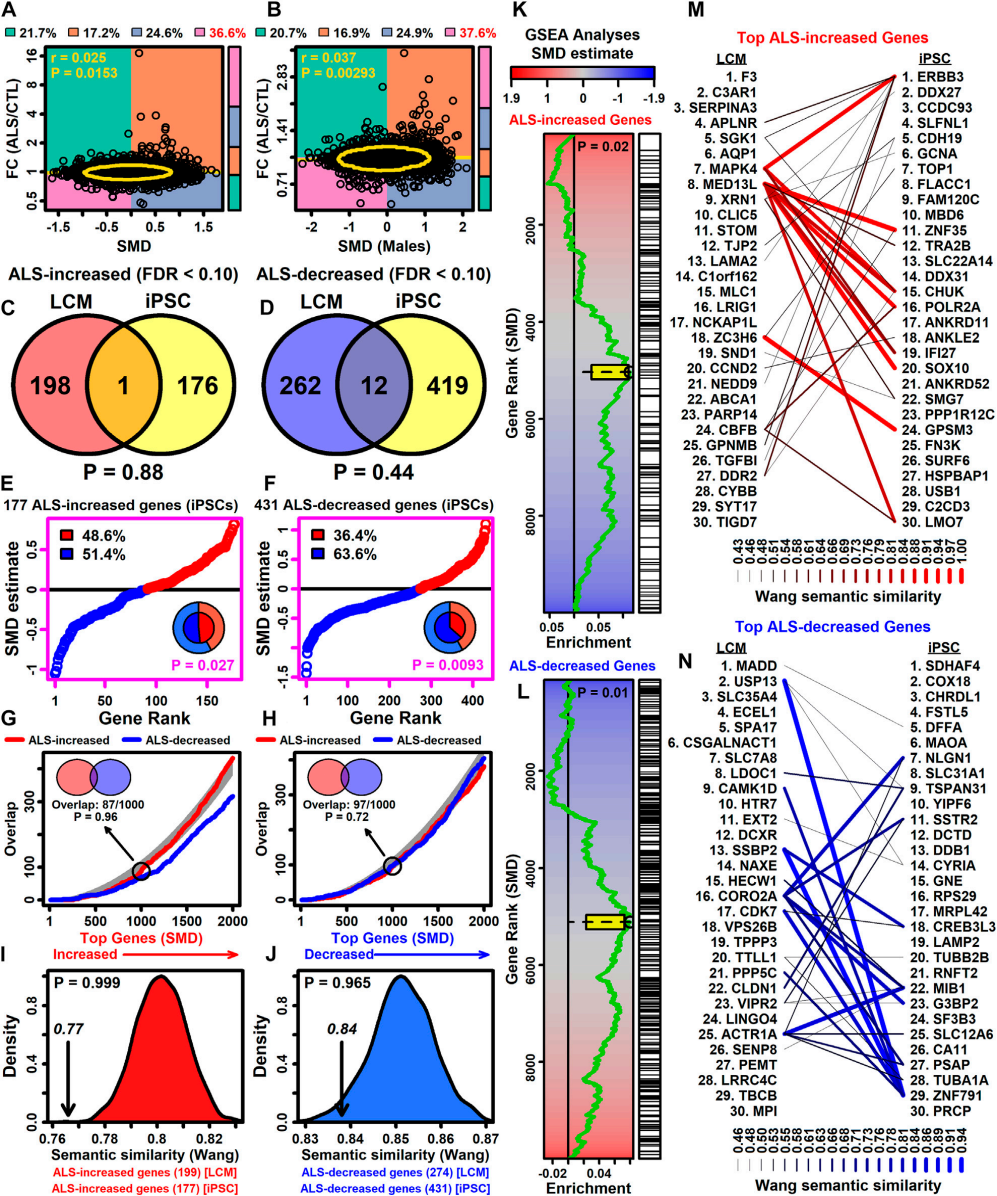
Comparison between LCM-dissected and iPSC-MN ALS signatures. The iPSC-MN-derived signature (ALS vs. CTL) was obtained from analysis of Answer ALS project samples (males only). **(A and B)** Scatterplots. Motor neuron SMD estimates are compared to iPSC-MN FC estimates and each represents an individual gene. In **(A)**, SMD estimates are calculated from both sexes, whereas in part **(B)** SMD estimates are calculated using data from males only. The proportion of genes within each quadrant is shown and Pearson’s chi-squared test was used to determine if the proportion of genes in any quadrant exceeds 25% (top margin; red font, *p* < 0.05). Quadrant proportions are graphically represented by the right sidebar. The Spearman correlation coefficient is shown with *p*-value (upper left). The yellow straight line is calculated by least-squares regression. A yellow ellipse is drawn around the middle 90% of genes closest to the bivariate mean (Mahalanobis distance). **(C and D)** Venn diagrams. In **(C)**, overlap is shown between ALS-increased genes from LCM-dissected motor neurons (SMD >0.80, FDR <0.10) and ALS-increased genes from iPSC-MNs (FDR <0.10). In **(D)**, overlap is shown between ALS-decreased genes from LCM-dissected motor neurons (SMD <0.80, FDR <0.10) and ALS-decreased genes from iPSC-MNs (FDR <0.10). The significance of overlap is evaluated using Fisher’s exact test (bottom margin *p*-values). **(E and F)** SMD estimates for genes with altered expression in iPSC-MNs. Rank-ordered symbols represent SMD estimates for each gene (red: increased in ALS LCM samples; blue: decreased in ALS LCM samples). The percentage of ALS-increased and ALS-decreased genes is shown (legend) and represented by the pie chart (inner region: the 177 or 431 genes with altered expression in iPSC-MNs; outer region: all 9882 motor neuron-expressed genes). The *p*-value was obtained by testing whether inner and outer pie chart frequencies differ significantly (Fisher’s exact test). **(G and H)** Ranked gene list overlap. In **(G)**, overlap of ALS-increased (iPSC-MN) and ALS-decreased (iPSC-MN) genes is shown (vertical axis) relative to genes most strongly increased in LCM-dissected ALS samples. In **(H)**, overlap of ALS-increased (iPSC-MN) and ALS-decreased (iPSC-MN) genes is shown (vertical axis) relative to genes most strongly decreased in LCM-dissected ALS samples. In both **(G)** and **(H)**, the dark grey region outlines the middle 95% of the null distribution (no significant overlap, *p* < 0.05). The observed overlap among the top 1000 **(G)** ALS-increased (iPSC-MN) and **(H)** ALS-decreased (iPSC-MN) genes is indicated (upper left) with associated *p*-value (Fisher’s exact test). **(I and J)** Pairwise GO semantic similarity (Wang metric). The pairwise similarity (arrow) was calculated between gene sets listed in the bottom margin. *p*-values were calculated based upon the null distribution (shown) from 1000 simulation trials. In each trial, motor neuron-expressed gene sets of the same size were generated by random sampling and the Wang similarity was calculated between the randomly generated sets. **(K, L)** Gene set enrichment analyses (GSEA). Genes were ranked based upon the SMD estimate (see color scale) and a running sum score was tabulated (green line) based on the position of **(K)** ALS-increased genes (iPSC-MN) or **(L)** ALS-decreased genes (iPSC-MN) within the ranked gene list. The enrichment score (black circle) is identified as the running sum score with maximum absolute value. The yellow box outlines the middle 95% of the enrichment score null distribution from simulations in which the ranked gene list was randomly permuted (100000 trials). (M, N) Top genes and their semantic similarity. The top 30 **(M)** ALS-increased and **(N)** ALS-decreased genes are shown. Lines between genes represent Wang semantic similarity scores between gene combinations.

Answer ALS project data has been used to identify genes differentially expressed between iPSC-MNs derived from ALS and CTL subjects (males only) (Workman et al., 2023). FC estimates (ALS/CTL) from this prior work were weakly correlated with SMD estimates from this study (*r*_s_ = 0.025, *p* = 0.241) (Figure 7A). The correlation improved slightly when SMD estimates were calculated using male samples only (*r*_s_ = 0.037, *p* = 0.003) (Figure 7B), restricting the meta-analysis to three datasets with sex annotation available (GSE18920, GSE56500, GSE76220) (Figure 7B). There was no significant overlap between DEGs or ranked gene lists from the iPSC-MN analysis and the current study (Figures 7C,D,G,H). Of 177 ALS-increased DEGs identified in iPSC-MNs (FDR <0.10), most (51.4%) were ALS-decreased in the LCM meta-analysis (SMD <0; Figure 7E), although GSEA did identify enrichment of such genes in the initial part of a gene list ranked in descending order by SMD estimate (*p* = 0.02, Figure 7M). Of 431 ALS-decreased DEGs identified in iPSC-MNs (FDR <0.10), a significant majority (63.6%) were correspondingly decreased in the LCM meta-analysis (*p* = 0.0093, Figure 7F) and GSEA demonstrated enrichment of such genes in the initial part of a gene list in ascending order by SMD estimate (*p* = 0.01, Figure 7L). There was no significant overlap between iPSC-MN and LCM DEGs based on the Wang semantic similarity of GO BP terms (Figures 7I,J) (Wang et al., 2007), although some gene pairs had functional similarity based on the Wang measure (Figures 7M,N). These analyses were repeated based upon a limited set of DEGs (9 ALS-increased, 5 ALS-decreased) reported by an independent meta-analysis of iPSC-MN datasets (ALS vs. CTL samples) (Ziff et al., 2023), but there was no significant genewise or functional overlap with meta-analysis DEGs from the current study (Supplementary Figure S27).

## 4 Discussion

**FIGURE 8:**
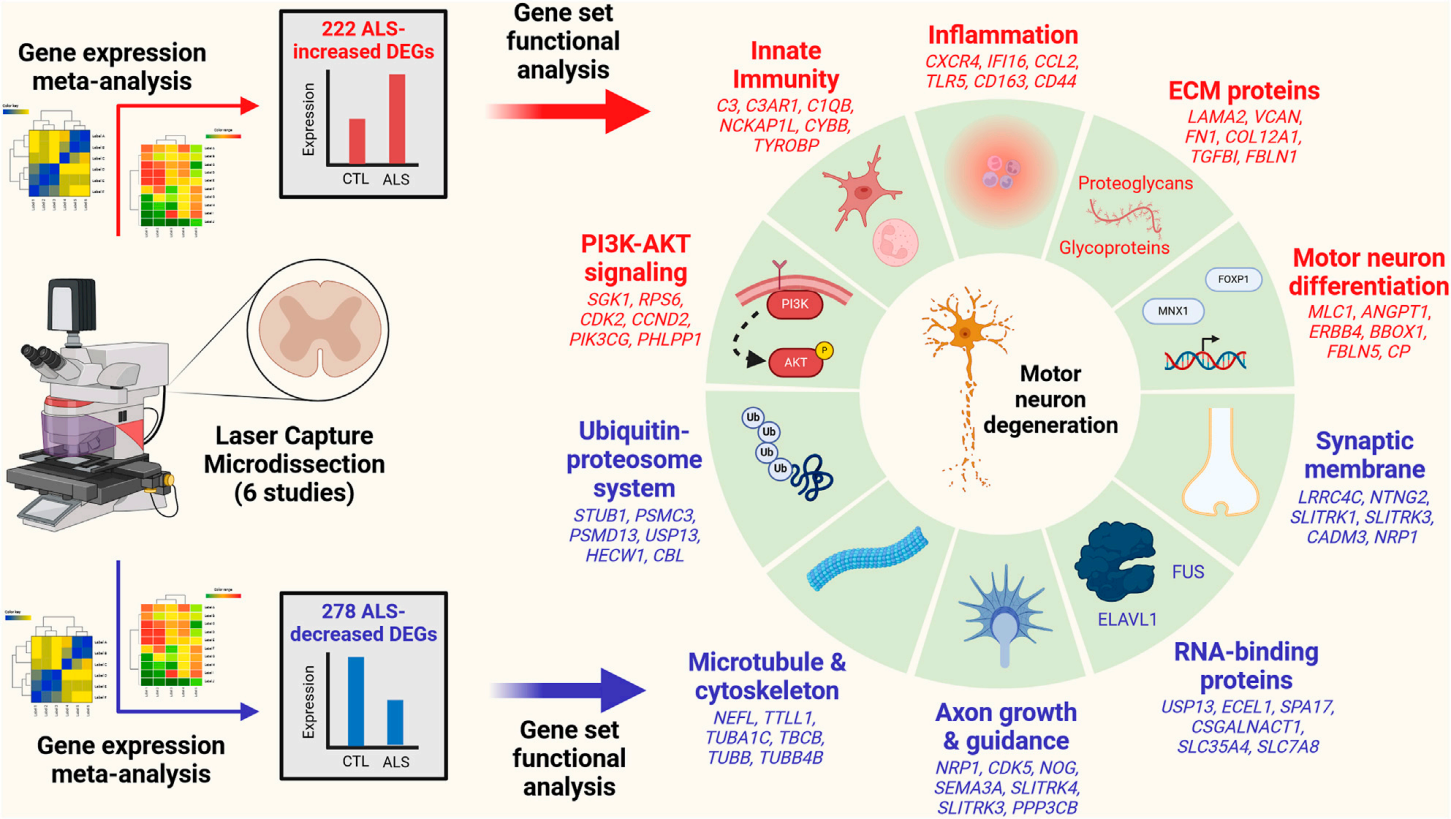
Functional categories linked to genes differentially expressed in ALS motor neurons. DEGs were identified from six gene expression studies that used LCM to isolate motor neurons from ALS and CTL post-mortem spinal cords. A meta-analysis model was applied to identify 222 ALS-increased (red) and 278 ALS-decreased DEGs (blue). Gene set analysis identified 5 core functional categories linked to ALS-increased DEGs: 1) PI3-AKT signaling, 2) innate immunity, 3) inflammation, 4) ECM proteins and 5) motor neuron differentiation. Likewise, 5 core functional categories were linked to ALS-decreased DEGs: 1) ubiquitin-proteosome system, 2) microtubule and cytoskeleton, 3) axon growth and guidance, 4) RNA-binding proteins and 5) synaptic membrane. Example DEGs associated with each category are listed.

Motor neuron degeneration is a common denominator among ALS patients despite the varying genetic or environmental factors that may underlie disease in any one individual. This study performed a meta-analysis of LCM transcriptome datasets (Cox et al., 2010; Rabin et al., 2010; Kirby et al., 2011; Highley et al., 2014; Cooper-Knock et al., 2015; Krach et al., 2018; Nizzardo et al., 2020) to identify 500 motor neuron-expressed protein-coding genes differentially expressed between ALS and CTL samples (FDR <0.10 with SMD >0.80 or SMD < −0.80). This is a larger number of DEGs than was identified from most datasets analyzed individually (Table 1). A comprehensive analysis of annotations linked to these genes highlights 10 core disease-relevant functional categories (Figure 8). The analysis also connects ALS transcriptomics to genetics, first by demonstrating overlap between DEGs and genes near ALS-associated SNP loci, and second by identifying putative DNA regulatory elements disrupted or engendered by ALS-associated SNP variants. These DNA regulatory elements interact with motor neuron-expressed transcription factors, such as MNX1 and FOXP1, which play an important role in motor neuron fate specification (Thaler et al., 1999; Adams et al., 2015). Interestingly, many DEGs identified in this study were similarly altered in LCM-dissected motor neurons from the SOD1-G93A mouse model of ALS (Gurney et al., 1994). However, most DEGs were not replicated in iPSC-MNs derived from ALS and CTL patients, suggesting that ALS motor neurons have a unique *in situ* transcriptome signature that has not been replicated by iPSC-MN studies.

Neuroinflammation is an important feature of ALS and markers of systemic inflammation have been associated with faster disease progression (Lunetta et al., 2017; Blum and Gitler, 2022). This study highlights components of innate immunity contributing to this pro-inflammatory state (e.g., neutrophils, microglia, complement) (Figure 8). The term “neutrophil degranulation” was the Reactome pathway most strongly overrepresented among ALS-increased DEGs (Supplemental Excel File S1), whereas 16.9% of ALS-increased DEGs were expressed more highly by microglia than any other spinal cord cell type (Supplementary Figure S17C). Additionally, genes linked to the alternative complement pathway were increased in ALS motor neurons, including complement C3 (*C3*) (SMD = 1.15, *p* = 0.00113, FDR = 0.036) and complement C3a receptor 1 (*C3AR1*) (SMD = 1.66, *p* = 5.9e-07, FDR = 0.00114). Furthermore, complement C1q B chain (*C1QB*), a component of the classical complement pathway, was also increased in ALS motor neurons (SMD = 1.22, *p* = 0.0039, FDR = 0.0698). An elevated neutrophil-to-monocyte ratio has been associated with disease progression in ALS patients (Murdock et al., 2016), and increased abundance of neutrophils within the spinal cord has been documented (Murdock et al., 2021). Neutrophils near motor neurons may alter the cytokine milieu in a way that favors polarization of microglia from an anti-inflammatory neuroprotective M2 (alternatively-activated) to a pro-inflammatory neurotoxic M1 (classically-activated) phenotype that can be damaging to motor neurons (Sargsyan et al., 2005; Henkel et al., 2009; Liao et al., 2012; Kim et al., 2020). This is further associated increased abundance of C3 and the receptor for its C3a cleavage product (C3AR1) near ALS motor neurons (Woodruff et al., 2008; Heurich et al., 2011; Bahia El Idrissi et al., 2016), which can indirectly enhance neutrophil recruitment via their effect on other immune cells (Daffern et al., 1995; Warwick et al., 2021). These results underscore a nexus of innate immune cells and immunomodulatory proteins in ALS, bolstering the rationale for development of drug candidates targeting innate immune pathways (Miller R. G. et al., 2022; Zhang R. et al., 2022; McGrath et al., 2023).

Spinal cord injury and neurodegeneration is associated with reactive scar formation having an inhibitory effect on axon regeneration (Fawcett et al., 2012; Burnside and Bradbury, 2014; D'Ambrosi and Apolloni, 2020). This cell non-autonomous process involves glia in combination with endothelial and pro-inflammatory immune cells, ultimately generating an organized scaffold of ECM proteins to support scar formation. Consistent with this, prior gene expression analyses of LCM-dissected motor neurons from ALS spinal cords have identified upregulation of genes encoding ECM proteins (Rabin et al., 2010), which may in some ways reflect a more widespread fibrosing process in ALS patients that extends to extra-CNS tissues (e.g., skeletal muscle, heart, skin, liver) (Apolloni and D'Ambrosi, 2022). Genes encoding ECM components play an important role in this process and can be collectively referred to as the “matrisome” with multiple protein sub-categories defined by shared signature domains (Naba et al., 2012a; Naba et al., 2012b). This study showed that genes belonging to each matrisome sub-category tend to be upregulated in ALS motor neurons, with the patten of upregulation most prominent among proteoglycans and ECM glycoproteins (Supplementary Figure S19).

Proteoglycans are heavily glycosylated protein molecules, such as chondroitin sulfate proteoglycans (CSPGs) and their receptors, which were shown to have increased abundance near reactive astrocytes in ALS rodent model spinal cords (Mizuno et al., 2008; Shijo et al., 2018). Versican (*VCAN*), for example, is a CSPG increased in ALS spinal cords (SMD = 1.05, *p* = 9.03e-05, FDR = 0.011) that contributes to ECM structure but also has pro-inflammatory activities (Wight et al., 2020). ECM glycoproteins are defined by the presence of oligosaccharide chains and several ALS-increased genes from this study encode proteins in this class (e.g., *LAMA2*, *FN1*, *SMOC1*; Supplementary Figure S19J). An interesting example is laminin subunit alpha 2 (*LAMA2*) (SMD = 1.37, *p* = 1.25e-05, FDR = 0.0043). Mutations in this gene have been associated with LAMA2-related muscular dystrophy (LAMA2-RD), which is a neuromuscular disease characterized by muscle weakness, spinal rigidity and respiratory impairment (Bouman et al., 2023), as well as by delayed nerve conduction (Shorer et al., 1995) and deceased axon myelination (Brett et al., 1998; Previtali and Zambon, 2020). These findings underscore the importance of proteoglycan and glycoproteins in scar formation during motor neuron degeneration and provide focus points for further studies to evaluate their impact on axon survival (Fawcett et al., 2012; Burnside and Bradbury, 2014; D'Ambrosi and Apolloni, 2020) and/or biomarker development (Edri-Brami et al., 2012; Xu et al., 2021).

Transcription factors coordinate gene expression to maintain homeostasis but their activation or repression can contribute to neurodegeneration (Jin et al., 2019). ALS-increased genes from this study have increased proximity to DNA motifs recognized by factors important for motor neuron fate specification, including motor neuron and pancreas homeobox 1 (MNX1/HB9) and forkhead box P1 (FOXP1). *MNX1* was originally isolated from human tonsil B lymphocytes (Harrison et al., 1994) but is widely utilized as a motor neuron-specific marker (Garcia-Diaz et al., 2020). Its expression is seen in progenitor and postmitotic motor neurons but is absent from interneurons (Thaler et al., 1999). This pattern is explained by its activity as a transcriptional repressor (William et al., 2003; Lee et al., 2008), which includes active repression of genes mediating interneuron specification (Shirasaki and Pfaff, 2002). Mice lacking *Mnx1* expression have defective motor neuron specification with axon pathfinding defects (Thaler et al., 1999) and die from respiratory failure after birth due to defective diaphragm innervation (Arber et al., 1999; Thaler et al., 2002). In this study, *MNX1* mRNA was mildly decreased in ALS motor neurons (SMD = −0.82, *p* = 0.103, FDR = 0.377), but the nearest neighbor distance between MNX1 motifs and ALS-increased genes was decreased by 25% relative to other genes (Figure 5A). Since MNX1 acts as a transcriptional repressor (William et al., 2003; Lee et al., 2008), loss of MNX1 activity in ALS motor neurons could have a transcriptional derepression effect, a common feature of genetic disease (Gabellini et al., 2003), which may account for increased expression of some DEGs in this study. In this context, it is notable that *ISL1* expression was decreased in ALS motor neurons (SMD = −0.767, *p* = 0.004, FDR = 0.069). ISL1 is a LIM homeodomain transcription factor required for motor neuron generation (Pfaff et al., 1996) that represses interneuron gene expression, similar to MNX1, although may control motor neuron differentiation through MNX1-independent pathways (Broihier and Skeath, 2002). Mice lacking *ISL1* expression also die shortly after birth due to respiratory failure and lack of diaphragm innervation (Liang et al., 2011). Future work should address how dysregulation of *MNX1*/*ISL1* expression/activity in motor neurons can impact maintenance of terminal motor neuron differentiation with potential downstream effects on cell survival and axon viability (Catela et al., 2022).

The forkhead box (FOX) transcription factor family includes diverse regulatory proteins mediating transcription of genes contributing to homeostatic and degenerative processes (Santo and Paik, 2018). ALS-increased genes from this study had increased proximity to a 5-GTA​AAC​A/TGT​TTA​C-3 motif recognized by multiple factors from this protein family (e.g., FOXP3, FOXO1, FOXP1; Figure 5A). Several such factors had detectable expression in motor neurons although none met criteria for differential expression between ALS and CTL samples. FOXO1 is a downstream effector within the PI3K-AKT signaling pathway (Brunet et al., 1999; Kops et al., 1999; Nakae et al., 1999) and genes within this pathway were enriched among ALS-increased DEGs and ALS-associated genes from GWA studies (Figure 4L). FOXO1 activity is positively regulated by cytoplasmic TDP-43 accumulation (Zhang et al., 2014), which is a hallmark feature of ALS pathology (Suk and Rousseaux, 2020). Some ALS-increased genes near FOXO motifs (e.g., *MXI1*, *BBOX1*, *ABHD4*) may therefore be upregulated secondary to FOXO activation downstream of TDP-43 mislocalization. On the other hand, these same FOXO motifs interact with FOXP1, which, similar to MNX1, is essential for motor neuron specification and target-muscle connectivity (Dasen et al., 2008; Rousso et al., 2008). FOXP1 activity is particularly critical for differentiation of lateral motor column (LMC) motor neurons at spinal cord levels corresponding to limbs (Adams et al., 2015). Findings from this analysis thus highlight forkhead family transcription factors associated with dysregulated gene expression in ALS motor neurons and it will be valuable in future work to evaluate their abundance or localization in post-mortem tissues.

Mouse models of ALS have been generated by overexpressing disease-related proteins, such as SOD1 or TDP-43, providing flexible *in vivo* systems to test mechanistic hypotheses or screen drug candidates (Philips and Rothstein, 2015). In 1993, SOD1 was discovered as the first gene linked to familial ALS, motivating development of the SOD1-G93A transgenic mouse (Gurney et al., 1994), which remains the most commonly studied disease model with many ALS-like features (Gois et al., 2020). This study showed that LCM-dissected motor neurons from SOD1-G93A mice share many transcriptomic features of post-mortem motor neurons from ALS patients. In general, the mouse-human correspondence was improved in SOD1-G93A mice having a more advanced phenotype (symptomatic or endstage) (Figure 6), with increased expression of ALS-increased DEGs associated with inflammation (*F3*, *C3AR1*, *CYBB*) and decreased expression of ALS-decreased DEGs associated with neuron generation (*CAMK1D*, *HECW1*, *SLITRK4*) (Supplementary Figure S26). On the one hand, these results are surprising, since use of the SOD1-G93A model has been critiqued as mechanistically representing only a minority of ALS patients, most of whom develop sporadic disease, with only 12% of familial cases having a causal SOD1 mutation (Renton et al., 2014). These limitations of the SOD1-G93A model may have accounted for the failure of at least some therapies with positive preclinical findings to translate successfully in clinical trials (Benatar, 2007). Since the majority of ALS patients from this meta-analysis had sporadic disease without SOD1 mutations, correspondence with the SOD1-G93A model may stem from common pathways activated or repressed during motor neuron failure. Such pathways may be linked to cellular distress signals that arise as downstream processes in diverse ALS patients, despite differences in the genetic or environmental factors that triggered disease onset (Li et al., 2013; Monahan et al., 2016; Szewczyk et al., 2023). In future work, such comparisons can be extended to other ALS mouse models, besides SOD1-G93A, which can provide an additional benchmark for model validation based on objective and quantitative criteria.

ALS-increased DEGs from this study had partial, although significant, overlap with those recently identified from a large-scale analysis of bulk spinal cords from ALS and CTL patients (Supplementary Figures S26E, G, I) (Humphrey et al., 2023). There was no significant overlap, however, with respect to ALS-decreased genes (Supplementary Figures S26F, H, J). In the bulk tissue analysis, it was proposed that increased expression of some genes was driven by a relative increase in the abundance of certain cell types, such as astrocytes, microglia, endothelial cells and/or pericytes (Humphrey et al., 2023). Since the current study included LCM studies targeting motor neuron-enriched samples, shifts in cell type abundance should contribute less to differential expression, such that most DEGs correspond to mRNAs having increased or decreased abundance in motor neurons. Nonetheless, LCM is an imperfect technique (Blum and Gitler, 2022). Isolation of motor neurons by LCM disproportionately captures the soma, excluding axonal or synaptic regions, and it is not technically feasible to fully exclude non-target cell types during tissue dissection (Kim et al., 2015). ALS-increased DEGs from this study had detectable expression in motor neurons isolated by single nucleus RNA-sequencing of the normal adult spinal cord; however, such genes were not motor neuron-specific and were also expressed by endothelial cells, microglia and astrocytes (Supplementary Figure S17). Despite advantages of LCM as a targeted approach, therefore, some changes in mRNA abundance from this study may not stem from motor neurons alone, but may also be related to shifts in cell type abundance and/or spinal cord infiltration by peripheral immune cells (Kawamata et al., 1992; Henkel et al., 2004; Zondler et al., 2016; Garofalo et al., 2020). Ultimately, immunohistochemical studies combined with high-throughput approaches such as single cell RNA-seq (Yadav et al., 2023) can be used to localize such alterations in mRNA abundance.

Few DEGs from this LCM meta-analysis were differentially expressed in prior studies of iPSC-MNs derived from ALS and CTL subjects (Workman et al., 2023; Ziff et al., 2023). There was no significant overlap between DEG sets (Figures 7C,D) and less than two-thirds of DEGs identified by LCM were altered in the same direction in iPSC-MNs (Figures 7C,D); however, significant LCM/iPSC correspondence could be demonstrated using GSEA (Figures 7K,L). We highlight three factors contributing to the limited LCM/iPSC correspondence. First, DEGs from this study were compared to those identified from an analysis of male iPSC-MN lineages, since in prior work no DEGs had been identified in female or combined sex iPSC-MN groups (Workman et al., 2023). Consistent with this, the transcriptome-wide effect size correlation did improve when LCM meta-estimates were calculated from male samples only, although the improvement was modest (*r*_s_ = 0.025 vs. *r*_s_ = 0.037; Figures 7A,B). Second, *in situ* transcriptome differences separating ALS from CTL samples may be diluted or altogether lost during the reprogramming steps required to generate iPSC-MNs, resulting in decreased signal-to-noise ratios and diminution of differential expression. Third, iPSC-MNs may be better suited as a model for embryonic-stage motor neurons, with limited ability to replicate many of the disease-related expression shifts seen in postmitotic motor neurons targeted by LCM analysis of post-mortem tissues (Ho et al., 2016). For these reasons, baseline differences between ALS and CTL iPSC-MNs may not parallel those of mature *in situ* motor neurons, although iPSC-MNs may still provide a flexible and valuable *in vitro* system for certain research objectives (Du et al., 2023).

## 5 Conclusion

This study used meta-analysis to analyze six LCM transcriptome datasets that together included 52 ALS and 37 CTL subjects, representing the largest such analysis performed to date. The analysis identified high-confidence sets of 222 ALS-increased DEGs and 278 ALS-decreased DEGs, where each DEG corresponds to a protein-coding gene having detectable expression in motor neurons. Such DEGs reflect a complex set of transcription perturbations underlying the ALS motor neuron phenotype. However, through comprehensive analysis of overrepresented gene annotations, it was possible to highlight a core set of 10 disease-relevant functional categories (Figure 8). Moreover, transcription factor regulators with a potential coordinating role were identified, including factors important for motor neuron differentiation (e.g., MNX1 and FOXP1). These factors are predicted to have sequence-specific interactions with DNA regulatory elements disrupted or engendered by ALS-associated SNP variants. Genes dysregulated in LCM-dissected motor neurons from ALS patients were often similarly altered in the SOD1-G93A mouse model but there was poor correspondence with iPSC-MNs from ALS patients. Results from this study can be further refined and updated in future work, based upon the accumulation of new data from post-mortem tissues of ALS patients. This will facilitate progress along several avenues, by helping to define a functional role for non-coding DNA segments already linked to disease status (Rich et al., 2020), by highlighting novel CSF or blood biomarker proteins (Witzel et al., 2022), or by suggesting targets that should be prioritized for antisense oligonucleotide development (Boros et al., 2022).

## Data Availability

All data analyzed in this work are available from the Gene Expression Omnibus database (GSE10953, GSE18920, GSE19332, GSE46298, GSE56500, GSE68605, GSE76220, GSE115130, GSE140747, GSE190442).
