# Supplementary material for: Meta-analysis of differential gene expression in lower motor neurons isolated by laser capture microdissection from post-mortem ALS spinal cords
Source: Front Genet. 2024 Apr 16;15:1385114. doi: 10.3389/fgene.2024.1385114 (PMC11059082; doi:10.3389/fgene.2024.1385114)
Supplement: Supplementary file 3 [file DataSheet1.pdf]

Supplementary Material

1 Supplementary Excel files

Supplemental Excel file 1. (A) 222 ALS-increased DEGs (FDR < 0.10, SMD > 0.80). Meta-analysis statistics are shown for each gene including SMD with raw and FDR-adjusted p-values. The expression of genes in motor neurons from normal adult post-mortem lumbar spinal cords is shown based on single-nucleus RNA sequencing (GSE190442). The final column lists spinal cord cell types having detectable expression of the indicated gene (mean CPM ≥ 1), where the listed cell types are ordered such that those with higher expression are listed first (GSE190442). (B) GO BP terms. (C) GO CC terms. (D) GO MF terms. (E) KEGG pathways. (F) WikiPathways. (G) Reactome pathways. (H) Disease Ontology (DO) terms. (I) Pathway commons. (J) MeSH terms. (K) DSigDB gene sets. (L) MSigDB gene sets. (M) LncRNA2Target. (N) miRTarBase. (O) miRDB. (P) TargetScan. (Q) RNAInter. Parts (B) - (L) list database annotations most strongly enriched among the 222 ALS-increased DEGs (conditional hypergeometric test or Fisher's exact test). Parts (M) – (P) list lncRNAs or microRNAs for which target genes are most strongly overrepresented among the 222 DEGs (Fisher's exact test). Part (Q) lists protein interaction partners overrepresented among the 222 DEG mRNAs (Fisher's exact test).

Supplemental Excel file 2. (A) 278 ALS-decreased DEGs (FDR < 0.10, SMD < -0.80). Meta-analysis statistics are shown for each gene including SMD with raw and FDR-adjusted p-values. The expression of genes in motor neurons from normal adult post-mortem lumbar spinal cords is shown based on single-nucleus RNA sequencing (GSE190442). The final column lists spinal cord cell types having detectable expression of the indicated gene (mean CPM ≥ 1), where the listed cell types are ordered such that those with higher expression are listed first (GSE190442). (B) GO BP terms. (C) GO CC terms. (D) GO MF terms. (E) KEGG pathways. (F) WikiPathways. (G) Reactome pathways. (H) Disease Ontology (DO) terms. (I) Pathway commons. (J) MeSH terms. (K) DSigDB gene sets. (L) MSigDB gene sets. (M) LncRNA2Target. (N) miRTarBase. (O) miRDB. (P) TargetScan. (Q) RNAInter. Parts (B) - (L) list database annotations most strongly enriched among the 278 ALS-decreased DEGs (conditional hypergeometric test or Fisher's exact test). Parts (M) – (P) list lncRNAs or microRNAs for which target genes are most strongly overrepresented among the 278 DEGs (Fisher's exact test). Part (Q) lists protein interaction partners overrepresented among the 278 DEG mRNAs (Fisher's exact test).

Supplemental Excel file 3. (A) 147 ALS-increased DEGs (FDR < 0.05, SMD > 0.80). Meta-analysis statistics are shown for each gene including SMD with raw and FDR-adjusted p-values. The expression of genes in motor neurons from normal adult post-mortem lumbar spinal cords is shown based on single-nucleus RNA sequencing (GSE190442). The final column lists spinal cord cell types having detectable expression of the indicated gene (mean CPM ≥ 1), where the listed cell types are ordered such that those with higher expression are listed first (GSE190442). (B) GO BP terms. (C) GO CC terms. (D) GO MF terms. (E) KEGG pathways. (F) WikiPathways. (G) Reactome pathways. (H) Disease Ontology (DO) terms. (I) Pathway commons. (J) MeSH terms. (K) DSigDB gene sets. (L) MSigDB gene sets. (M) LncRNA2Target. (N) miRTarBase. (O) miRDB. (P) TargetScan. (Q) RNAInter. Parts (B) - (L) list database annotations most strongly enriched among the 147 ALS-increased DEGs (conditional hypergeometric test or Fisher's exact test). Parts (M) – (P) list lncRNAs

or microRNAs for which target genes are most strongly overrepresented among the 147 DEGs (Fisher's exact test). Part (Q) lists protein interaction partners overrepresented among the 147 DEG mRNAs (Fisher's exact test).

Supplemental Excel file 4. (A) 195 ALS-decreased DEGs (FDR < 0.05, SMD < -0.80). Meta-analysis statistics are shown for each gene including SMD with raw and FDR-adjusted p-values. The expression of genes in motor neurons from normal adult post-mortem lumbar spinal cords is shown based on single-nucleus RNA sequencing (GSE190442). The final column lists spinal cord cell types having detectable expression of the indicated gene (mean CPM ≥ 1), where the listed cell types are ordered such that those with higher expression are listed first (GSE190442). (B) GO BP terms. (C) GO CC terms. (D) GO MF terms. (E) KEGG pathways. (F) WikiPathways. (G) Reactome pathways. (H) Disease Ontology (DO) terms. (I) Pathway commons. (J) MeSH terms. (K) DSigDB gene sets. (L) MSigDB gene sets. (M) LncRNA2Target. (N) miRTarBase. (O) miRDB. (P) TargetScan. (Q) RNAInter. Parts (B) - (L) list database annotations most strongly enriched among the 195 ALS-decreased DEGs (conditional hypergeometric test or Fisher's exact test). Parts (M) – (P) list lncRNAs or microRNAs for which target genes are most strongly overrepresented among the 195 DEGs (Fisher's exact test). Part (Q) lists protein interaction partners overrepresented among the 195 DEG mRNAs (Fisher's exact test).

2 Supplementary Figures

Supplemental figures S1 to S27 are shown below.

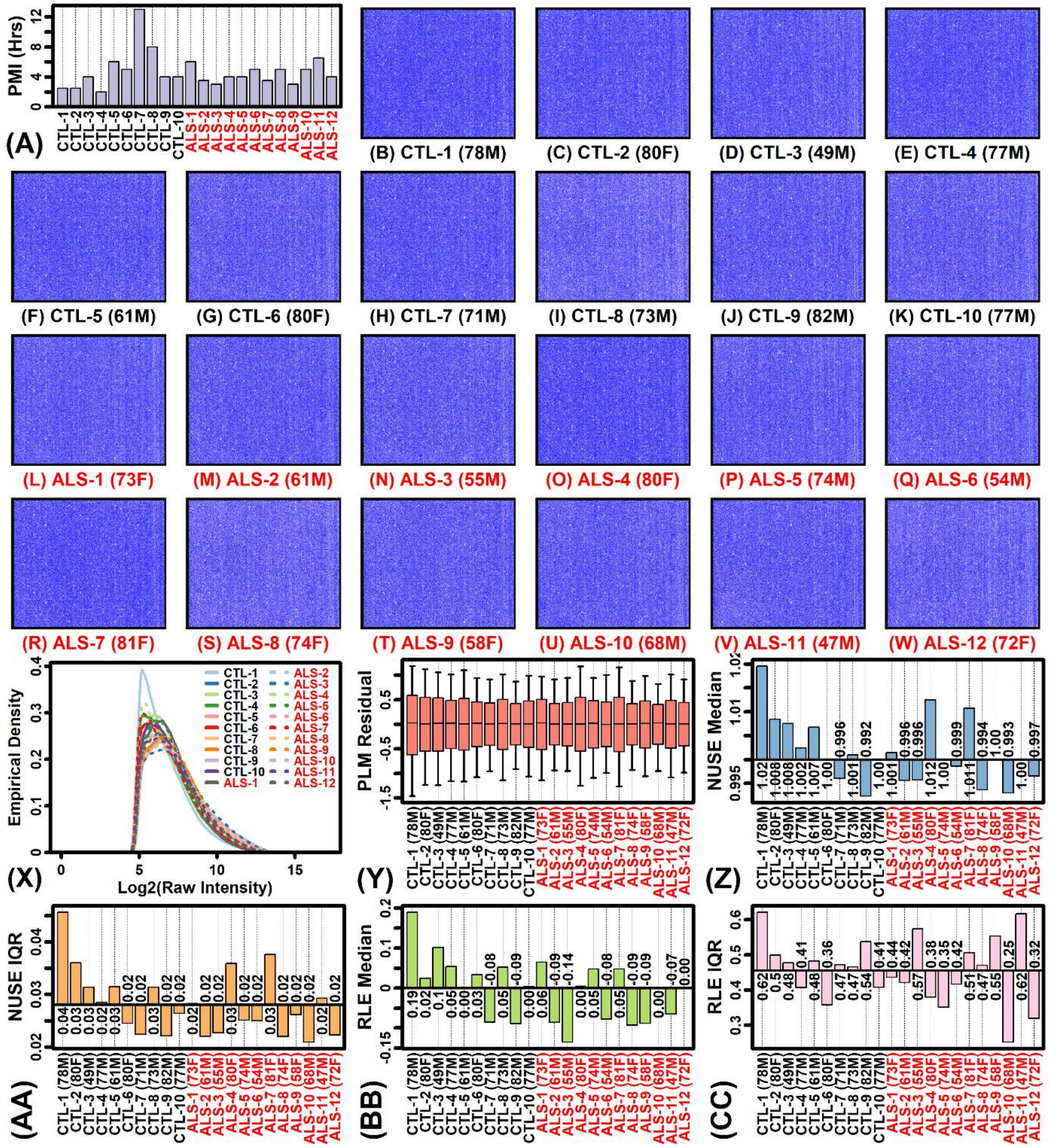

Figure S1. Microarray quality control (GSE18920). (A) Post-mortem intervals (hours, *bulbar-onset ALS). (B - W) Microarray pseudoimages. (X) Raw signal intensity distributions. (Y) Probe-level model (PLM) residuals. Boxes span residuals for the middle 50% of probes (whiskers: 10th to 90th percentiles). (Z, AA) Normalized unscaled standard error (NUSE) median and interquartile range. (BB, CC) Relative log expression (RLE) median and interquartile range.

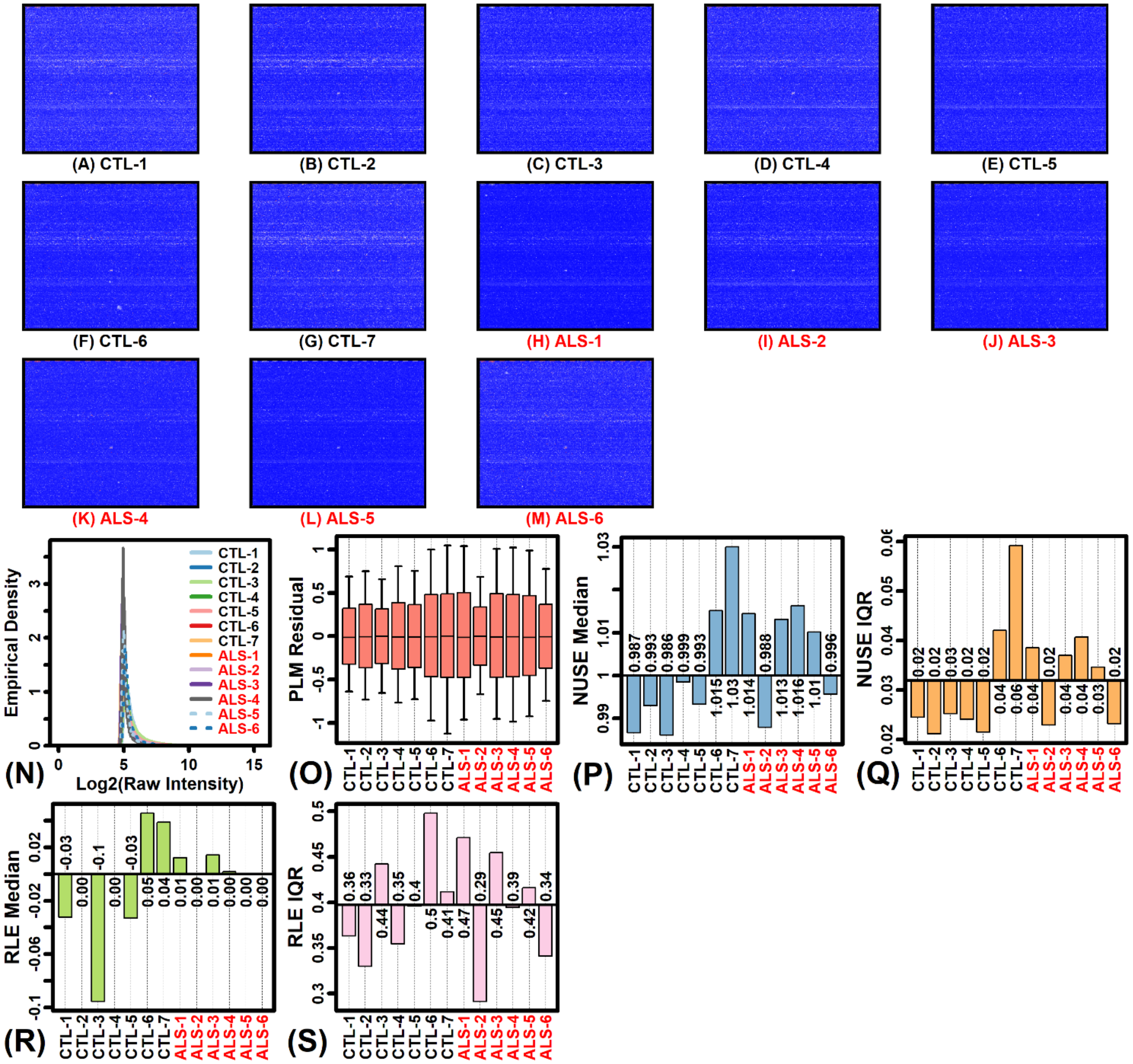

Figure S2. Microarray quality control (GSE19332). (A - M) Microarray pseudoimages. (N) Raw intensity distributions. (O) Probe level model residuals. Boxes outline the middle 50% of residual values for each array (whiskers: 10th to 90th percentiles). (P, Q) Normalized unscaled standard error (NUSE) median and interquartile range. (R, S) Relative log expression (RLE) median and interquartile range.

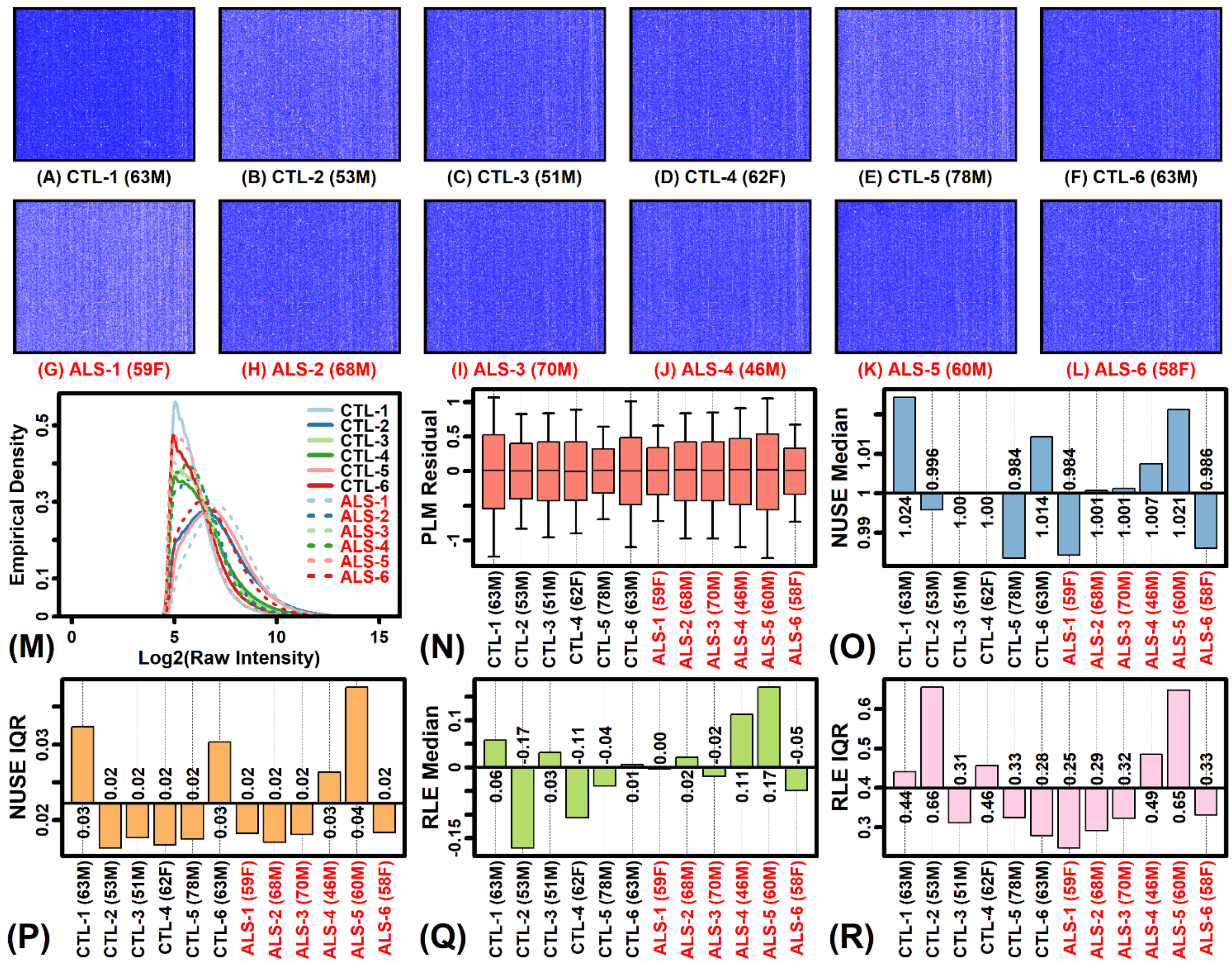

Figure S3. Microarray quality control (GSE56500). (A - L) Microarray pseudoimages. (M) Raw intensity distributions. (N) Probe level model residuals. Boxes outline the middle 50% of residual values for each array (whiskers: 10th to 90th percentiles). (O, P) Normalized unscaled standard error (NUSE) median and interquartile range. (Q, R) Relative log expression (RLE) median and interquartile range.

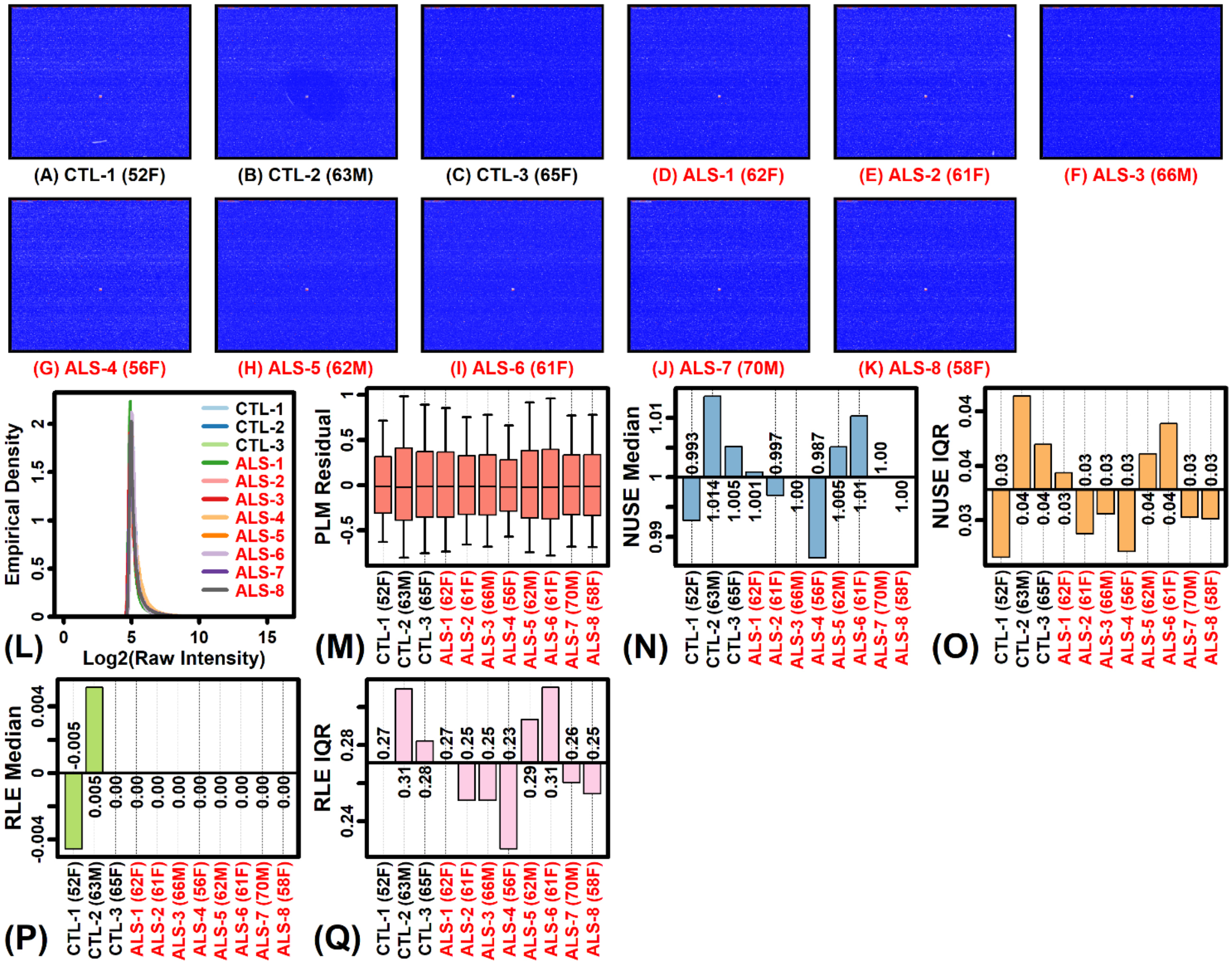

Figure S4. Microarray quality control (GSE68605). (A - K) Microarray pseudoimages. (L) Raw intensity distributions. (M) Probe level model residuals. Boxes outline the middle 50% of residual values for each array (whiskers: 10th to 90th percentiles). (N, O) Normalized unscaled standard error (NUSE) median and interquartile range. (P, Q) Relative log expression (RLE) median and interquartile range.

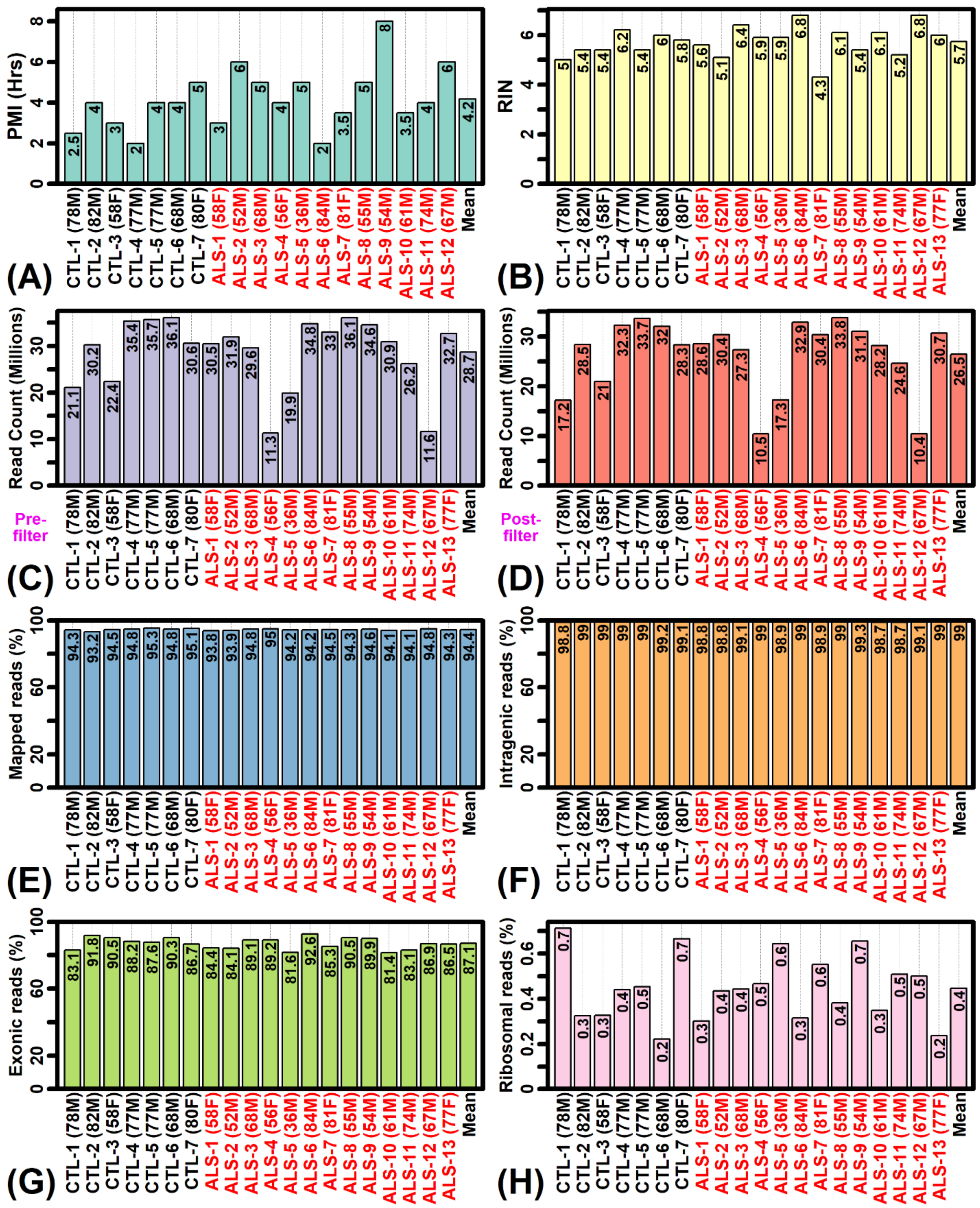

Figure S5. RNA-seq quality control (GSE76220). (A) Post-mortem interval (PMI). (B) RNA integrity number (RIN). (C) Read count (pre-filter). (D) Read count (post-filter). (E) Percentage of mapped reads. (F) Percentage of reads mapped to intragenic regions. (G) Percentage of reads mapped to exonic regions. (H) Percentage of reads mapped to ribosomal sequences.

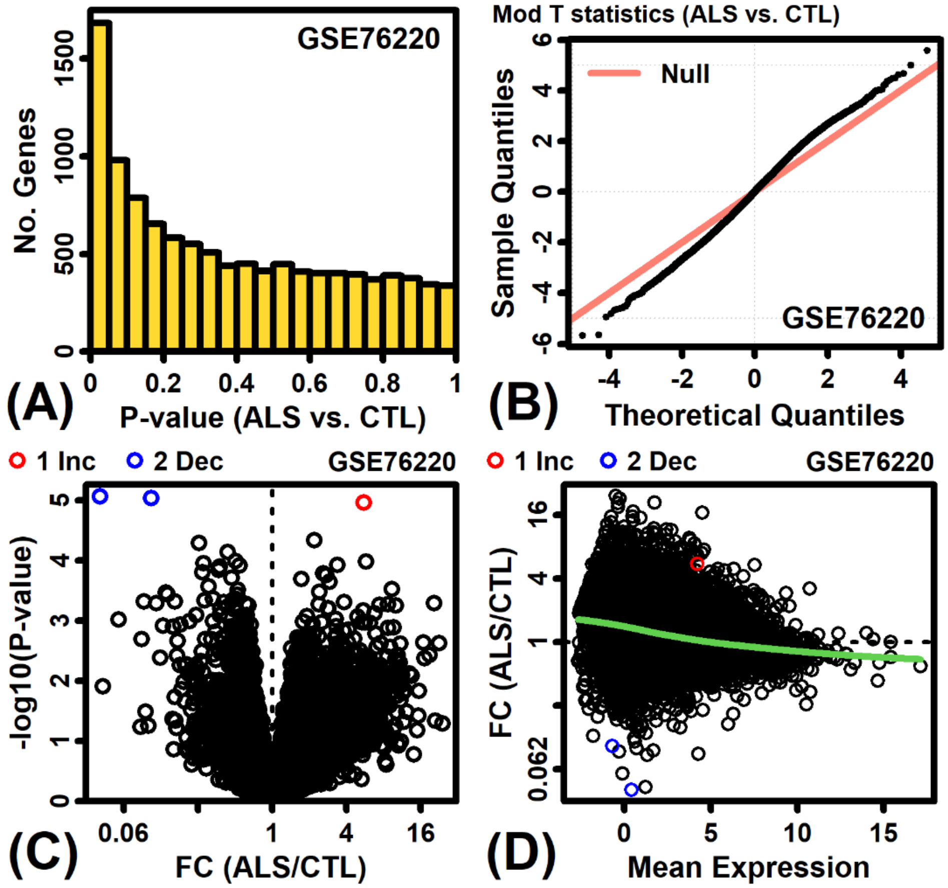

Figure S6. GSE76220 differential expression analysis (permissive gene filter). Analyses were performed using 10921 protein-coding genes with detectable expression in at least 15% of samples (i.e., ≥ 3 of 20 samples). (A) Raw p-value histogram. The distribution of raw p-values is shown among the protein-coding genes included in the differential expression analysis. (B) Q-Q plot. Observed t statistic sample quantiles (vertical axis) are plotted against theoretical quantiles of Student's t distribution (horizontal axis). A larger proportion of differentially expressed genes is suggested by deviation from a straight line. (C) Volcano plot. The -log10-transformed p-values (vertical axis) are plotted against FC estimates (horizontal axis). (D) MA plot. FC estimates (vertical axis) are plotted against average normalized expression of genes (vertical axis). The green line represents the nonparametric locally weighted smoothing estimate (loess fit). In (C) and (D), each point represents an individual gene and colors denote ALS-increased (FDR < 0.10, FC > 1.50) and ALS-decreased (FDR < 0.10, FC < 0.67) genes. The number of differentially expressed genes is indicated (top margin).

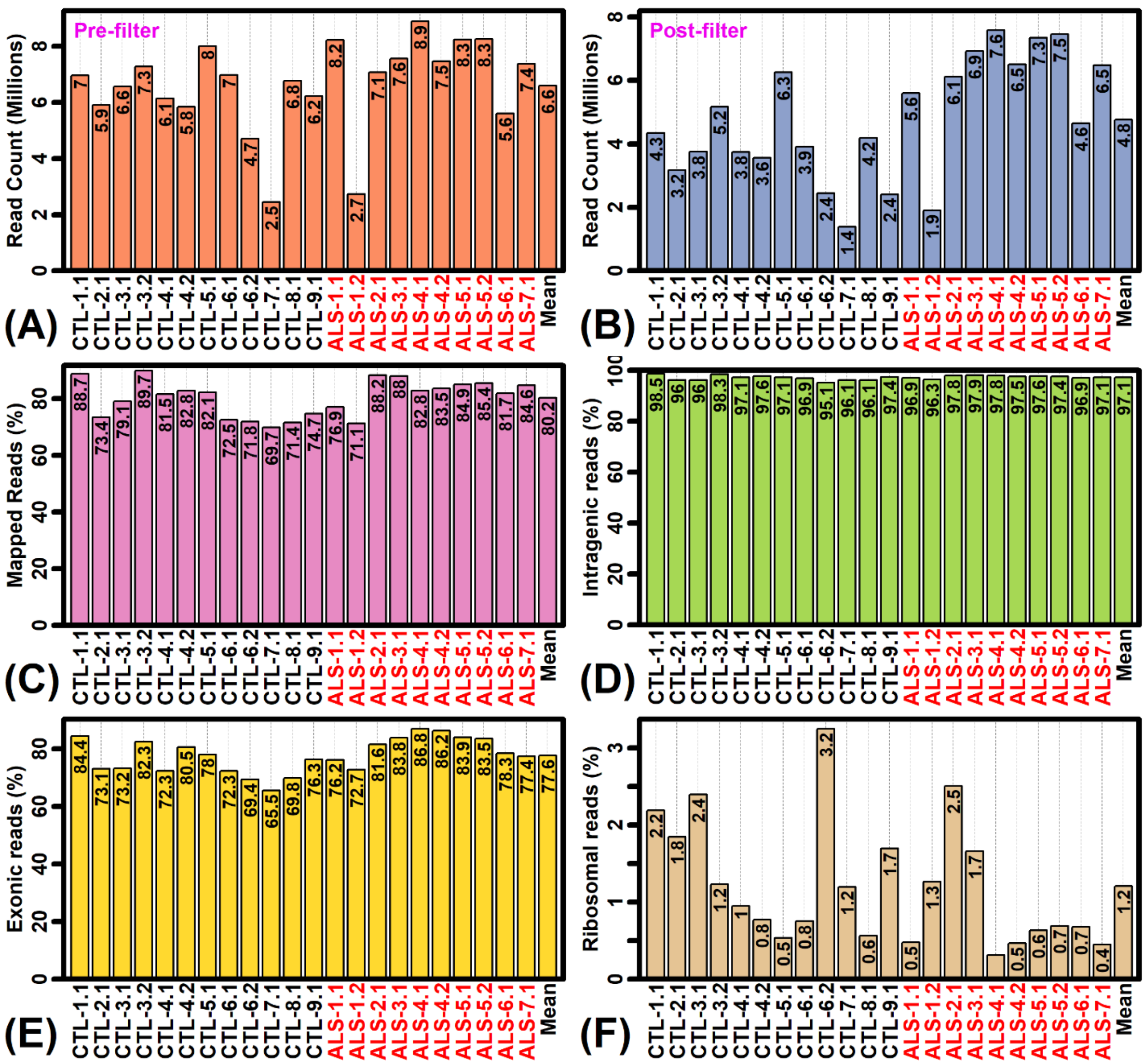

Figure S7. RNA-seq quality control (GSE115130). (A) Read count (pre-filter). (B) Read count (post-filter). (C) Percentage of mapped reads. (D) Percentage of reads mapped to intragenic regions. (E) Percentage of reads mapped to exonic regions. (F) Percentage of reads mapped to ribosomal sequences.

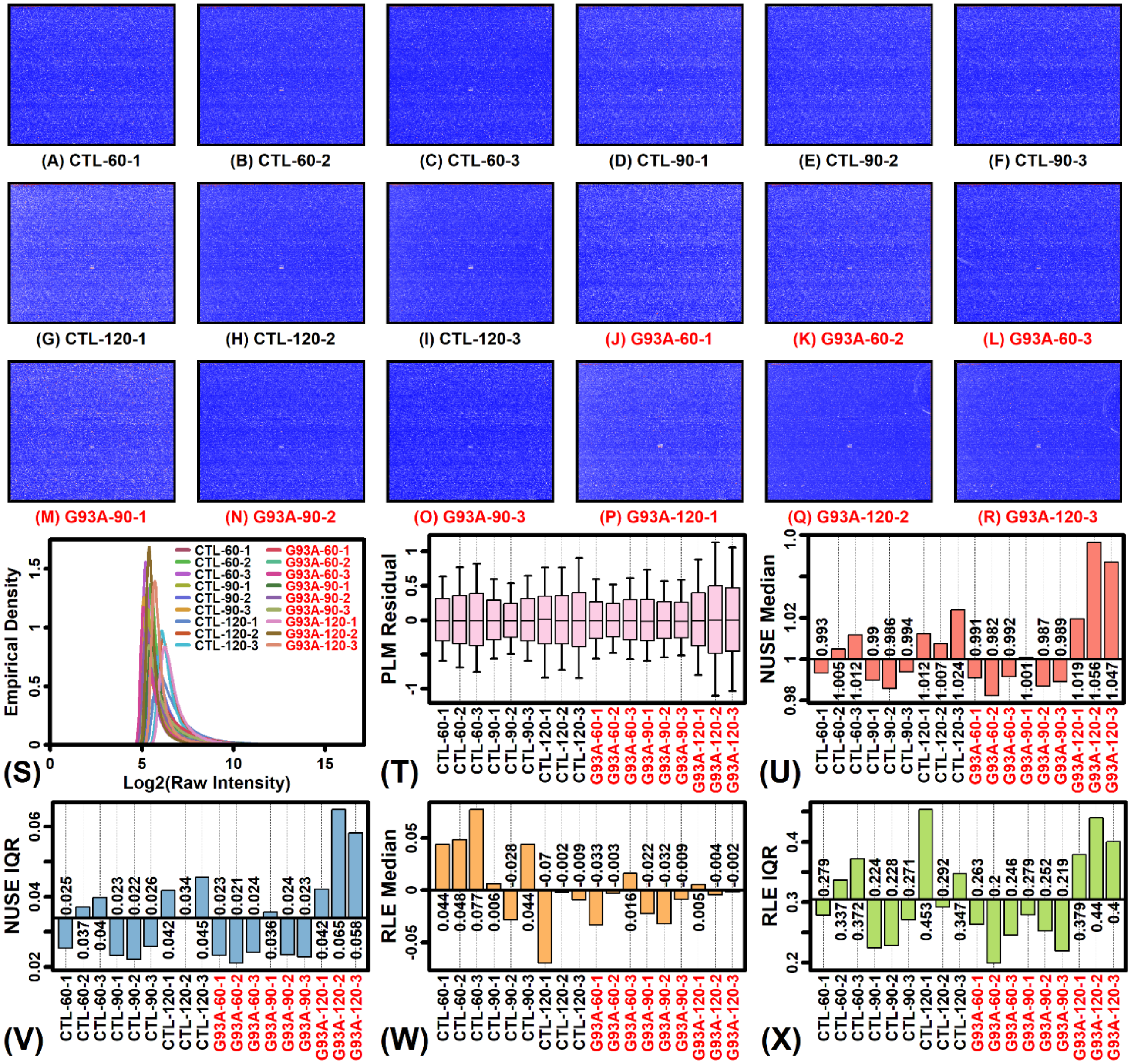

Figure S8. Microarray quality control (GSE10953). (A - R) Microarray pseudoimages. (S) Raw signal intensity distributions. (T) Probe-level model (PLM) residuals. Boxes span residuals for the middle 50% of probes (whiskers: 10th to 90th percentiles). (U, V) Normalized unscaled standard error (NUSE) median and interquartile range. (W, X) Relative log expression (RLE) median and interquartile range.

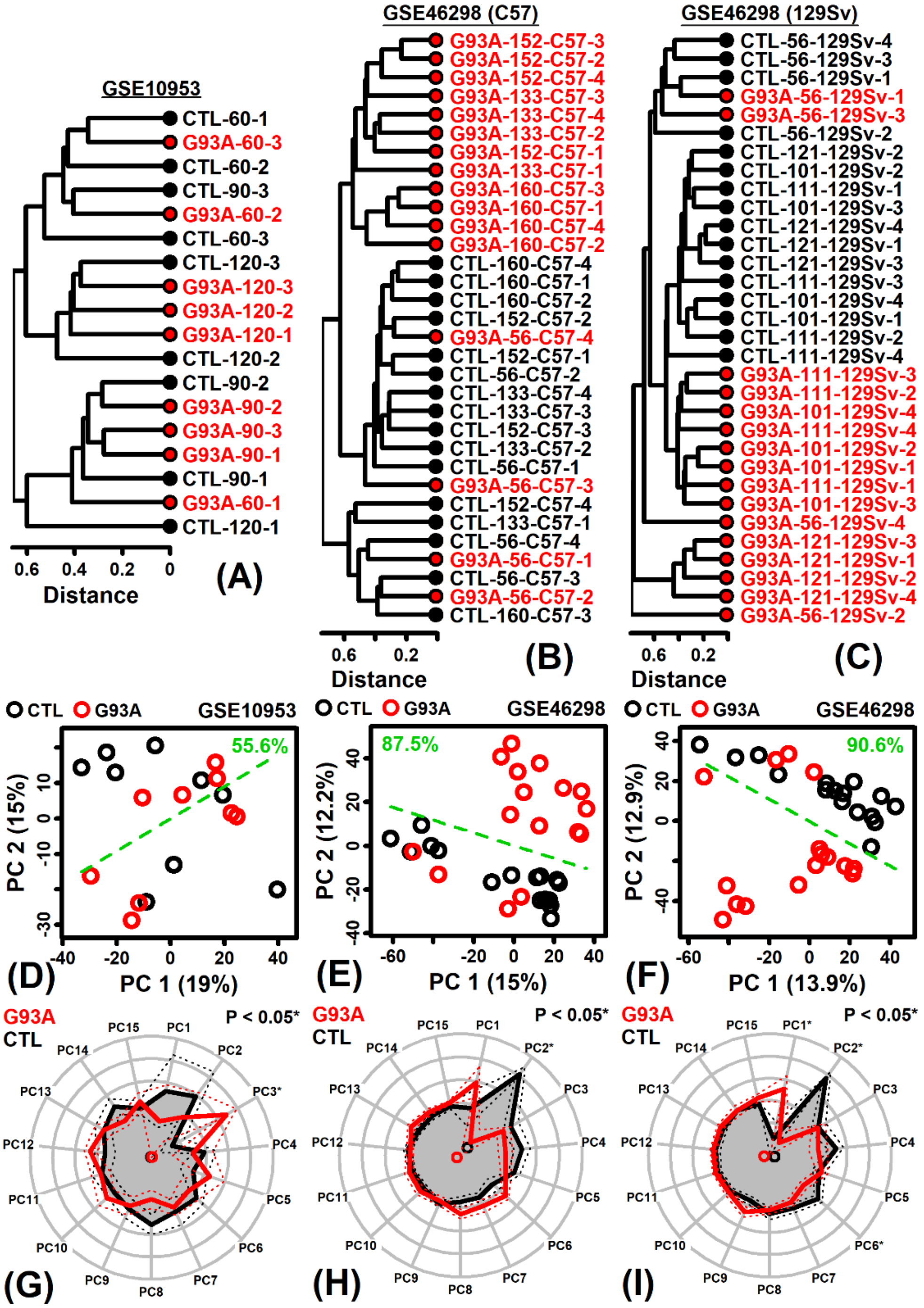

Figure S9. Cluster and principal component analyses (GSE10953 and GSE46298). (A - C) Hierarchical cluster analyses. Samples were clustered based upon Euclidean distance with average linkage. (D - F) Principal component scatterplots. Samples are plotted with respect to the first two PC axes. The dotted green line represents the linear discriminant function separating SOD1-G93A from CTL samples. The percentage of samples accurately classified (SOD1-G93A vs. CTL) using the discriminant function is shown (green font). (M - R) Principal component radial plots. Average PC values for SOD1-G93A (red) and CTL (black) samples are plotted (thick lines) ± 1 standard error (dotted lines). PC axes with significant differences between SOD1-G93A and CTL sample scores are indicated (*, $P < 0.05$, two-sample two-tailed t-test).

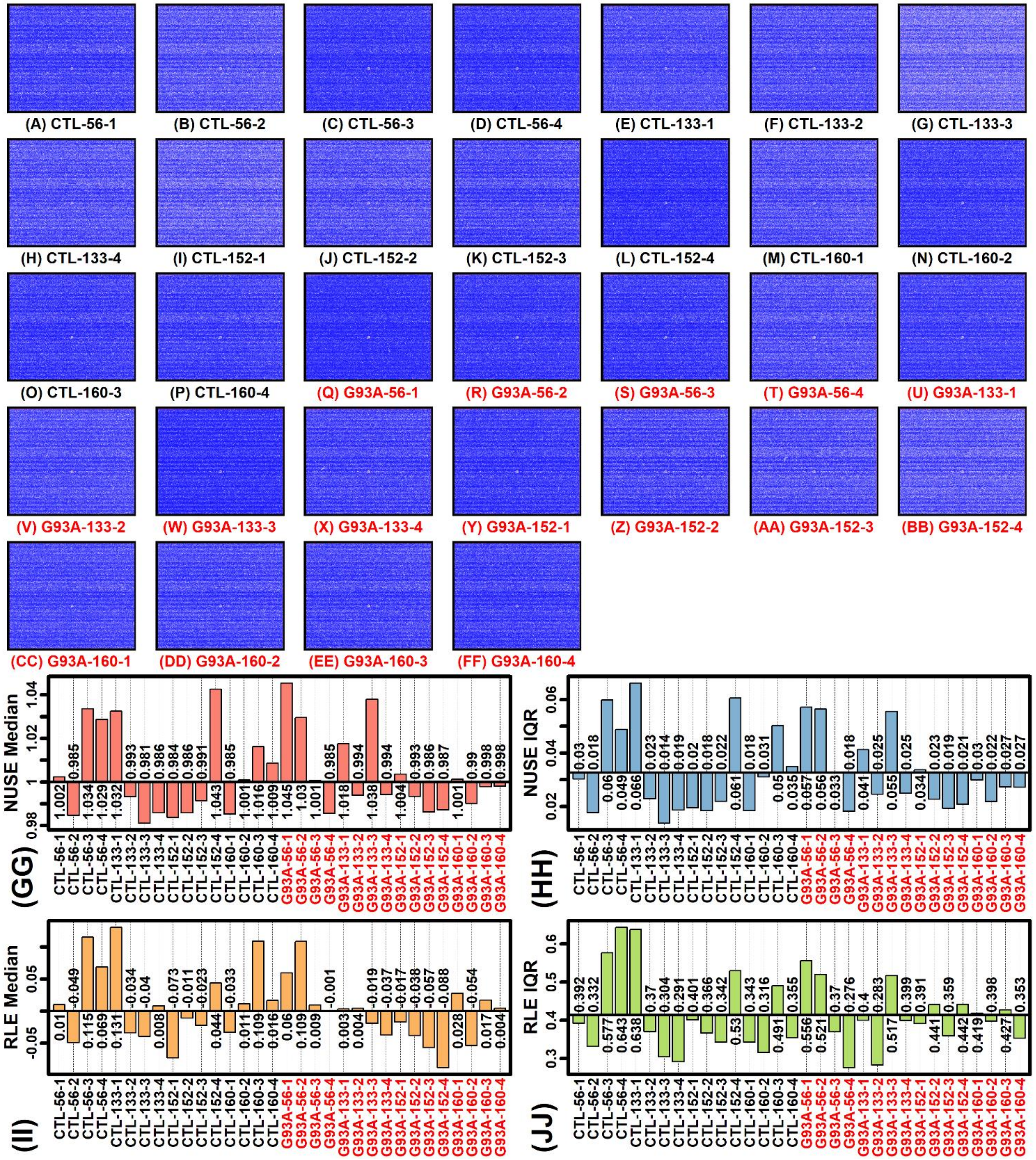

Figure S10. Microarray quality control (GSE46298, C57BL/6J). (A - FF) Microarray pseudoimages. (GG, HH) Normalized unscaled standard error (NUSE) median and interquartile range. (II, JJ) Relative log expression (RLE) median and interquartile range.

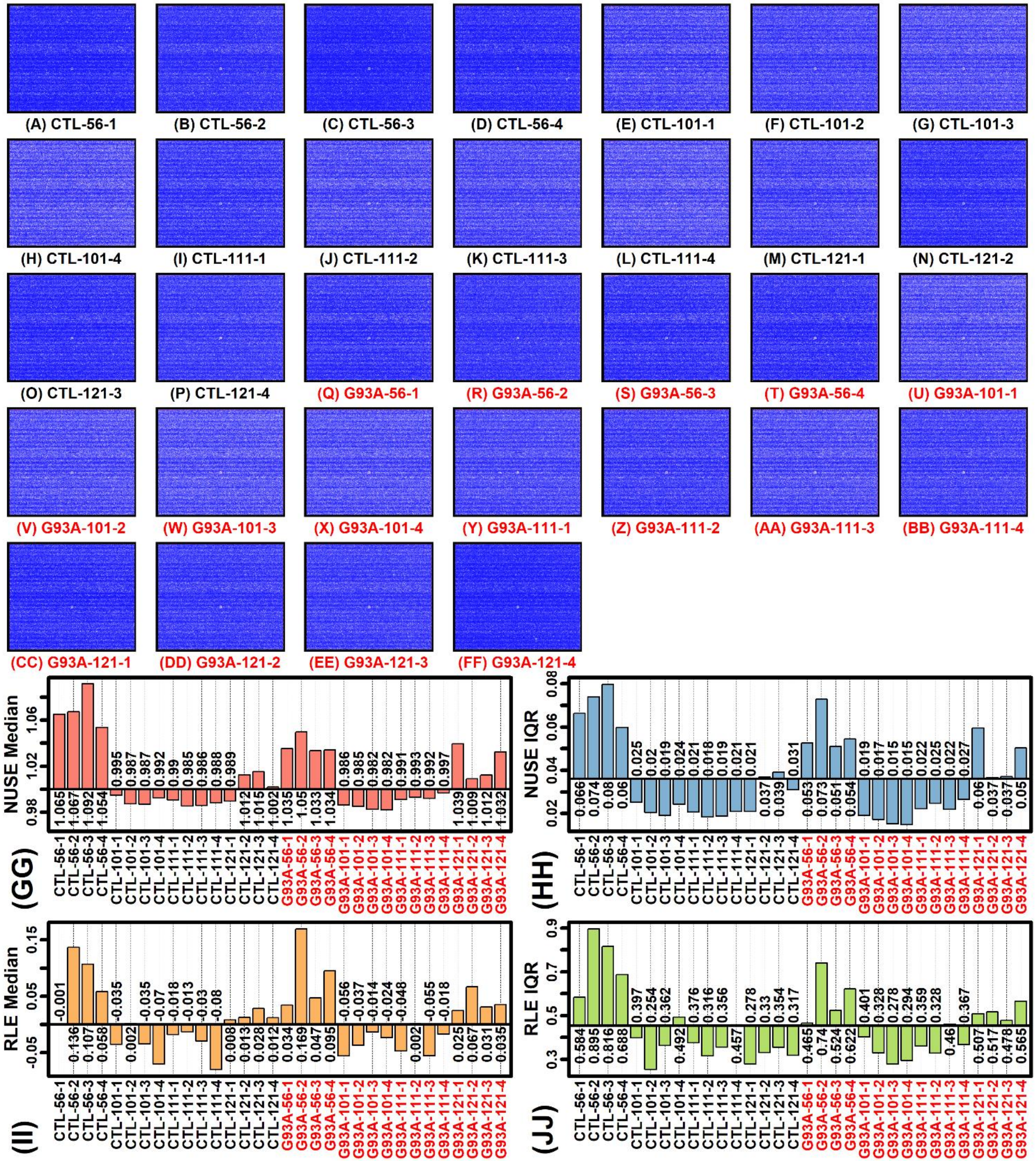

Figure S11. Microarray quality control (GSE46298, 129Sv). (A - FF) Microarray pseudoimages. (GG, HH) Normalized unscaled standard error (NUSE) median and interquartile range. (II, JJ) Relative log expression (RLE) median and interquartile range.

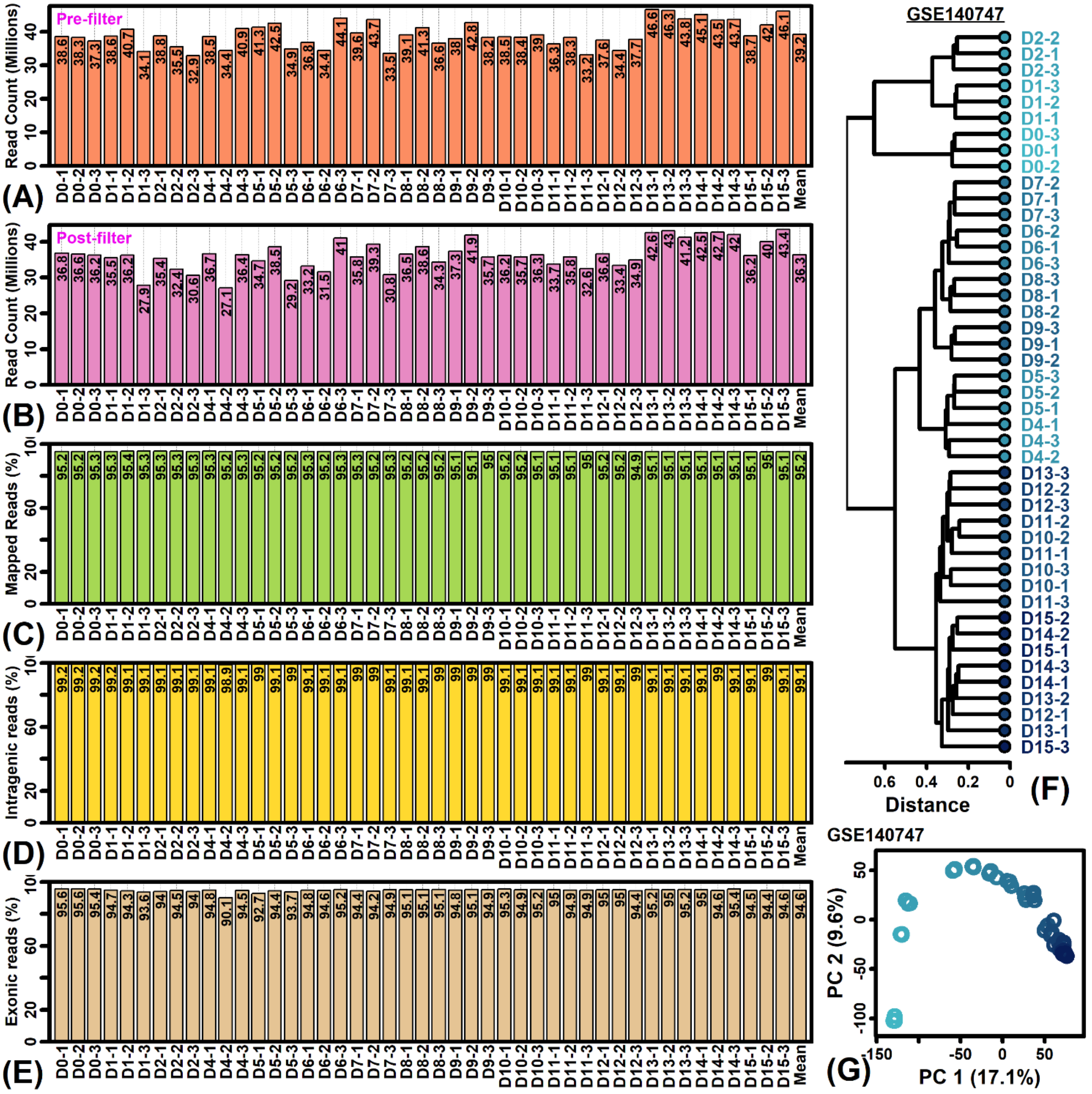

Figure S12. RNA-seq quality control, cluster and principal component analyses (GSE140747). (A) Read count (pre-filter). (B) Read count (post-filter). (C) Percentage of mapped reads. (D) Percentage of reads mapped to intragenic regions. (E) Percentage of reads mapped to exonic regions. (F) Hierarchical cluster analysis. Samples were clustered based upon the Euclidean distance with average linkage. Labels indicate the day of the differentiation protocol (0 to 15) and replicate number (1 to 3). A darker label font is used for more differentiated samples (later in the time series). (G) Principal component scatterplot. Samples are plotted with respect to the first two PC axes. Symbols with darker font correspond to more differentiated samples collected later in the time series (see label color in part F above).

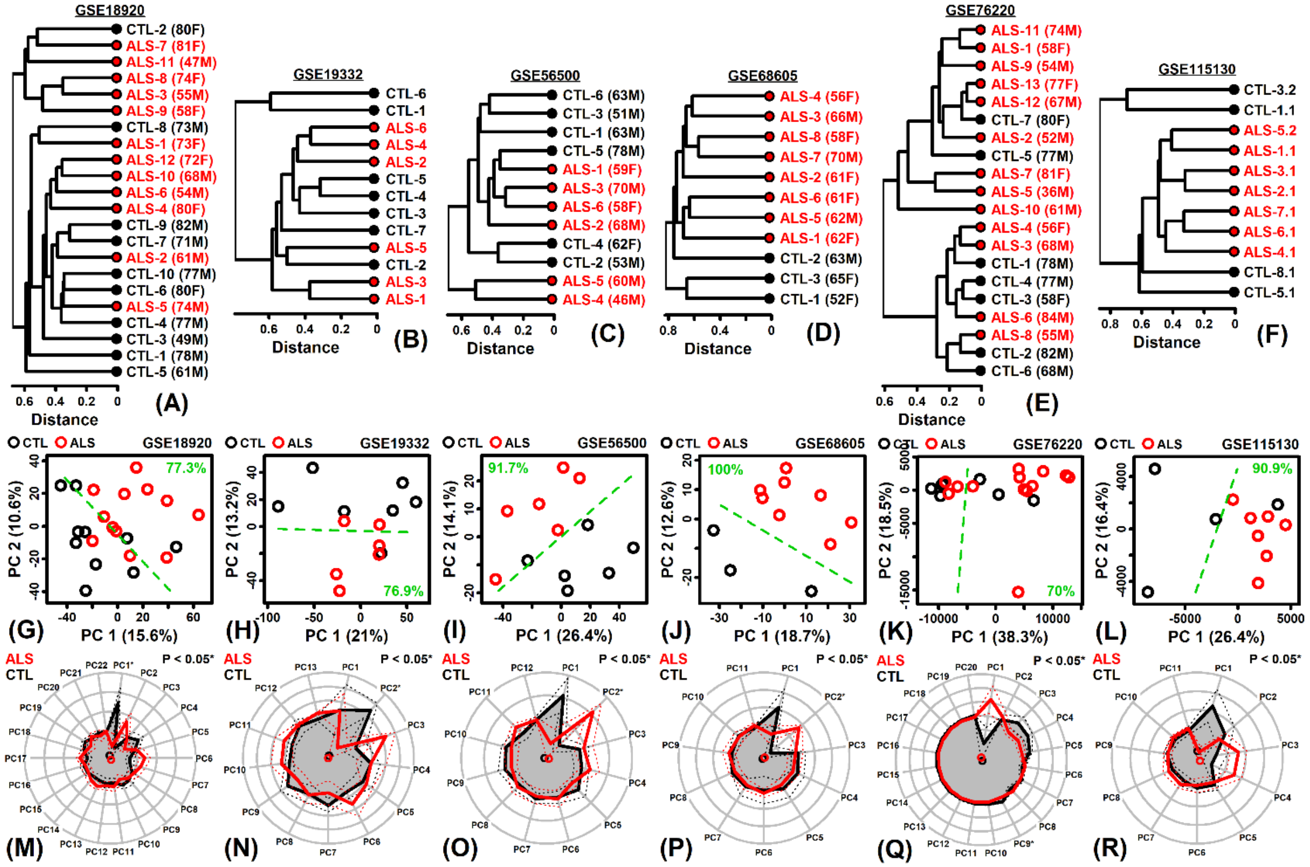

Figure S13. Cluster and principal component analyses. (A - F) Hierarchical cluster analyses. Samples were clustered based upon Euclidean distance with average linkage. (G - H) Principal component scatterplots. Samples are plotted with respect to the first two PC axes. The dotted green line represents the linear discriminant function separating ALS from CTL samples. The percentage of samples accurately classified (ALS vs. CTL) using the discriminant function is shown (green font). (M - R) Principal component radial plots. Average PC values for ALS (red) and CTL (black) samples are plotted (thick lines) ± 1 standard error (dotted lines). PC axes with significant differences between ALS and CTL sample scores are indicated (*, P < 0.05, two-sample two-tailed t-test).

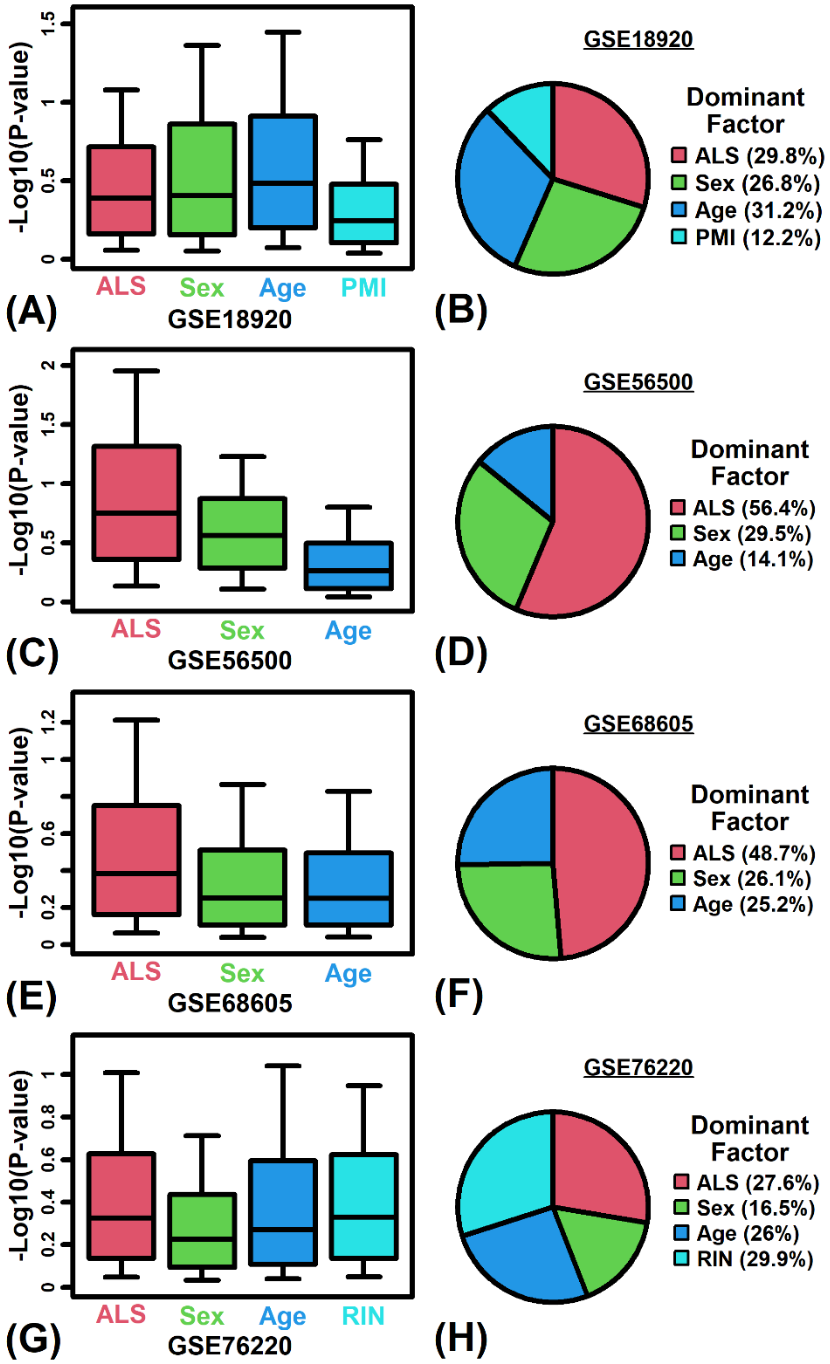

Figure S14. Factors associated with gene expression variation. (A, C, E, G) Likelihood ratio test (LRT) p-values (log10-transformed). LRTs were performed for each gene to compare full models (expression = ALS + sex + age ± PMI ± RIN) with reduced models (only 2-3 explanatory variables). Boxplots show the distribution of LRT p-values (log10-transformed) among genes when the indicated factor was eliminated from the full model. Boxes outline the middle 50% of log10-transformed p-values (whiskers: 10th to 90th percentiles). (B, D, F, H) Dominant factor distribution. The pie chart indicates the percentage of genes for which the indicated factor was dominant (i.e., greatest increase in deviance, full model vs. reduced model).

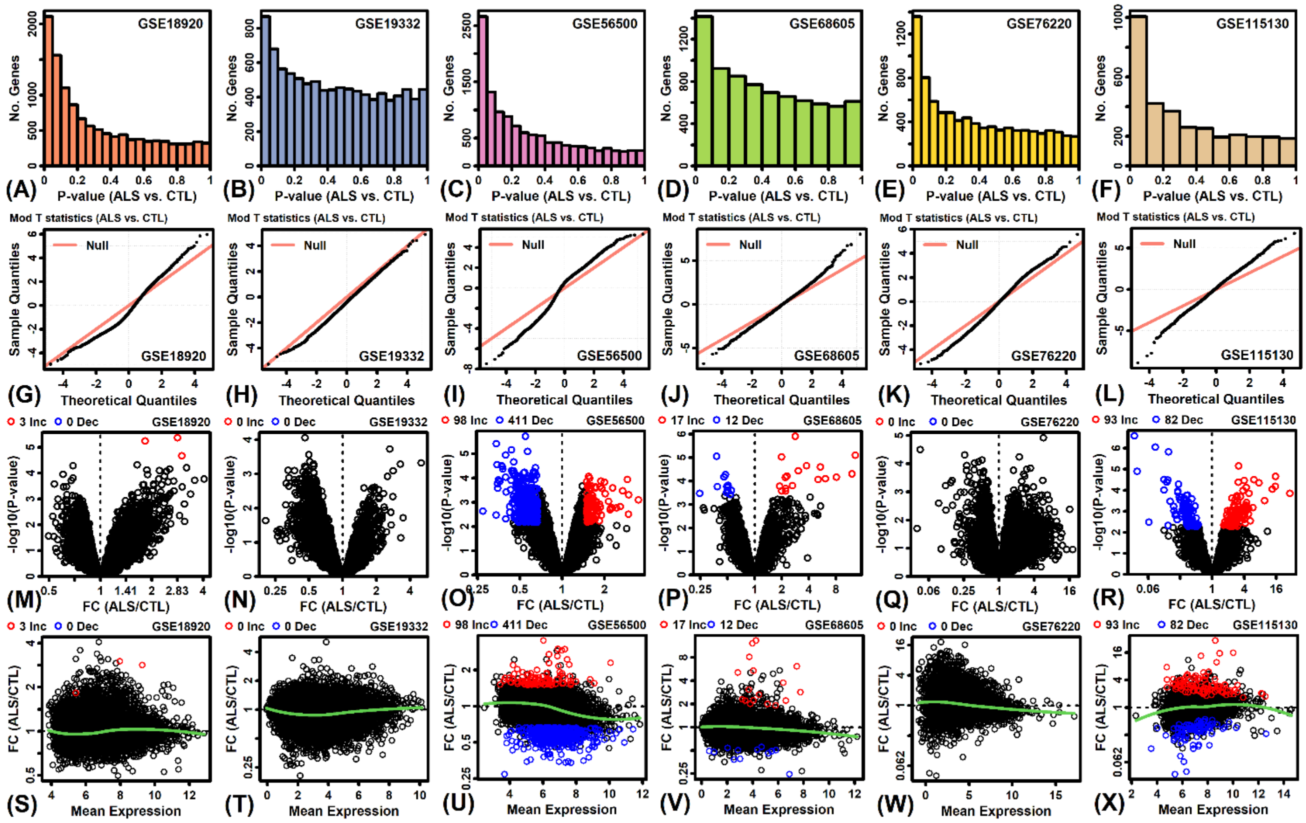

Figure S15. Differential expression analyses. (A - F) Raw p-value histograms. The distribution of raw p-values is shown among the motor neuron-expressed protein-coding genes included in each differential expression analysis. (G - L) Q-Q plots. Observed t statistic sample quantiles (vertical axis) are plotted against theoretical quantiles of Student's t distribution (horizontal axis). A larger proportion of differentially expressed genes is suggested by deviation from a straight line. (M - R) Volcano plots. The -log10-transformed p-values (vertical axis) are plotted against FC estimates (horizontal axis). (S - X) MA plots. FC estimates (vertical axis) are plotted against average normalized expression of genes (vertical axis). The green line represents the nonparametric locally weighted smoothing estimate (loess fit). In (M) - (X), each point represents an individual gene and colors denote ALS-increased (FDR < 0.10, FC > 1.50) and ALS-decreased (FDR < 0.10, FC < 0.67) genes. The number of differentially expressed genes is indicated (top margin).

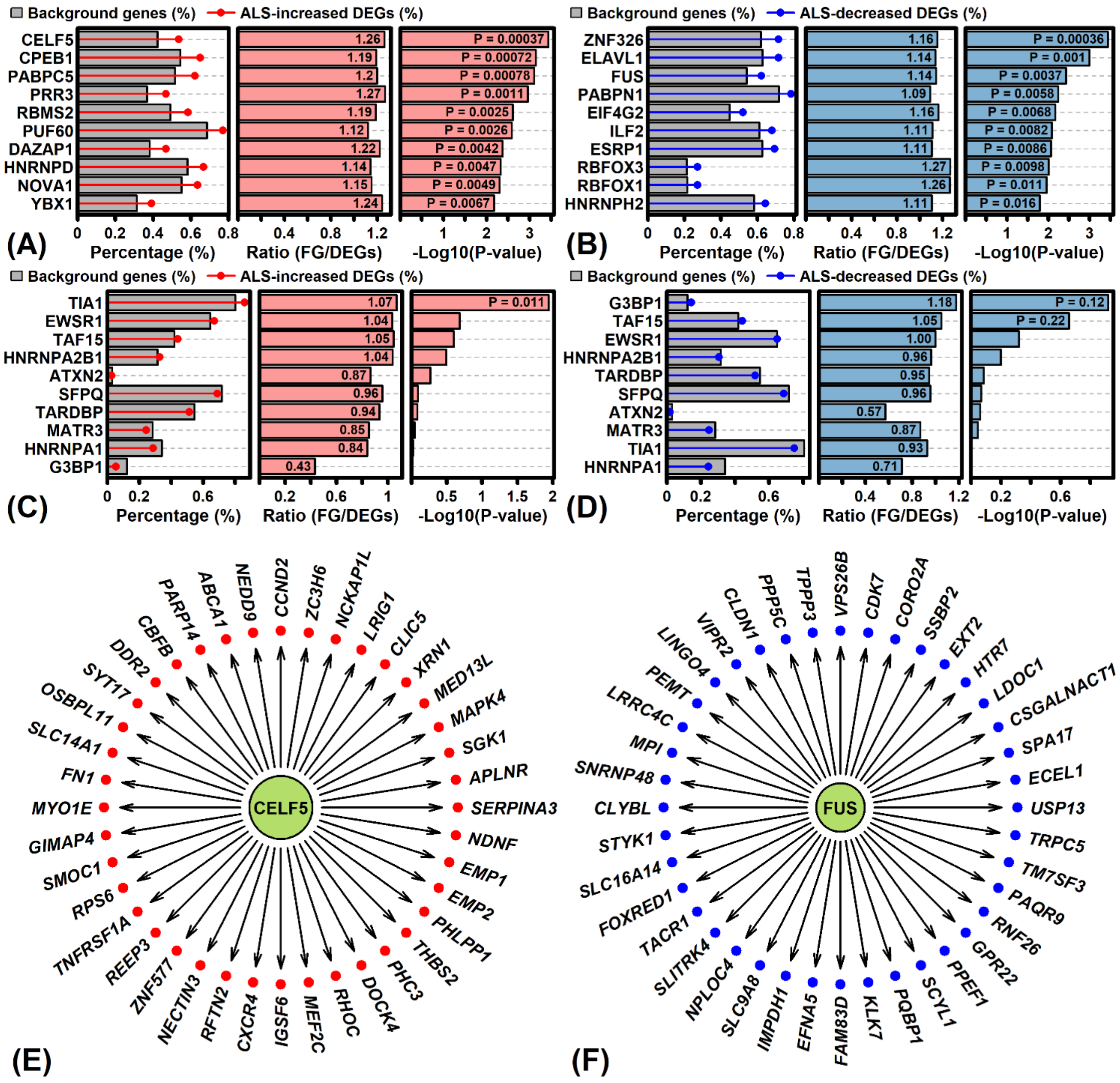

Figure S16. RNA-binding proteins interacting with mRNAs linked to DEGs (RNAInter database). (A, B) Top-ranked proteins overrepresented as interaction partners with mRNAs associated with (A) ALS-increased DEGs and (B) ALS-decreased DEGs. (C, D) Selected proteins and their frequency of interaction with mRNAs associated with (C) ALS-increased DEGs and (D) ALS-decreased DEGs. In (A) - (D), the percentage of DEGs interacting with the indicated protein is compared to the percentage among background non-DEGs (left). The ratio of these percentages is shown (middle), where ratios > 1 indicate an above-background rate of mRNA-protein interactions among DEGs compared to non-DEGs. P-values generated from Fisher's exact test are shown (right), where p-values < 0.05 denote a significantly increased rate of protein-mRNA interactions among DEGs compared to non-DEGs (without multiple hypothesis test correction; FDR > 0.10 for all proteins). (E) CELF5 interactions. ALS-increased DEGs with CELF5-interacting mRNAs are shown. (F) FUS interactions. ALS-decreased DEGs with FUS-interacting mRNAs are shown.

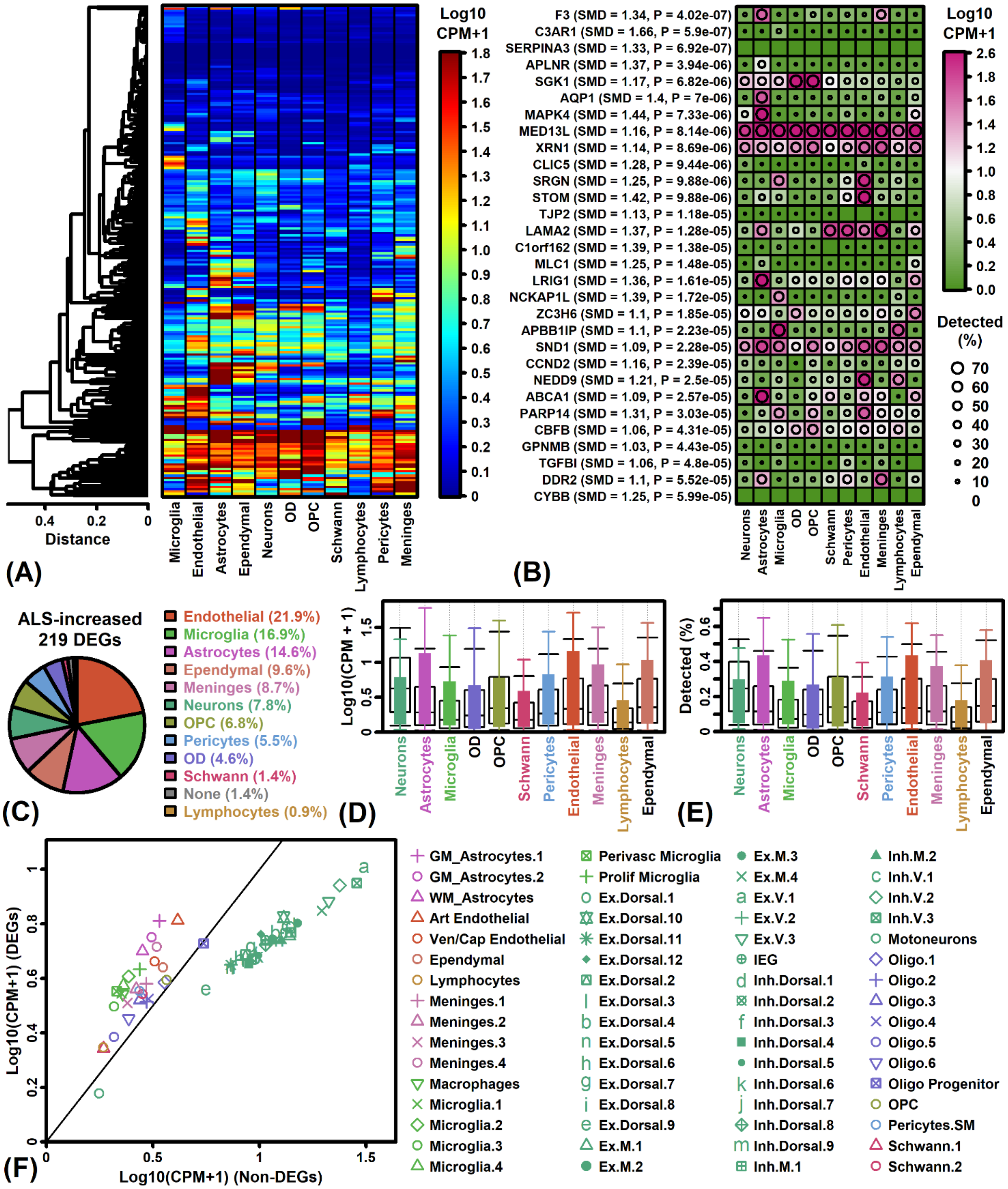

Figure S17. Expression of ALS-increased DEGs in cell types from normal adult spinal cord isolated by single-nucleus RNA sequencing (GSE190442). (A) Cluster analysis. The heatmap shows average expression for each cell type. Rows (genes) and columns (cell types) are clustered based upon the Euclidean distance metric (average linkage). (B) Top 30 ALS-increased DEGs. The heatmap shows average expression in each cell type (color) and the percentage of cells having detectable expression (i.e., CPM ≥ 1) (open circles). The SMD meta-estimate and p-value are listed

(left margin). (C) Pie chart. The 219 ALS-increased genes were assigned to the cell type for which average expression was highest. Genes without detectable expression in any cell type were assigned to the "None" category. (D, E) DEG vs. non-DEG expression by cell type. Colored boxplots correspond to ALS-increased DEGs, whereas non-colored boxplots correspond to all other meta-analysis genes. In (D), boxplots outline the middle 50% of average expression among genes (whiskers: 10th to 90th percentiles). In (E), the percentage of cells with detectable expression is evaluated (i.e., CPM $\geq$ 1) and boxplots outline the middle 50% of estimates among genes (whiskers: 10th to 90th percentiles). Colored labels (bottom margin) are used if median values differ significantly between DEGs and non-DEGs (FDR < 0.10, two-sample Wilcoxon rank sum test). (F) Spinal cord subpopulations. The scatterplot shows average expression of ALS-increased DEGs (vertical axis) and all other meta-analysis genes (horizontal axis) in 64 cell subpopulations. The 64 subpopulations are listed with their corresponding symbol (right margin). Subpopulation labels are consistent with those used by Yadav et al. 2023 (Neuron 111: 328-344).

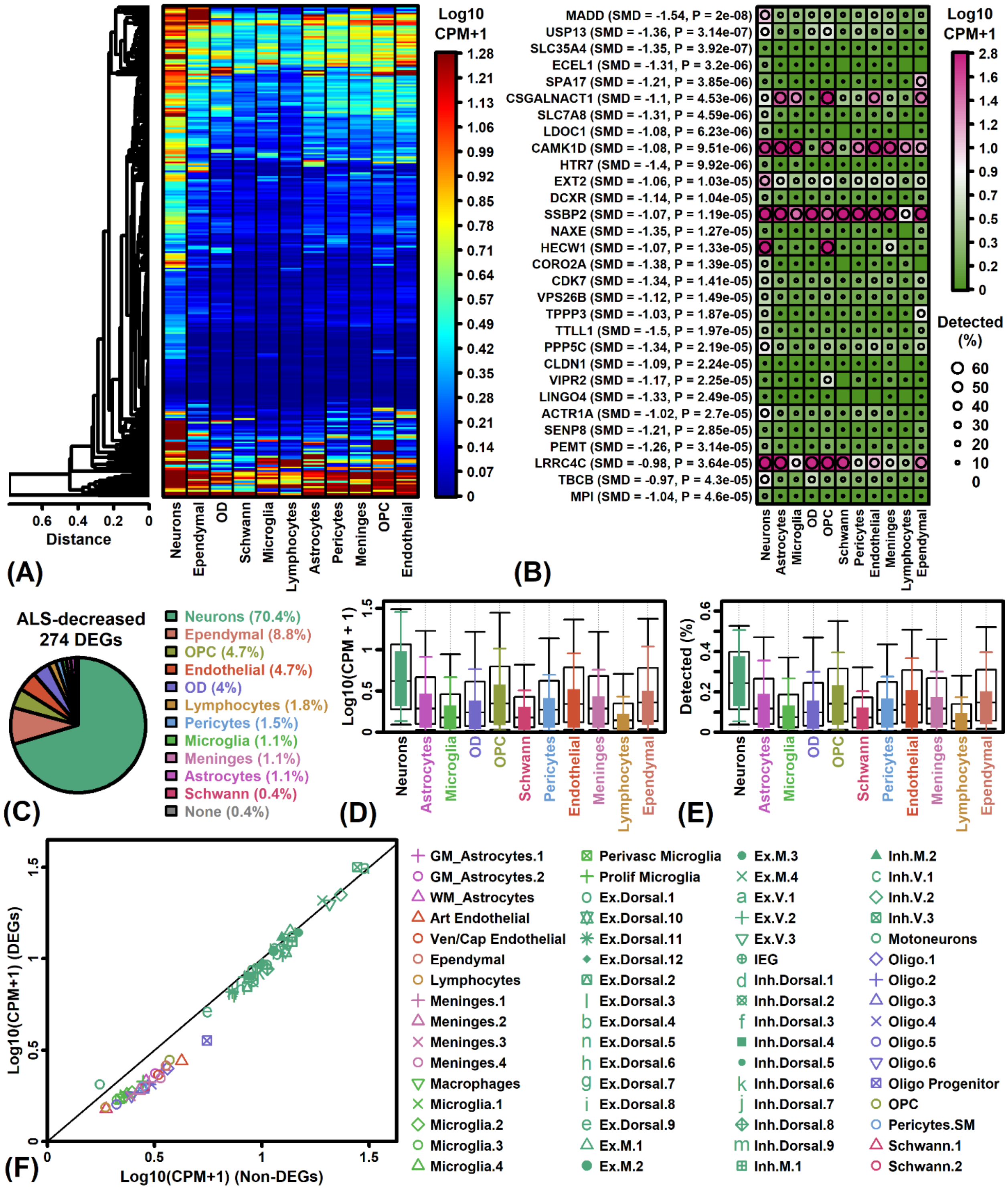

Figure S18. Expression of ALS-decreased DEGs in cell types from normal adult spinal cord isolated by single-nucleus RNA sequencing (GSE190442). (A) Cluster analysis. The heatmap shows average expression for each cell type. Rows (genes) and columns (cell types) are clustered based upon the Euclidean distance metric (average linkage). (B) Top 30 ALS-decreased DEGs. The heatmap shows average expression in each cell type (color) and the percentage of cells having

detectable expression (i.e., CPM ≥ 1) (open circles). The SMD meta-estimate and p-value are listed (left margin). (C) Pie chart. The 274 ALS-decreased genes were assigned to the cell type for which average expression was highest. Genes without detectable expression in any cell type were assigned to the "None" category. (D, E) DEG vs. non-DEG expression by cell type. Colored boxplots correspond to ALS-decreased DEGs, whereas non-colored boxplots correspond to all other meta-analysis genes. In (D), boxplots outline the middle 50% of average expression among genes (whiskers: 10th to 90th percentiles). In (E), the percentage of cells with detectable expression is evaluated (i.e., CPM ≥ 1) and boxplots outline the middle 50% of estimates among genes (whiskers: 10th to 90th percentiles). Colored labels (bottom margin) are used if median values differ significantly between DEGs and non-DEGs (FDR < 0.10, two-sample Wilcoxon rank sum test). (F) Spinal cord subpopulations. The scatterplot shows average expression of ALS-decreased DEGs (vertical axis) and all other meta-analysis genes (horizontal axis) in 64 cell subpopulations. The 64 subpopulations are listed with their corresponding symbol (right margin). Subpopulation labels are consistent with those used by Yadav et al. 2023 (Neuron 111: 328-344).

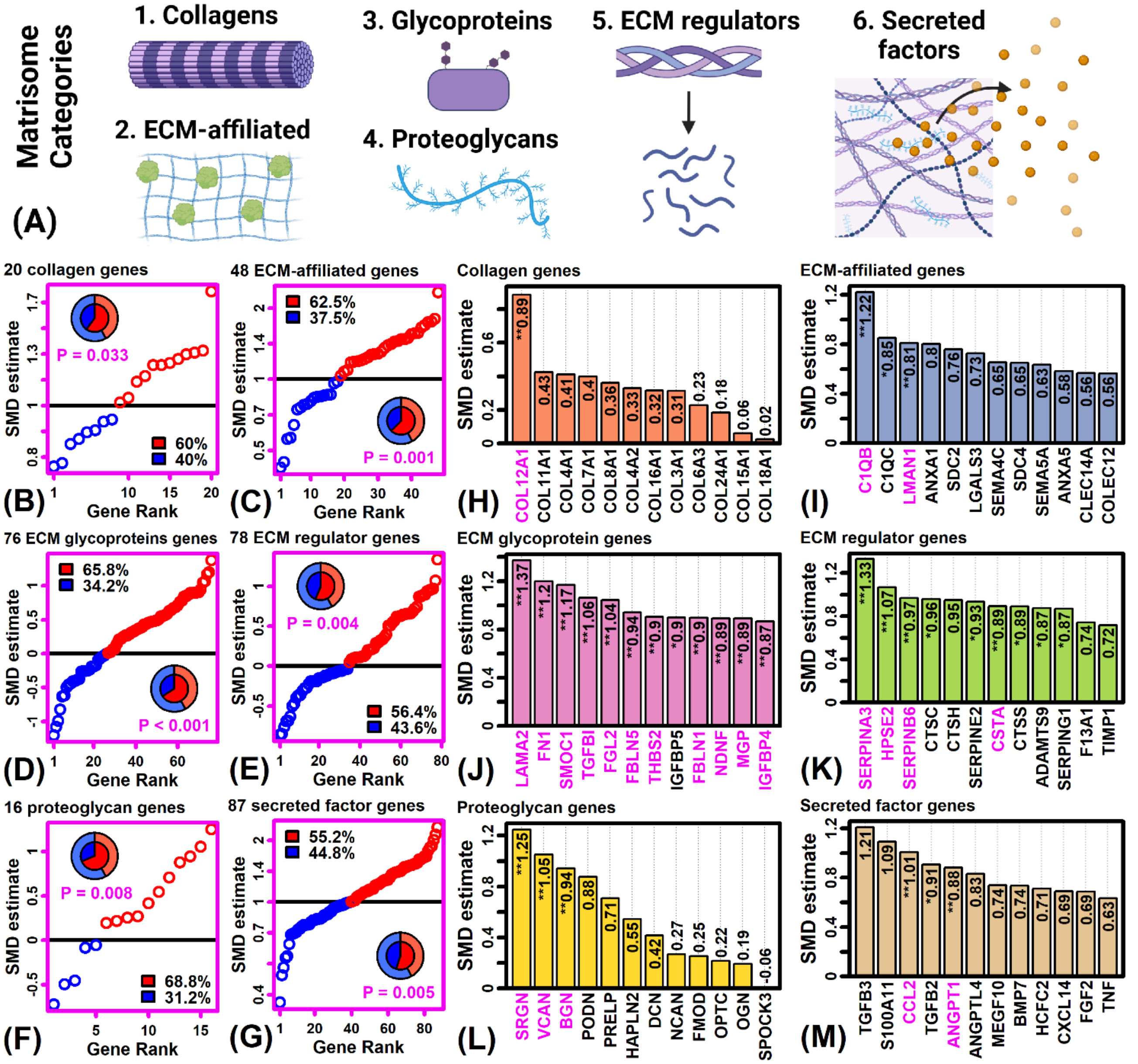

Figure S19. Matrisome genes. (A) Matrisome gene categories. (B - G) SMD estimates for genes in each matrisome category (red: ALS-increased; blue: ALS-decreased). The number of motor neuron-expressed genes in each category is indicated (top margin). The percentage of ALS-increased and ALS-decreased genes is shown (legend) and represented by the pie chart (inner region: genes within each matrisome category; outer region: all 9882 motor neuron-expressed genes). The p-value was obtained by testing whether inner and outer pie chart frequencies differ significantly (Fisher's exact test). (H - M) ALS-increased genes in each matrisome category. The SMD estimate is shown for each gene (*P < 0.05; **FDR < 0.10, gene symbol in magenta font).

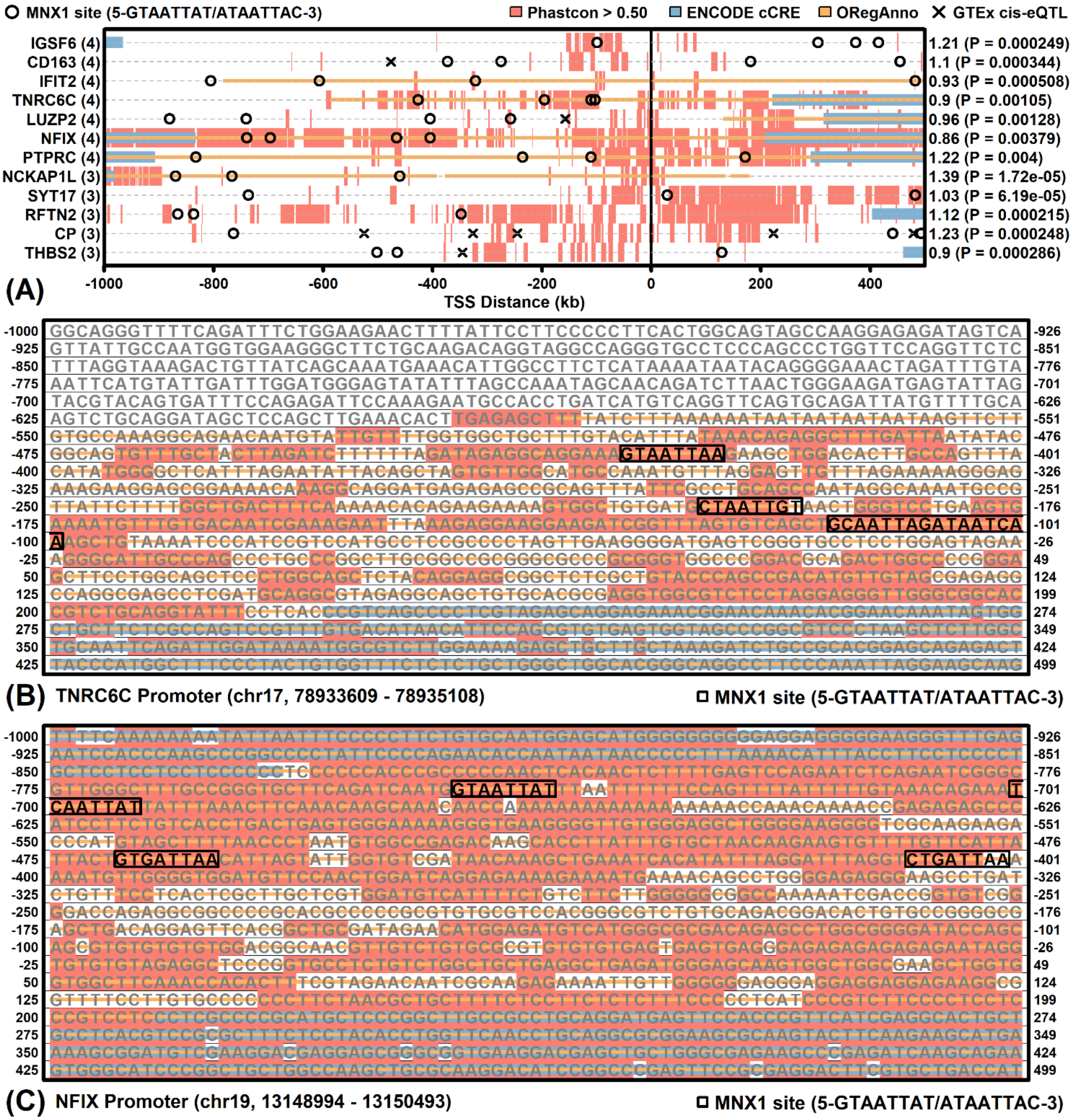

Figure S20. ALS-increased genes with the highest number of MNX1 motif matches in TSS-proximal sequences. (A) Top 12 ALS-increased genes. Gene symbols are shown with the number of MNX1 motif matches (5-GTAATTAT/ATAATTAC-3) in parentheses (left margin). The 1000 bp upstream and 500 bp downstream sequences are plotted with symbols and background regions as indicated (see figure legend, top margin). The SMD meta-estimate is given with p-value (right margin). (B, C) TSS-proximal sequences for (B) trinucleotide repeat containing adaptor 6C (*TNRC6C*) and (C) nuclear factor I X (*NFIX*). Background colors are as indicated in the part (A) legend and boxes are drawn around MNX1 motif matches. Sequences are shown in 5’ to 3’ orientation.

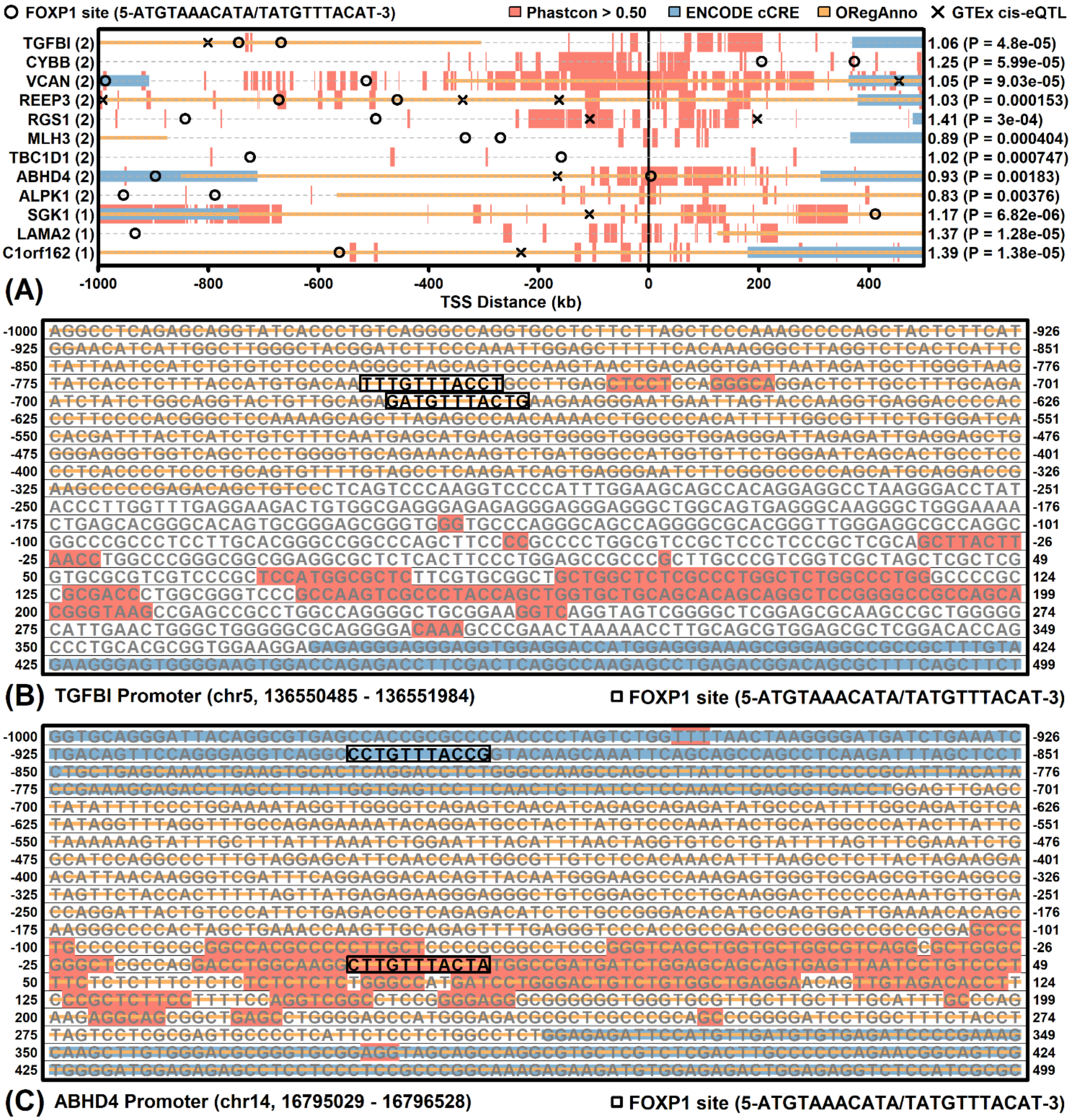

Figure S21. ALS-increased genes with the highest number of FOXP1 motif matches in TSS-proximal sequences. (A) Top 12 ALS-increased genes. Gene symbols are shown with the number of FOXP1 motif matches (5-ATGTAAACATA/TATGTTTACAT-3) in parentheses (left margin). The 1000 bp upstream and 500 bp downstream sequences are plotted with symbols and background regions as indicated (see figure legend, top margin). The SMD meta-estimate is given with p-value (right margin). (B, C) TSS-proximal sequences for (B) transforming growth factor beta induced (*TGFBI*) and (C) abhydrolase domain containing 4 N-acyl phospholipase B (*ABHD4*). Background colors are as indicated in the part (A) legend and boxes are drawn around FOXP1 motif matches. Sequences are shown in 5’ to 3’ orientation.

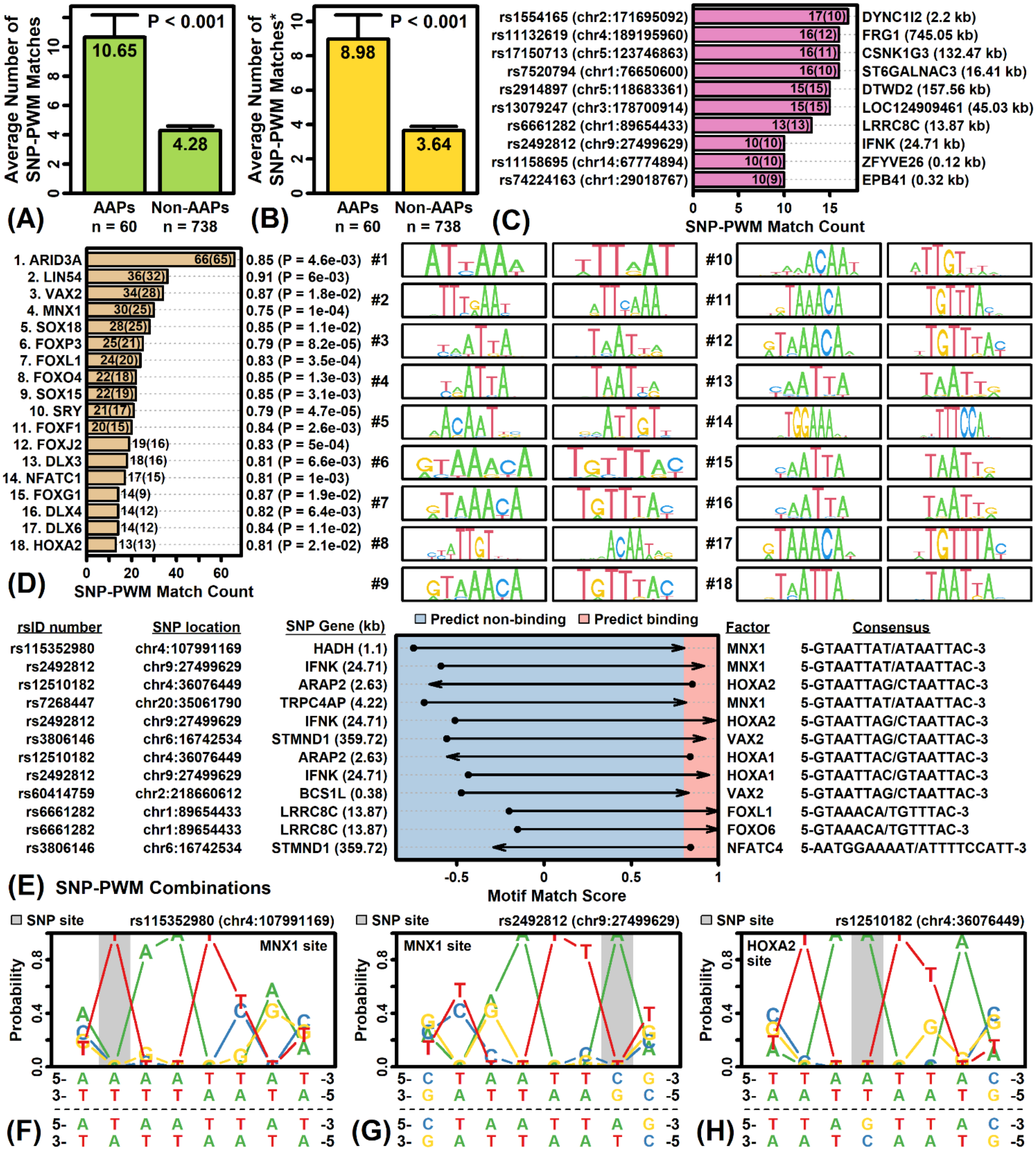

Figure S22. ALS-associated SNP variants and their effects on genomic matches to ALS-associated PWM models. (A, B) Average number of SNP-PWM matches among ALS-associated PWMs (AAPs) and PWMs not associated with ALS (non-AAPs). The average is shown for both PWM groups (± 1 standard error). Part (A) includes genotype-dependent and -independent PWM matches whereas part (B) includes only genotype-independent matches where both SNP variants matched a given PWM model. (C) ALS-associated SNP loci having the largest number of matches to

ALS-associated PWM models. The SNP site is listed (left margin) along with the nearest gene and its genomic distance to the SNP (right margin). Bars indicate the total number of SNP-PWM matches for a given SNP (genotype-dependent and -independent). The number of genotype-dependent SNP-PWM matches is given in parentheses. (D) ALS-associated PWM models having the largest number of matches at ALS-associated SNP loci. The transcription factor associated with each PWM model is listed (left margin). Bars indicate the total number of SNP-PWM matches for the indicated PWM model (genotype-dependent and -independent). The number of genotype-dependent SNP-PWM matches is given in parentheses. The right margin lists the ratio statistic for each PWM model (i.e., average motif-TSS distance of ALS-increased DEGs / average motif-TSS distance for all other genes) and its associated p-value. Sequence logos for each PWM model are shown (far right). (E) Enhancer-associated genotype-dependent SNP-PWM matches. SNP (left margin) and PWM model information (right margin) is shown for each SNP-PWM match. The PWM match score is plotted for both SNP variants (closed circle: reference genome; arrow: non-reference genome). (F-G) Top 3 SNP-PWM pairs from part (E). The position probability matrix for each PWM is plotted with different colors used for each letter. Two genomic sequences are shown (bottom margin) differing only with respect to the SNP locus (top: reference genome; bottom: non-reference genome).

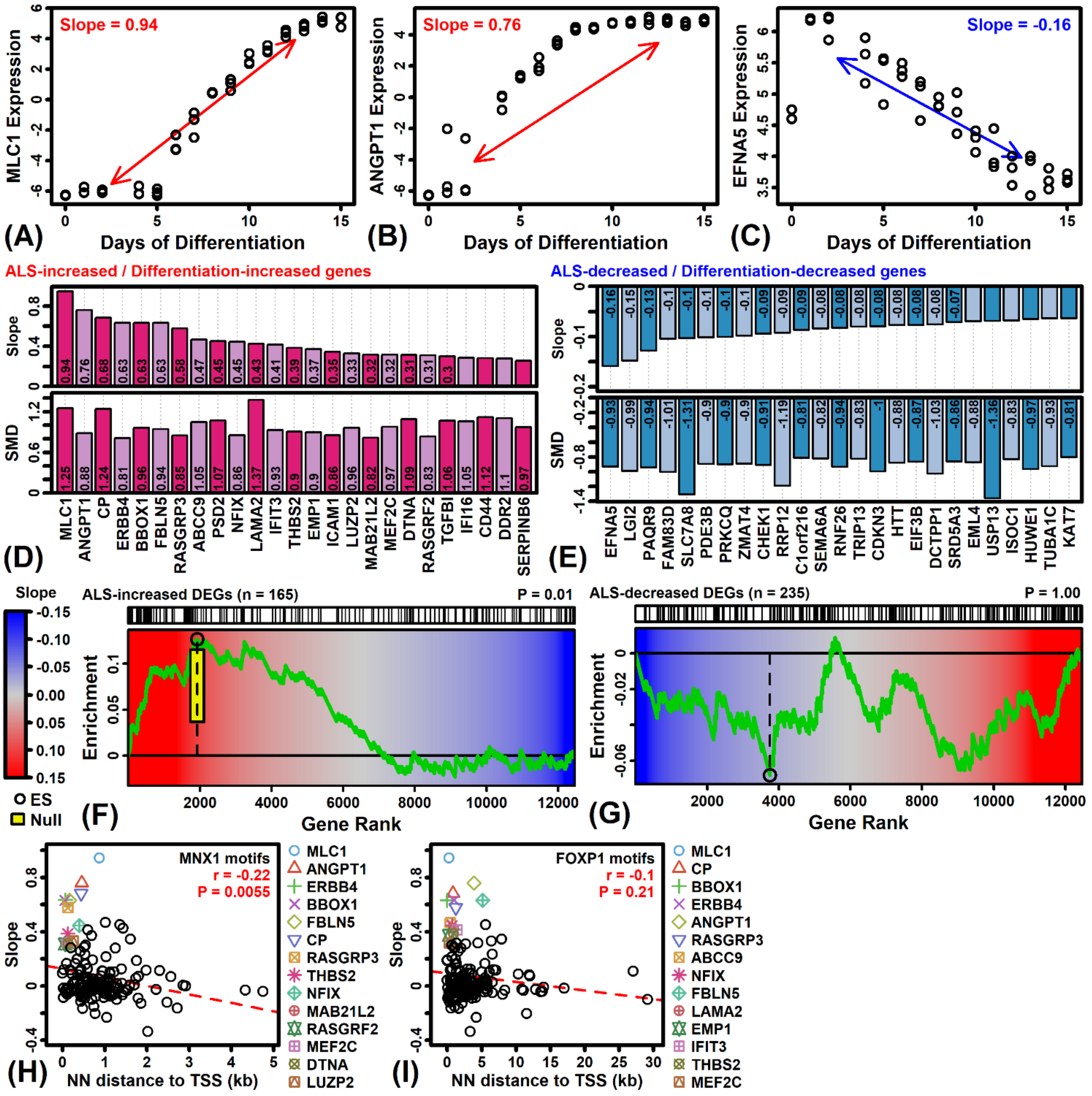

Figure S23. Comparison of ALS DEGs to genes altered during *in vitro* motor neuron differentiation (GSE140747). (A) Modulator of VRAC current 1 (*MLC1*) expression. (B) Angiopoietin 1 (*ANGPT1*) expression. (C) Ephrin A5 (*EFNA5*) expression. In (A) - (C), voom-normalized expression values (log_2 scale) are shown and the slope estimated by least squares regression is given (double-arrowed line). (D) ALS-increased / differentiation-increased genes. The meta-analysis SMD estimate is shown (bottom) with least squares slope estimated from the differentiation time series (top). All genes shown were increased during the differentiation time series (FDR < 0.10) and increased in ALS LCM-dissected motor neurons (FDR < 0.10 with SMD > 0.80). (E) ALS-decreased / differentiation-decreased genes. The meta-analysis SMD estimate is shown (bottom) along with least squares slope estimated from the differentiation time series (top). All genes shown were decreased during the differentiation time series (FDR < 0.10) and decreased in ALS

LCM-dissected motor neurons (FDR < 0.10 with SMD > 0.80). (F, G) Gene set enrichment analyses (GSEA). Genes were ranked based upon the estimated slope (see color scale) and a running sum score was tabulated (green line) based on the position of (F) ALS-increased or (G) ALS-decreased genes within the ranked gene list. The enrichment score (black circle) is identified as the running sum score with maximum absolute value. The yellow box outlines the middle 95% of the enrichment score null distribution obtained in simulations wherein the ranked gene list was randomly permuted (100000 trials). (H, I) Relationship between slope estimates and nearest neighbor (NN) distances between ALS-increased DEGs and (H) MNX1 or (I) FOXP1 motifs. Each symbol represents an ALS-increased DEG and the dashed red line represents the least-squares regression estimate. The Spearman correlation coefficient and associated p-value are shown (upper right). ALS-increased DEGs closest to a MNX1/FOXP1 motif with highest slope estimates are highlighted using special symbols (right margin).

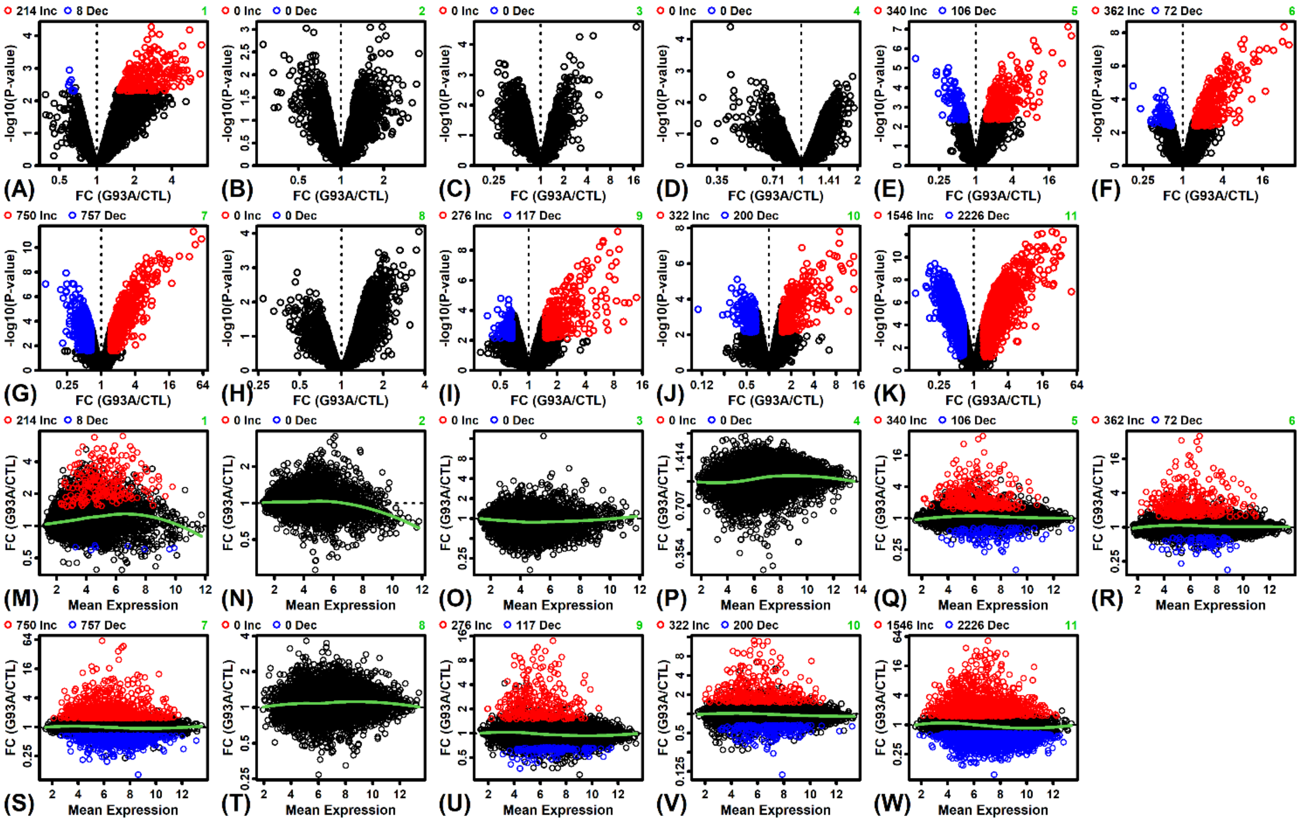

Figure S24. Differential expression analyses (GSE10953 and GSE46298). (A - K) Volcano plots. The -log10-transformed p-values (vertical axis) are plotted against FC estimates (horizontal axis). (M - W) MA plots. FC estimates (vertical axis) are plotted against average normalized expression of genes (vertical axis). The green line represents the nonparametric locally weighted smoothing estimate (loess fit). In (A) - (W), each point represents an individual gene and colors denote SOD1-G93A-increased (FDR < 0.10, FC > 1.50) and SOD1-G93A-decreased (FDR < 0.10, FC < 0.67) genes. The number of differentially expressed genes is indicated along with the comparison identifier (top margin).

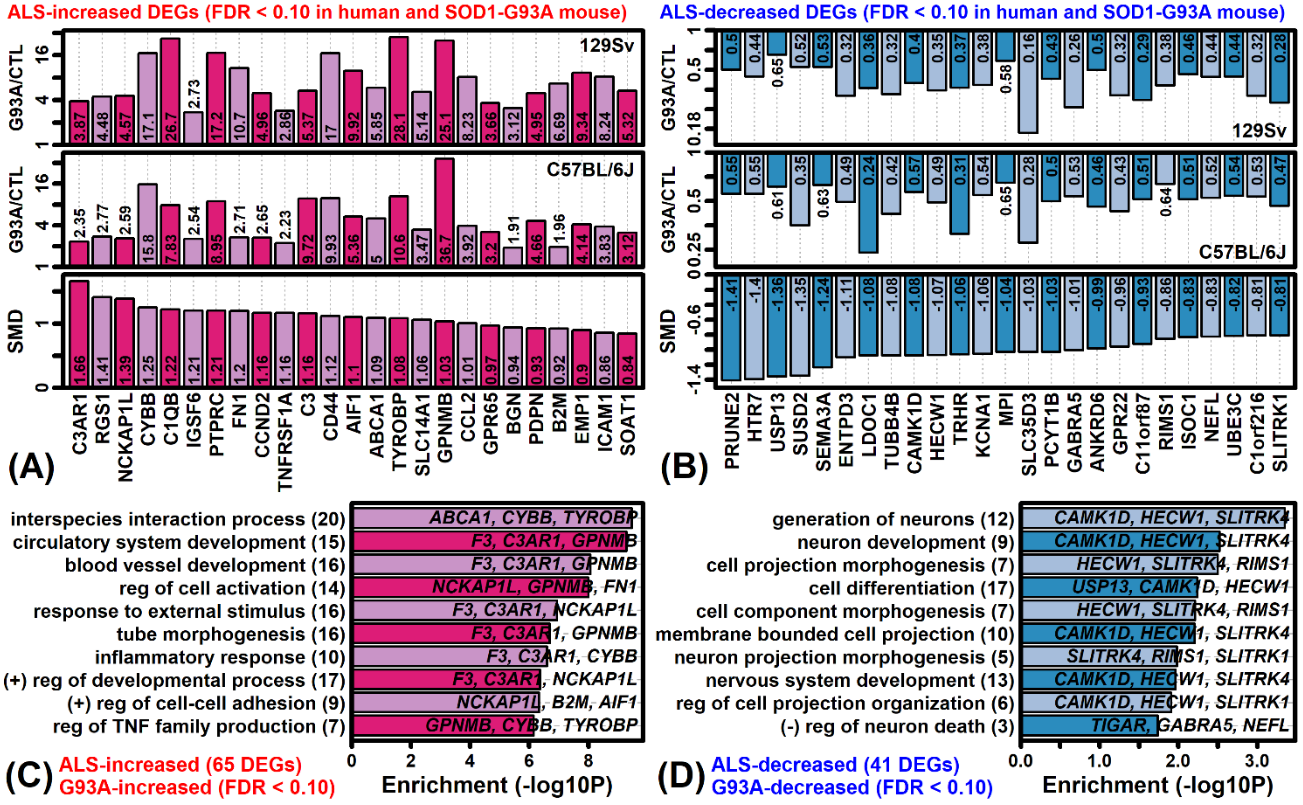

Figure S25. ALS DEGs similarly altered in LCM-dissected motor neurons from SOD1-G93A mice (endstage phenotype, GSE46298). (A) ALS-increased DEGs having increased expression in SOD1-G93A mouse motor neurons. The meta-analysis SMD estimate is shown on the bottom panel and FC estimates (G93A/CTL) are shown for two strains. All genes had significantly increased expression in G93A mice from both strains (FDR < 0.10 with FC > 1.50). (B) ALS-decreased DEGs having decreased expression in SOD1-G93A mouse motor neurons. The meta-analysis SMD estimate is shown on the bottom panel and FC estimates (G93A/CTL) are shown for two strains. All genes had significantly decreased expression in G93A mice from both strains (FDR < 0.10 with FC < 0.67). (C, D) GO BP terms. Figures show GO BP terms enriched among (C) ALS-increased DEGs with increased expression in G93A motor neurons and (D) ALS-decreased DEGs with decreased expression in G93A motor neurons. The number of ALS DEGs associated with each GO BP term is given in parentheses (left margin) and example genes are listed within each figure.

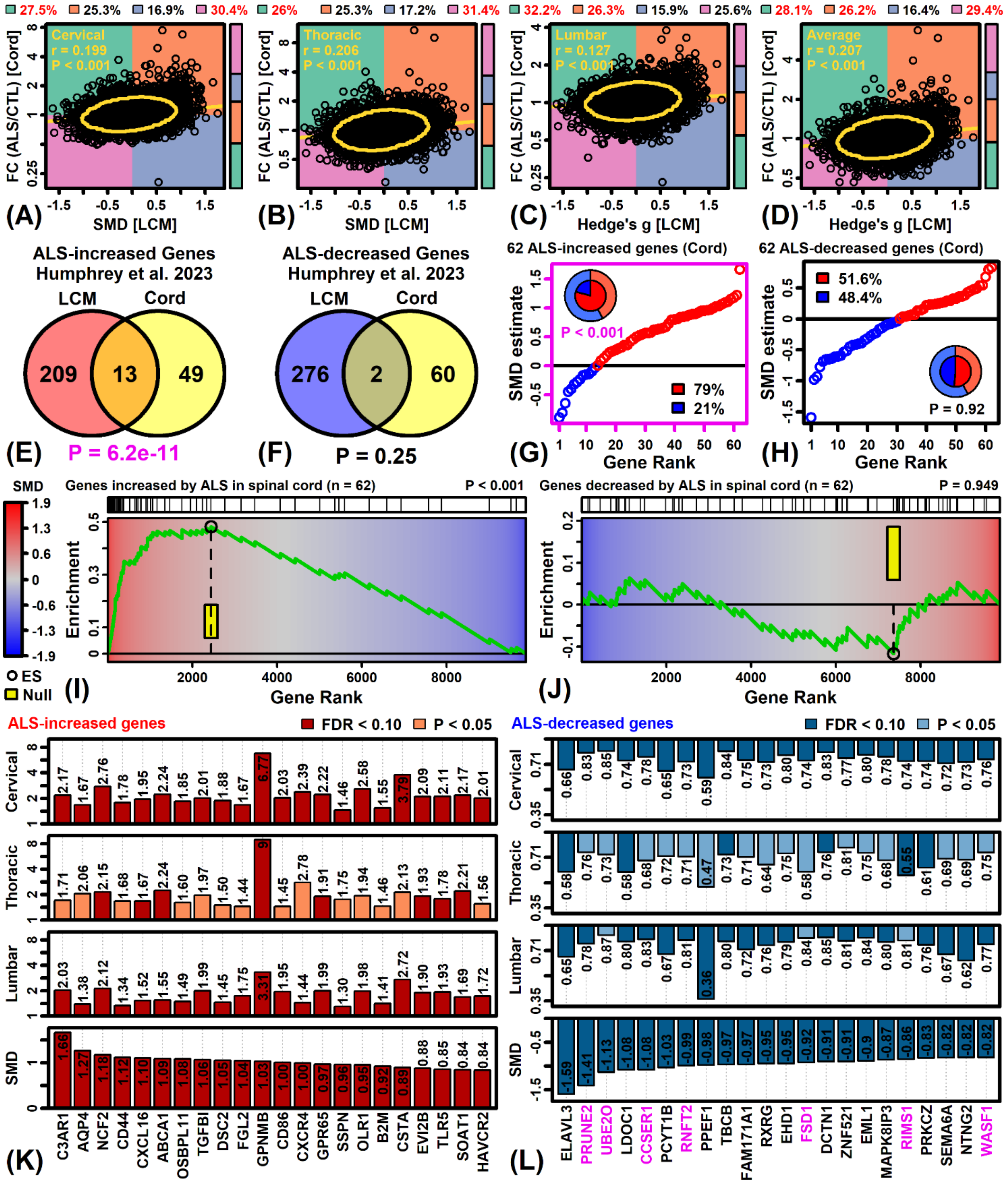

Figure S26. Comparison between LCM-dissected and post-mortem bulk spinal cord-derived ALS signatures (New York Genome Center ALS Consortium). (A - D) SMD estimates (LCM-dissected motor neurons) are compared to FC estimates (ALS vs. CTL) from bulk spinal cord segments, including (A) cervical cord, (B) thoracic cord, (C) lumbar cord or (D) the average of all

three cord regions. Each point represents an individual gene. The proportion of genes within each quadrant is shown and Pearson's chi-squared test is used to determine if the proportion of genes in any quadrant exceeds 25% (top margin; red font, $P < 0.05$). The right sidebar graphically represents the proportions in each quadrant. The Spearman correlation coefficient is shown with p-value (upper left). The yellow straight line is calculated by least-squares regression. A yellow ellipse is drawn around the middle 90% of genes closest to the bivariate mean (Mahalanobis distance). (E, F) Venn diagrams. Overlap is shown between genes altered in the LCM meta-analysis (left) and bulk spinal cord (right). Genes increased in ALS spinal cord segments were selected by identifying genes with FC > 1.50 in each cord region, with FDR < 0.10 in at least one region and $P < 0.05$ in all 3 regions. Likewise, genes decreased in ALS cord segments were selected by identifying genes with FC < 0.67 in each cord region, with FDR < 0.10 in at least one region and $P < 0.05$ in all 3 regions. (G, H) SMD estimates observed for genes altered in ALS vs. CTL spinal cords (based on above-stated FC, FDR and p-value criteria). Rank-ordered symbols represent SMD estimates for each gene (red: ALS-increased, LCM samples; blue: ALS-decreased, LCM samples). The percentage of ALS-increased and ALS-decreased genes is shown (legend) and represented by the pie chart (inner region: the 62 ALS-increased or -decreased genes in spinal cord; outer region: all 9882 motor neuron-expressed genes). The p-value was obtained by testing whether the inner and outer pie chart frequencies differ significantly (Fisher's exact test). (I, J) Gene set enrichment analyses. Genes were ranked based upon the SMD estimate (see color scale) and a running sum score was tabulated (green line) based on the position of genes associated with (I) genes increased in ALS spinal cords or (J) genes decreased in ALS spinal cords. The enrichment score (black circle) is identified as the running sum score with maximum absolute value. The yellow box outlines the middle 95% of the enrichment score null distribution from simulations in which the ranked gene list was randomly permuted (100000 trials). (K, L) Genes similarly altered in LCM-dissected motor neurons (meta-analysis) and whole spinal cord (ALS vs. CTL). FC estimates for each spinal cord region are indicated in the top 3 rows with SMD estimates (bottom). In (L), genes in magenta font belong to the M20 module (suspected to be motor neuron-related).

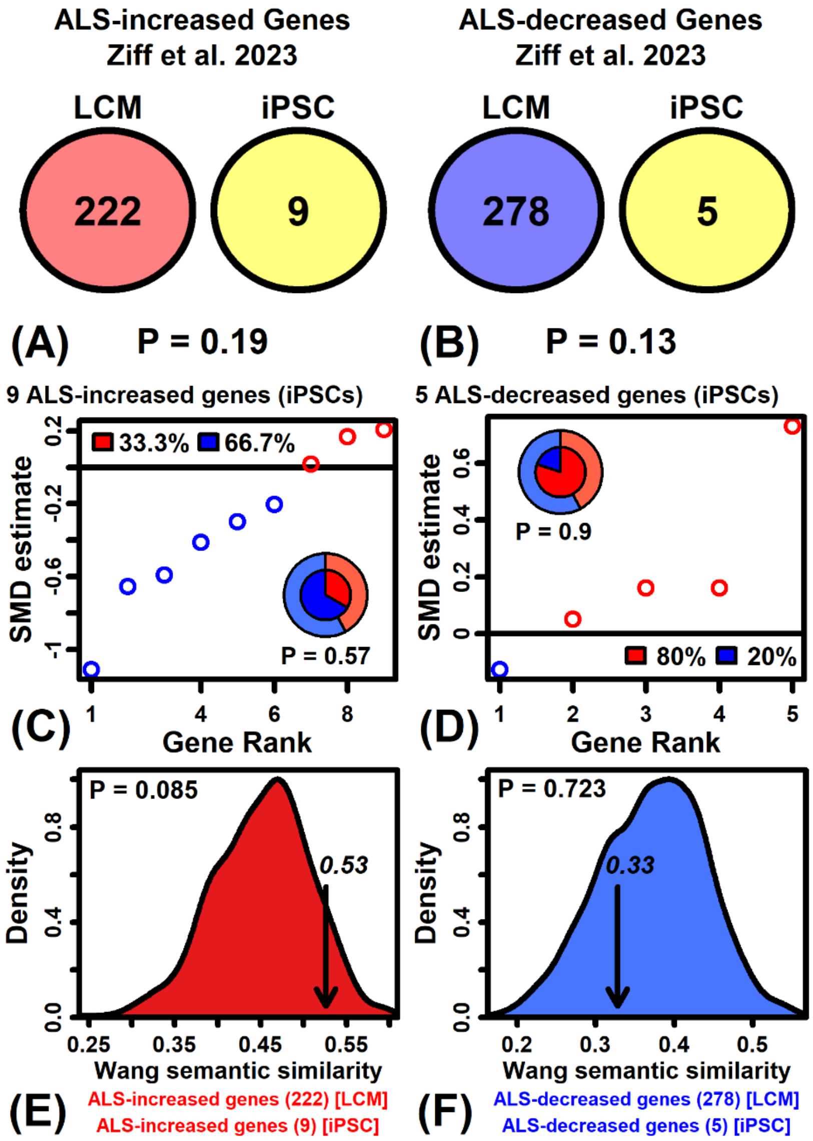

Figure S27. Comparison between LCM-dissected and iPSC-MN ALS signatures. The LCM-dissected motor neuron signature (ALS vs. CTL) was calculated based upon meta-analysis (i.e., SMD estimates). The iPSC-MN signature (ALS vs. CTL) was obtained from analysis of iPSC-MN samples reported previously (see text). (A, B) Venn diagrams. In (A), overlap is shown between ALS-increased genes from LCM-dissected motor neurons (SMD > 0.80, FDR < 0.10) and ALS-increased genes from iPSC-MNs (FDR < 0.05). In (B), overlap is shown between ALS-decreased genes from LCM-dissected motor neurons (SMD < 0.80, FDR < 0.10) and ALS-decreased genes from iPSC-MNs (FDR < 0.05). Fisher's exact test is used to evaluate the significance of overlap (bottom margin p-values). (C, D) SMD estimates observed for genes with altered expression in iPSC-MNs. Rank-ordered symbols represent SMD for each gene (red: increased in LCM-dissected ALS samples; blue: decreased in LCM-dissected ALS samples). The percentage of ALS-increased and ALS-decreased genes is shown (legend) and represented by the pie chart (inner region: the 9 or 5 genes with altered expression in iPSC-MN samples; outer region: all 9882 motor neuron-expressed genes). The p-value was obtained by testing whether the inner and outer pie chart frequencies differ significantly (Fisher’s exact test). (E, F) Pairwise GO semantic similarity (Wang metric). The pairwise similarity

(arrow) was calculated between the gene sets listed in the bottom margin. P-values were calculated based upon the null distribution (shown) generated from 1000 simulation trials. In each trial, motor neuron-expressed gene sets of the same size were generated by random sampling and the Wang similarity was calculated between the randomly generated sets.
